# Rational Design and Synthesis of a Novel Series of Thiosemicarbazone-Containing Quinazoline Derivatives as Potential VEGFR2 Inhibitors

**DOI:** 10.3390/pharmaceutics17020260

**Published:** 2025-02-15

**Authors:** Alexandru Șandor, Ovidiu Crișan, Gabriel Marc, Ionel Fizeșan, Ioana Ionuț, Cristina Moldovan, Anca Stana, Ilioara Oniga, Adrian Pîrnău, Laurian Vlase, Andreea-Elena Petru, Ionuț-Valentin Creștin, Alex-Robert Jîjie, Brîndușa Tiperciuc, Ovidiu Oniga

**Affiliations:** 1 Department of Pharmaceutical Chemistry, Faculty of Pharmacy, “Iuliu Hațieganu” University of Medicine and Pharmacy, 41 Victor Babes, Street, 400010 Cluj-Napoca, Romania; alexandru.sandor@elearn.umfcluj.ro (A.Ș.); ionut.ioana@umfcluj.ro (I.I.); cmoldovan@umfcluj.ro (C.M.); stana.anca@umfcluj.ro (A.S.); btiperciuc@umfcluj.ro (B.T.); ooniga@umfcluj.ro (O.O.); 2 Department of Organic Chemistry, “Iuliu Hațieganu” University of Medicine and Pharmacy, 41 Victor Babeş, 400012 Cluj-Napoca, Romania; marc.gabriel@umfcluj.ro; 3 Department of Toxicology, Faculty of Pharmacy, “Iuliu Hațieganu” University of Medicine and Pharmacy, 8 Victor Babeș Street, 400012 Cluj-Napoca, Romania; ionel.fizesan@umfcluj.ro (I.F.); andreea.elen.petru@elearn.umfcluj.ro (A.-E.P.); ionut.vale.crestin@elearn.umfcluj.ro (I.-V.C.); 4 Department of Pharmacognosy, “Iuliu Hațieganu” University of Medicine and Pharmacy, 12 Ion Creangă Street, 400010 Cluj-Napoca, Romania; ioniga@umfcluj.ro; 5 National Institute for Research and Development of Isotopic and Molecular Technologies, 67-103 Donath Street, 400293 Cluj-Napoca, Romania; apirnau@itim-cj.ro; 6 Department of Pharmaceutical Technology and Biopharmaceutics, “Iuliu Hațieganu” University of Medicine and Pharmacy, 41 Victor Babes, Street, 400012 Cluj-Napoca, Romania; laurian.vlase@umfcluj.ro; 7 Department of Toxicology, Faculty of Pharmacy, “Victor Babeș” University of Medicine and Pharmacy Timisoara, Eftimie Murgu Square No. 2, 300041 Timisoara, Romania; alex-robert.jijie@umft.ro; 8 Research Center for Pharmaco-Toxicological Evaluations, Faculty of Pharmacy, “Victor Babes” University of Medicine and Pharmacy Timișoara, Eftimie Murgu Square No. 2, 300041 Timișoara, Romania

**Keywords:** quinazoline, thiosemicarbazone, VEGFR2, structure design, synthesis, anti-angiogenic, anticancer, drug discovery, small molecules, tyrosine kinase inhibitors

## Abstract

**Background/Objectives:** Angiogenesis plays a crucial role in tumor development and is a driving force for the aggressiveness of several types of cancer. Our team developed a novel series of thiosemicarbazone-containing quinazoline derivatives, **TSC1-TSC10**, as potential VEGFR2 inhibitors with proven anti-angiogenic and antiproliferative potential. **Methods**: The **TSC1-TSC10** series was synthesized and characterized by spectral data. Extensive methodology was applied both in vitro (Alamar Blue assay, Scratch assay, CAM assay, and VEGFR2 kinase assay) and in silico (docking studies, MDs, and MM-PBSA) for the confirmation of the biological potential. **Results**: **TSC10** emerged as the most promising compound, with a favorable cytotoxic potential across the cell panel (Ea.Hy296, HaCaT, and A375) in agreement with the in vitro VEGFR2 kinase assay (IC_50_ = 119 nM). A comparable motility reduction in the vascular endothelial cells to that of the reference drug **sorafenib** was provided by **TSC10**, with a similar anti-angiogenic potential in the more complex in ovo model of the CAM assay. The in silico experiments confirmed the successful accommodation of the active site of the kinase domain similar to **sorafenib** for the entire **TSC1-TSC10** series, providing valuable key insight into the complex stability driving force for the evaluated compounds. **Conclusions**: The in vitro evaluations of the biological potential correlated with the in silico predictions by computer-aided complex simulations provided a solid confirmation of the initial hypothesis for the **TSC1-TSC10** series.

## 1. Introduction

Angiogenesis is a notorious hallmark of cancer that is vital for the development of solid tumors by ensuring the metabolic needs that are necessary for tumor expansion and metastasis [1,2]. The hypoxic intratumoral condition is the driver of neoangiogenesis, leading to the development of novel blood vessels through sprouting, splitting, vascular co-optation, or vasculogenic mimicry [3]. Angiogenesis is the result of a severely impaired vascular homeostasis that is associated with the upregulation of the pro-angiogenic factors that trigger the accelerated proliferation of the dormant endothelial cells and the formation of complex tumor blood vessel networks with severely impacted morphology [4,5]. Pro-angiogenic factors such as vascular endothelial growth factor (VEGF), platelet-derived growth factor (PDGF), fibroblast growth factor 2 (FGF-2), chemokines, and angiopoietins are actively and simultaneously released in the tumor microenvironment and mediate the rapid expansion of the endothelial cells [2,4]. VEGF is one of the most notorious pro-angiogenic factors that lead to the activation of several tyrosine-kinase receptors including the vascular endothelial growth factor receptor (VEGFR) family. This family consists of three members (VEGFR1, VEGFR2, and VEGFR3) that play a major role in angiogenesis and lymphangiogenesis, with particular implications in pathological and physiological processes [6].

**Figure 1:**
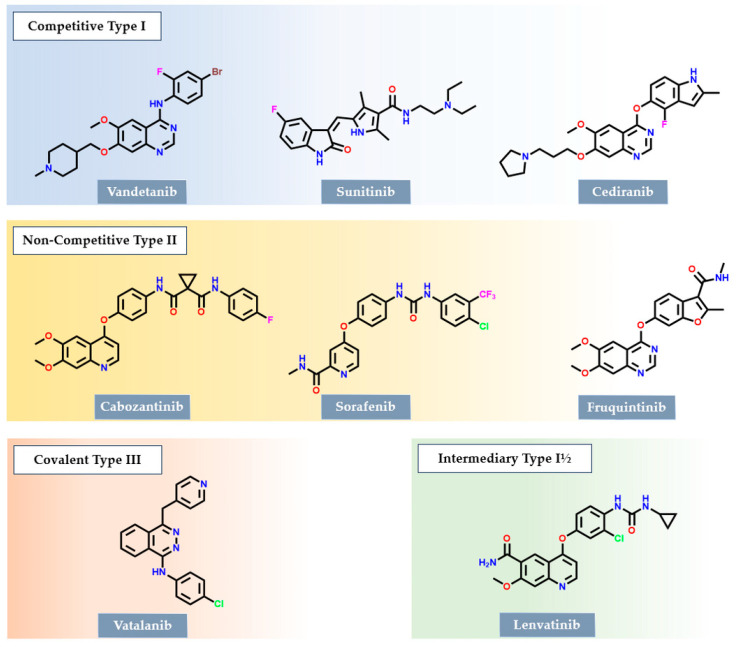
Chemical structures of clinical and preclinical VEGFR2 TKIs and their classification based on the general inhibitory mechanism.

VEGFR2 signaling is one of the main drivers of tumor angiogenesis and is critical for all the key steps of neovascularization [2,7]. VEGFR2 activation is directly linked to vascular endothelial cell proliferation through the activation of several intracellular phosphorylation cascades (phospholipase C pathway—PLCγ, inositol triphosphate—IP_3_, and RAS-MEK-ERK) [8]. VEGFR2 is essential for endothelial cell survival (PI3K/AKT/mTOR pathway), can facilitate endothelial cell motility (focal adhesion kinase pathway), and leads to enhancement of vascular permeability by increasing nitric oxide production [7,9,10,11]. VEGFR2 overexpression and aberrant signaling have been linked to several types of cancer, such as melanoma, hepatocellular carcinoma, colorectal cancer, thyroid cancer, and renal cell carcinoma, associated with limited prognosis and tumor aggressiveness. Its major role as a driving oncogene in cancer made VEGFR2 one of the most studied therapeutic targets for the development of novel anti-angiogenetic agents. To date, several VEGFR2 inhibitors have been developed and used in clinical practices for the treatment of multiple types of cancer [12]. One of the most widely spread techniques is the development of small molecules that inhibit the catalytic activity of the kinase domain (tyrosine kinase inhibitors—TKIs) leading to successful disruption of the signal transduction mediated by VEGFR2 [13]. Several examples of such VEGFR2 TKIs are provided in Figure 1.

The documented architecture of the catalytic domain of VEGFR2 plays a major role in the development of novel TKIs [13]. This domain is a narrow cleft, placed between the asymmetrical C and N-lobes, and consists of a front cleft and a back cleft, respectively. The front cleft is the main region that accommodates ATP, including the regions that bind adenine, pentose, and phosphate groups, while the back cleft is an extra binding site with access restricted by the gatekeeper Val916 [14,15]. Based on the interaction mechanism of the VEGFR2 TKIs with the catalytic domain, four different kinds of inhibitors can be distinguished: competitive type I (sunitinib, vandetanib, and cediranib), non-competitive type II (sorafenib, cabozantinib, and fruquintinib), covalent type III (vatalanib), and intermediary type I½ (lenvatinib) (Figure 1) [12,15]. Although considered a breakthrough for cancer treatment, current VEGFR2 therapy is facing major drawbacks in terms of efficiency, as well as the rapid development of resistance through secondary mutation, upregulation of angiogenic pathways, hypoxia-driven metastasis, vascular co-optation, mimicry, or the involvement of cancer stem cells [16,17,18]. Most of the approved VEGFR2 TKIs act as multi-target agents that lead to severe off-target effects and undesirable side effects [19].

This study aims to provide a novel molecular framework for the development of a series of VEGFR2 inhibitors as potential antiproliferative and anti-angiogenic agents, considering the structural model of already established and widely used VEGFR2 inhibitors.

## 2. Structural Design and Rationale

Existing crystallography data of several TKI-VEGFR2 complexes suggest the subdivision of the catalytic domain, from a pharmacophore perspective, into three major regions: two hydrophobic pockets (HYD I and HYD II) and a linker region, namely the DFG region. The HYD I region (the hinge region) is the main area occupied by ATP and is formed by amino acids (AAs) such as Leu840, Glu917, Phe918, and Cys919 in the proximity of the solvent-accessible area (Extra I). The gatekeeper area (Lys868, Val916) is a highly lipophilic region that requires a suitable molecular geometry for accommodation and restricts access to the DFG region [14,20,21,22,23]. Type I inhibitors are restricted to the accommodation of these three regions (HYD I, Extra I, gatekeeper), forming a series of hydrogen bonds (H-bonds) like ATP (Cys919, Glu918) with low kinase selectivity [11,15]. The DFG region is a narrow space and contains the hydrogen donor–acceptor pair Glu885-Asp1046, which generates key polar contacts with Mg^2+^ and phosphate groups facilitating the γ-phosphorylation [14,23]. The access to the HYD II (allosteric region) is further facilitated by the conformation of the DFG motif. An outward orientation to the phosphate region of the Phe1047 residue allows access to the highly lipophilic pocket HYD II that provides additional selectivity for type II inhibitors due to the conserved character of the surrounding AAs and the additional hydrophobic interactions [24,25].

**Figure 2:**
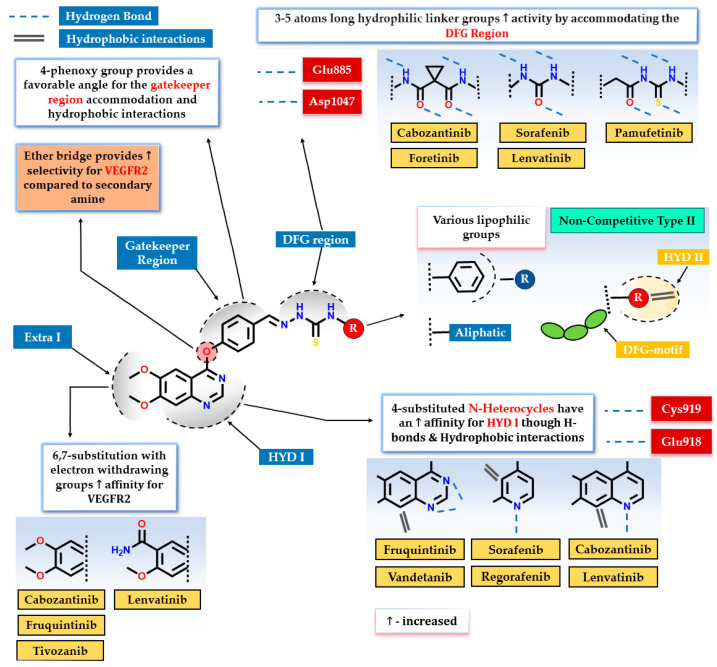
Structural design principles applied for the development of the **TSC1-TSC10** series.

The major structural features of type II inhibitors are correlated with the four main areas occupied (HYD I, gatekeeper, DFG region, and HYD II). A flat nitrogen-based heteroaromatic ring in the hinge region is mandatory for key H-bond interactions with Glu918 and Cys919 and stable hydrophobic interaction within the adenine binding region. A suitable aromatic core found within a favorable angle range with the main aromatic ring in the hinge region is vital for the accommodation in the gatekeeper area. Successful accommodation in the DFG region is achieved by a series of medium-length (3–5 atoms) hydrophilic linkers (urea, amide, and hydrazone) that form both donor–acceptor H-bonds with the pair Glu885-Asp1046. Finally, a hydrophobic moiety (large aromatic groups) at the distal end of the linker is mandatory for the occupation of the allosteric pocket in the DFG-out configuration, stabilizing the complex through hydrophobic interactions (Figure 2) [11,15,20,21,22,23].

Encouraged by the already existing solid structure–activity relationship (SAR) data and previously reported series, we aimed to develop a novel quinazoline series (**TSC1-TSC10**) as potential VEGFR2 inhibitors by applying the scaffold hopping approach technique (Figure 2) [15,23,26]. Due to the previous favorable results and established pharmacophore properties, the quinazoline core was chosen to accommodate the hinge region [13,26]. To maximize the electronic density on the quinazoline core with a minimal impact on molecular weight (M_w_), a 6,7-dimethoxy substitution that potentially extends in the Extra I area was chosen. A 4-phenoxy substitution on the quinazoline ring was selected to maximize the affinity towards VEGFR2, which could provide a suitable angle favorable for the accommodation of the gatekeeper area with the formation of stabilizing hydrophobic interactions. A 5-atom-long hydrophilic linker (thiosemicarbazone) was chosen to accommodate the DFG region due to its proven capability to form donor–acceptor H-bonds through the extended conjugated system [27]. Finally, various lipophilic groups were attached to the distal end of the thiosemicarbazone to accommodate the allosteric binding pocket and to stabilize the complex through hydrophobic interactions. Selected medium-size lipophilic groups (allyl and isopropyl) were chosen to evaluate the impact of smaller-size groups on biological activity.

## 3. Materials and Methods

### 3.1. Materials and Measurements

All the chemicals and solvents used in the synthetic protocols and biological assays were acquired from commercial local suppliers and used with no further purification. The reaction progression was monitored by thin layer chromatography (TLC) under UV light visualization using a mix of heptane: ethylacetate (3:7) as the mobile phase and silica gel-coated TLC Merck F254 plates as the stationary phase. The uncorrected melting points (°C) were recorded using an MPM-H1 device (Schorpp Gerätetechnik, Überlingen, Germany). An FT/IR 6100 spectrometer (Jasco, Cremella, Italy) was used to record the Fourier transform infrared (FT-IR) spectra by the KBr pellets technique, and the reported absorption peaks were represented in cm^−1^. Nuclear magnetic resonance (NMR) spectra were performed on an Avance NMR spectrometer (Bruker, Bremen, Germany) at 500 MHz for ^1^H-RMN and 125 MHz for ^13^C-RMN, respectively, using deuterated dimethylsulfoxide-*d*_6_ (DMSO-*d*_6_) as a solvent and tetramethyl silane (TMS) for calibration. The chemical shifts of ^1^H-RMN and ^13^C-RMN spectra were assessed using the DMSO-*d*_6_ peak as a reference and expressed in δ scale (ppm) with the coupling constants (*J*) in Hz, respectively. The designated multiplicity for the identified chemical shifts was abbreviated as follows: singlet (s), doublet (d), doublet of doublet (dd), triplet (t), doublet of quartet (dq), multiplet (m), and broad (br). The 3 aromatic moieties of the novel compounds were abbreviated as quinazoline (Q), the proximal phenyl ring directly bonded to the quinazoline ring through an ether bridge (Ar^1^), and the distal phenyl moiety (Ar^2^). After preliminary purity confirmation of the compounds by TLC, their purity was confirmed by RP-HPLC using an Agilent 1100 series device with UV detection (Agilent Technologies, Santa Clara, CA, USA), which was used to record the mass spectra (MS) reported in *m/z* (relative intensity) in positive ionization mode.

All the cell lines used in this study (Ea.Hy926, HaCaT, and A375) were acquired from ATCC (Manassas, VA, USA) and stored according to the manufacturer’s instructions. All culture reagents were acquired from Gibco (Pasley, UK): fetal bovine serum (FBS), penicillin/streptomycin (10.000 U/mL), Dulbecco’s modified Eagle’s medium (DMEM) with high or low glucose, phosphate-buffered saline solution (PBS), pyruvate (100 nM), and trypsin-EDTA.

### 3.2. Chemistry

#### 3.2.1. Synthesis of the Intermediate (**1**) and Thiosemicarbazide Derivatives **1a–10a**

The intermediate (**1**) and thiosemicarbazide derivatives ***1a–10a*** were prepared according to previously reported protocols with satisfying yields and corresponding spectral data [26,28,29,30,31,32,33]. Thiosemicarbazide derivatives were named as follows: *4-allyl-thiosemicarbazide* (**1a**)*; 4-(3-Trifluoromethyl-phenyl)-3-thiosemicarbazide* (**2a**)*; 4-(4-Fluoro-phenyl)-3-thiosemicarbazide* (**3a**)*; 4-(4-Chloro-phenyl)-3-thiosemicarbazide* (**4a**)*; 4-Phenyl-3-thiosemicarbazide* (**5a**); *4-(4-Chloro-3-trifluoromethyl-phenyl)-3-thiosemicarbazide* (**6a**)*; 4-(3,4-Difluoro-phenyl)-3-thiosemicarbazide* (**7a**); *4-(3-Methoxy-phenyl)-3-thiosemicarbazide* (**8a**)*; 4-Isopropyl-thiosemicarbazide* (**9a**)*;* and *4-(2,3-Dichloro-phenyl)-3-thiosemicarbazide* (**10a**).

#### 3.2.2. Synthesis of Compounds TSC1-TSC10

In a round bottom flask, 7 mL of ethanol (EtOH) was added to 310 mg of the intermediate (**1**) (1 mmol) and refluxed for 1 h under stirring conditions until complete dissolution. To the reaction mixture, 1 mmol of the corresponding thiosemicarbazide derivatives ***2a–8a, 10a*** or 1.25 mmol of ***1a***, ***9a***, respectively, was added. One drop of concentrated acetic acid was added as a catalyst and the stirring continued under reflux conditions for 5 h until the quantitative formation of a white to pale yellow precipitate as indicated by TLC. The formed precipitate was filtered off from the hot suspension, dried under a vacuum, rinsed repeatedly with EtOH, and crystallized using hot EtOH to afford the corresponding compounds **TSC1-TSC10**.

*N-allyl-2-(4-((6,7-dimethoxyquinazolin-4-yl)oxy)benzylidene)hydrazine-1-carbothioamide *(**TSC1**) white powder, mp = 250 °C; yield = 62.4%; **FT-IR** (KBr) ν_max_ cm^−1^: 1210.11 (C-O str.), 1377.41 (C-H bnd.), 1507.58 (C=C str.), 1616.58 (C=N str.), 3154.01 (N-H str.), 3352.64 (N-H str.); **^1^H-NMR** (DMSO-*d*_6_, 500 MHz) *δ* ppm: 3.982 (s, 3H, Q_6_-OCH_3_), 3.997 (s, 3H, Q_7_-OCH_3_), 4.242 (t, 2H, -CH_2_-, *J* = 8 Hz), 5.109 (dq, 1H, =CH_2_, *J*_1_ = 1.5 Hz, *J*_2_ = 10.5 Hz), 5.175 (dq, 1H, =CH_2_, *J*_1_ = 2 Hz, *J*_2_ = 17 Hz), 5.895–5.972 (m, 1H, -CH=), 7.378–7.393 (m, 3H–2H Ar^1^-H_3_, 1H Q-H_8_), 7.565 (s, 1H, Q-H_5_), 7.946 (d, 2H, Ar^1^-H_2_, *J* = 8.5 Hz), 8.127 (s, 1H, -CH=N-), 8.566 (s, 1H, Q-H_2_), 8.748 (t, 1H, -NH-, *J* = 6 Hz),11.567 (br. S, 1H, =N-NH-).**^13^C-NMR** (DMSO-*d*_6_, 125 MHz) *δ* ppm: 45.7 (=CH_2_), 55.9 (Q_7_-OCH_3_), 56.1 (Q_6_-OCH_3_), 100.6 (Q_8_), 106.7 (Q_4a_), 109.7 (Q_5_), 115.4 (-CH_2_-) 122.4 (Ar^1^-C_3_), 128.5 (Ar^1^-C_2_), 131.6 (Ar^1^-C_1_), 135.0 (-CH=) 141.1 (-CH=N-), 148.9 (Q_8a_), 150.0 (Q_6_), 152.1 (Q_7_), 153.5 (Ar^1^-C_4_), 155.7 (Q_4_), 164.6 (Q_2_), 177.2 (C=S); and **MS**: *m/z* = 424.4 [M + H]^+^, calcd 423.14.

*N-(3-(trifluoromethyl)-phenyl)-2-(4-((6,7-dimethoxyquinazolin-4-yl)oxy)benzylidene)-hyrazine-1-carbothioamide* (**TSC2**) pale-yellow powder, mp = 213 °C; yield = 75%; **FT-IR** (KBr) ν_max_ cm^−1^: 1164.31 (C-F str.), 1212.04 (C-O str.), 1375.48 (C-H bnd.), 1503.24 (C=C str.), 1618.47 (C=N str.), 3141.47 (N-H str.), 3284.18 (N-H str.); **^1^H-NMR** (DMSO-*d*_6_, 500 MHz) *δ* ppm: 3.987 (s, 3H, Q_6_-OCH_3_), 4.001 (s, 3H, Q_7_-OCH_3_), 7.403–7.429 (m, 3H–2H Ar^1^-H_3_, 1H Q-H_8_), 7.554–7.578 (m, 2H, 1H–Ar^2^-H_4_; 1H Q-H_5_), 7.619 (t, 1H, Ar^2^-H_5_, *J* = 8 Hz), 7.841 (d, 1H, Ar^2^-H_6_, *J* = 8.5 Hz), 8.042–8.068 (m, 3H, 2H–Ar^1^-H_2_, *J* = 8.5 Hz, s, 1H, Ar^2^-H_2_), 8.250 (s, 1H, -CH=N-), 8.577 (s, 1H, Q-H_2_), 10.346 (br. s, 1H, -NH-),12.057 (br. s, 1H, =N-NH-); **^13^C-NMR** (DMSO-*d*_6_, 125 MHz) *δ* ppm: 56.0 (Q_7_-OCH_3_), 56.1 (Q_6_-OCH_3_), 100.6 (Q_8_), 106.7 (Q_4a_), 109.7 (Q_5_), 121.5 (Ar^2^-C_2_, *J* = 3.5 Hz), 121.9 (Ar^2^-C_4_, *J* = 3.5 Hz), 121.9 (CF_3_, *J* = 280 Hz), 122.4 (Ar^1^-C_5_), 128.7 (Ar^2^-C_3_, *J* = 31.5 Hz), 129.0 (Ar^2^-C_5_, *J* = 2.6 Hz), 129.0 (Ar^1^-C_2_), 129.5 (Ar^2^-C_6_), 131.6 (Ar^1^-C_1_), 139.8 (Ar^2^-C_1_) 142.7 (-CH=N-), 148.9 (Q_8a_), 150.1 (Q_6_), 152.1 (Q_7_), 153.8 (Ar^1^-C_4_), 155.8 (Q_4_), 164.6 (Q_2_), 175.9 (C=S); and **MS**: *m/z* = 528.4 [M + H]^+^, calcd = 527.12.

*N-(4-fluorophenyl)-2-(4-((6,7-dimethoxyquinazolin-4-yl)oxy)benzylidene)hydrazine-1-carbothioamide* (**TSC3**) pale-yellow powder, mp = 233–234 °C; yield = 95%; **FT-IR** (KBr) ν_max_ cm^−1^: 1166.24 (C-F str.), 1212.04 (C-O str.), 1375.96 (C-H bnd.), 1510.95 (C=C str.), 1620.88 (C=N str.), 3152.08 (N-H str.), 3328.05 (N-H str.); **^1^H-NMR** (DMSO-*d*_6_, 500 MHz) *δ* ppm: 3.982 (s, 3H, Q_6_-OCH_3_), 3.996 (s, 3H, Q_7_-OCH_3_), 7.125 (t, 2H, Ar^2^-H_3_, *J* = 9 Hz), 7.391–7.411 (m, 3H–2H Ar^1^-H_3_; 1H Q-H_8_), 7.551–7.579 (m, 3H, 2H Ar^2^-H_2_, 1H, Q-H_5_), 8.043 (d, 2H, Ar^1^-H_2_, *J* = 8.5 Hz), 8.220 (s, 1H, -CH=N-), 8.572 (s, 1H, Q-H_2_), 10.168 (br. s, 1H, -NH-),11.897 (br. s, 1H, =N-NH-).**^13^C-NMR** (DMSO-*d*_6_, 125 MHz) *δ* ppm: 55.9 (Q_7_-OCH_3_), 56.1 (Q_6_-OCH_3_), 100.6 (Q_8_), 106.7 (Q_4a_), 109.7 (Q_5_), 114.6 (Ar^2^-C_3_, *J* = 21.8 Hz), 122.4 (Ar^1^-C_3_), 128.1 (Ar^2^-C_2_, *J* = 7.8 Hz), 128.9 (Ar^1^-C_2_), 131.4 (Ar^1^-C_1_), 135.4 (Ar^2^-C_1_, *J* = 2.6 Hz), 142.1 (-CH=N-), 148.9 (Q_8a_), 150.1 (Q_6_), 152.1 (Q_7_), 153.7 (Ar^1^-C_4_), 155.7 (Q_4_), 159.6 (Ar^2^-C_4_, *J* = 240.5 Hz), 164.5 (Q_2_), 176.3 (C=S); and **MS**: *m/z* = 478.3 [M + H]^+^, calcd = 477.13.

*N-(4-chlorophenyl)-2-(4-((6,7-dimethoxyquinazolin-4-yl)oxy)benzylidene)hydrazine-1-carbothioamide* (**TSC4**) pale-yellow powder, mp = 240–241 °C; yield = 82.6%; FT **IR** (KBr) ν_max_ cm^−1^: 768.98 (C-Cl str.), 1211.08 (C-O str.), 1374.51 (C-H bnd.), 1500.83 (C=C str.), 1619.91 (C=N str.), 3148.92 (N-H str.), 3320.34 (N-H str.); **^1^H-NMR** (DMSO-*d*_6_, 500 MHz) *δ* ppm: 3.986 (s, 3H, Q_6_-OCH_3_), 4.001 (s, 3H, Q_7_-OCH_3_), 7.399–7.445 (m, 5H–2H Ar^2^-H_2_, 2H Ar^1^-H_3_, 1H Q-H_8_), 7.577 (s, 1H, Q-H_5_), 7.635 (d, 2H, Ar^2^-H_3_, *J* = 9 Hz), 8.045 (d, 2H, Ar^2^-H_3_, *J* = 9 Hz), 8.227 (s, 1H, -CH=N-), 8.575 (s, 1H, Q-H_2_), 10.199 (br. s, 1H, -NH-),11.948 (br. s, 1H, =N-NH-); **^13^C-NMR** (DMSO-*d*_6_, 125 MHz) *δ* ppm: 56.0 (Q_7_-OCH_3_), 56.1 (Q_6_-OCH_3_), 100.6 (Q_8_), 106.7 (Q_4a_), 109.7 (Q_5_), 122.4 (Ar^1^-C_3_), 127.5 (Ar^2^-C_2_), 127.9 (Ar^1^-C_2_), 129.0 (Ar^2^-C_3_), 129.2 (Ar^2^-C_4_), 131.4 (Ar^1^-C_1_), 138.0 (Ar^2^-C_1_), 142.3 (-CH=N-), 148.9 (Q_8a_), 150.1 (Q_6_), 152.1 (Q_7_), 153.7 (Ar^1^-C_4_), 155.8 (Q_4_), 164.6 (Q_2_), 175.9 (C=S); and **MS**: *m/z* = 494.2 [M + H]^+^, calcd = 493.10.

*N-phenyl-2-(4-((6,7-dimethoxyquinazolin-4-yl)oxy)benzylidene)hydrazine-1-carbothioamide* (**TSC5**) white powder, mp = 245 °C; yield = 95%; **FT-IR** (KBr) ν_max_ cm^−1^: 1210.06 (C-O str.), 1373.55 (C-H bnd.), 1502.76 (C=C str.), 1619.43 (C=N str.), 3163.65 (N-H str.), 3295.27 (N-H str.); **^1^H-NMR** (DMSO-*d*_6_, 500 MHz) *δ* ppm: 3.985 (s, 3H, Q_6_-OCH_3_), 4.000 (s, 3H, Q_7_-OCH_3_), 7.203–7.238 (m, 1H, Ar^2^-H_4_), 7.369–7.415 (m, 5H–2H Ar^2^-H_3_, 2H Ar^1^-H_3_, 1H Q-H_8_), 7.574–7.595 (m, 3H–2H Ar^2^-H_2_, 1H, Q-H_5_), 8.047 (d, 2H, Ar^1^-H_2_, *J* = 8.5 Hz), 8.224 (s, 1H, -CH=N-), 8.575 (s, 1H, Q-H_2_), 10.164 (br. s, 1H, -NH-),11.867 (br. s, 1H, =N-NH-); **^13^C-NMR** (DMSO-*d*_6_, 125 MHz) *δ* ppm: 56.0 (Q_7_-OCH_3_), 56.1 (Q_6_-OCH_3_), 100.6 (Q_8_), 106.7 (Q_4a_), 109.7 (Q_5_), 122.4 (Ar^1^-C_3_), 125.3 (Ar^2^-C_4_), 125.9 (Ar^2^-C_2_), 128.0 (Ar^1^-C_2_), 128.9 (Ar^2^-C_3_), 131.5 (Ar^1^-C_1_), 139.0 (Ar^2^-C_1_), 142.0 (-CH=N-), 148.9 (Q_8a_), 150.1 (Q_6_), 152.1 (Q_7_), 153.7 (Ar^1^-C_4_), 155.7 (Q_4_), 164.6 (Q_2_), 175.9 (C=S); and **MS**: *m/z* = 460.3 [M + H]^+^, calcd = 459.14.

*N-(4-chloro-3-(trifluoromethyl)phenyl)-2-(4-((6,7-dimethoxyquinazolin-4-yl)oxy)benzylidene)hydrazine-1-carbothioamide* (**TSC6**); pale-yellow powder, mp = 241 °C; yield = 84.3%; **FT-IR** (KBr) ν_max_ cm^−1^: 1198.54 (C-F str.), 1379.82 (C-H bnd.), 1502.76 (C=C str.), 1614.61 (C=N str.), 3122.19(N-H str.), 3299.12 (N-H str.); **^1^H-NMR** (DMSO-*d*_6_, 500 MHz) *δ* ppm: 3.987 (s, 3H, Q_6_-OCH_3_), 4.002 (s, 3H, Q_7_-OCH_3_), 7.403–7.436 (m, 3H, 2H Ar^1^-H_3_, 1H Q-H_8_), 7.577 (s, 1H, Q-H_5_), 7.733 (d, 1H, Ar^2^-H_6_, *J* = 8.5 Hz), 8.020–8.062 (m, 3H, 2H Ar^1^-H_2_, 1H Ar^2^-H_5_), 8.211 (d, 2H, -CH=N-, *J* = 2 Hz), 8.256 (s, 1H, Ar^2^-H_2_), 8.576 (s, 1H, Q-H_2_), 10.373 (br. s, 1H, -NH-),12.136 (br. s, 1H, =N-NH-),**^13^C-NMR** (DMSO-*d*_6_, 125 MHz) *δ* ppm: 56.0 (Q_7_-OCH_3_), 56.1 (Q_6_-OCH_3_), 100.6 (Q_8_), 106.7 (Q_4a_), 109.7 (Q_5_), 122.5 (Ar^1^-C_3_), 122.7 (Ar^2^-C_2_, *J* = 271 Hz), 124.3 (CF_3_, *J* = 5.25 Hz), 126.0 (Ar^2^-C_3_, *J* = 16.5 Hz), 126.2 (Ar^2^-C_4_, *J* = 1.75 Hz), 129.1 (Ar^1^-C_2_), 130.6 (Ar^2^-C_5_), 131.1 (Ar^2^-C_6_), 131.2 (Ar^1^-C_1_), 138.5 (Ar^2^-C_1_), 143.0 (-CH=N-), 148.9 (Q_8a_), 150.1 (Q_6_), 152.1 (Q_7_), 153.6 (Ar^1^-C_4_), 155.8 (Q_4_), 164.5 (Q_2_), 175.8 (C=S); and **MS**: *m/z* = 562.4 [M + H]^+^, calcd = 561.08.

*N-(3,4-difluorophenyl)-2-(4-((6,7-dimethoxyquinazolin-4-yl)oxy)benzylidene)hydrazine-1-carbothioamide* (**TSC7**)); pale-yellow powder, mp = 229 °C; yield = 52%%; **FT-IR** (KBr) ν_max_ cm^−1^: 1155.63 (C-F str.), 1211.56 (C-O str.) 1379.34 (C-H bnd.), 1502.76 (C=C str.), 1614.61 (C=N str.), 3155.64 (N-H str.), 3330.94 (N-H str.); **^1^H-NMR** (DMSO-*d*_6_, 500 MHz) *δ* ppm: 3.987 (s, 3H, Q_6_-OCH_3_), 4.002 (s, 3H, Q_7_-OCH_3_), 7.405–7.480 (m, 5H–1H Ar^2^-H_2_, 1H Ar^2^-H_6_, 2H Ar^1^-H_3_, 1H Q-H_8_), 7.578 (s, 1H, Q-H_5_), 7.760–7.802 (m, 1H, Ar^2^-H_5_), 8.045 (d, 2H, Ar^1^-H_2_, *J* = 8.5 Hz), 8.230 (s, 1H, -CH=N-), 8.576 (s, 1H, Q-H_2_), 10.217 (br. s, 1H, -NH-),12.005 (br. s, 1H, =N-NH-), **^13^C-NMR** (DMSO-*d*_6_, 125 MHz) *δ* ppm: 56.0 (Q_7_-OCH_3_), 56.1 (Q_6_-OCH_3_), 100.6 (Q_8_), 106.7 (Q_4a_), 109.7 (Q_5_), 115.1 (Ar^2^-C_5_, *J* = 20 Hz), 116.4 (Ar^2^-C_2_, *J* = 18.3 Hz), 122.4 (Ar^1^-C_3_), 122.5 (Ar^2^-C_6_, *J* = 2.6 Hz), 129.0 (Ar^1^-C_2_), 131.3 (Ar^1^-C_1_), 136.0 (Ar^2^-C_1_, *J*_1_ = 3.5 Hz, *J*_2_ = 8.75 Hz), 142.5 (-CH=N-), 146.9 (Ar^2^-C_4_, *J*_1_ = 12.25 Hz, *J*_2_ = 242 Hz), 148.9 (Q_8a_), 149.2 (Q_6_), 151.1 (Ar^2^-C_3_, *J*_1_ = 17.5 Hz, *J*_2_ = 242 Hz) 152.1 (Q_7_), 153.8 (Ar^1^-C_4_), 155.8 (Q_4_), 164.5 (Q_2_), 176.0 (C=S); and **MS**: *m/z* = 496.4 [M + H]^+^, calcd = 495.12.

*N-(3-methoxyphenyl)-2-(4-((6,7-dimethoxyquinazolin-4-yl)oxy)benzylidene)hydrazine-1-carbothioamide* (**TSC8**); white powder, mp = 225 °C; yield = 83.2%; **FT-IR** (KBr) ν_max_ cm^−1^: 1214.93 (C-O str.) 1378.37 (C-H bnd.), 1503.24 (C=C str.), 1618.47 (C=N str.), 3148.22 (N-H str.), 3340.10 (N-H str.); **^1^H-NMR** (DMSO-*d*_6_, 500 MHz) *δ* ppm: 3.777 (s, 3H, Ar^2^-OCH_3_), 3.985 (s, 3H, Q_6_-OCH_3_), 4.000 (s, 3H, Q_7_-OCH_3_), 6.783–6.806 (m, 1H, Ar^2^-H_4_), 7.192–7.210 (m, 1H, Ar^2^-H_2_), 7.264–7.300 (m, 2H, Ar^2^-H_5,_ Ar^2^-H_6_), 7.389–7.412 (m, 3H, 2H Ar^1^-H_3_, 1H Q-H_8_), 7.576 (s, 1H, Q-H_5_), 8.043 (d, 2H, Ar^1^-H_2_, *J* = 9 Hz), 8.223 (s, 1H, -CH=N-), 8.574 (s, 1H, Q-H_2_), 10.109 (br. s, 1H, -NH-), 11.875 (br. s, 1H, =N-NH-); **^13^C-NMR** (DMSO-*d*_6_, 125 MHz) *δ* ppm: 55.1 (Ar^2^-OCH_3_) 56.0 (Q_7_-OCH_3_), 56.1 (Q_6_-OCH_3_), 100.6 (Q_8_), 106.7 (Q_4a_), 109.7 (Q_5_), 110.7 (Ar^2^-C_5_), 111.3 (Ar^2^-C_2_), 117.8 (Ar^2^-C_6_), 122.4 (Ar^1^-C_3_), 128.7 (Ar^1^-C_2_), 122.9 (Ar^2^-C_4_), 131.4 (Ar^1^-C_1_), 140.1 (Ar^2^-C_1_), 142.0 (-CH=N-), 148.9 (Q_8a_), 150.1 (Q_6_), 152.1 (Q_7_), 153.7 (Ar^1^-C_4_), 155.8 (Q_4_), 158.9 (Ar^2^-C_3_), 164.6 (Q_2_), 175.7 (C=S); and **MS**: *m/z* = 490.4 [M + H]^+^, calcd = 489.15.

*N-isopropyl-2-(4-((6,7-dimethoxyquinazolin-4-yl)oxy)benzylidene)hydrazine-1-carbothioamide* (**TSC9**); white powder, mp = 249–250 °C; yield = 55.5%; **FT-IR** (KBr) ν_max_ cm^−1^: 1207.7 (C-O str.), 1375 (C-H bnd.), 1498.42 (C=C str.), 1620.88 (C=N str.), 3162.20 (N-H str.), 3351.68 (N-H str.); **^1^H-NMR** (DMSO-*d*_6_, 500 MHz) *δ* ppm: 1.236 (s, 3H, -CH_3_), 1.249 (s, 3H, -CH_3_), 3.987 (s, 3H, Q_6_-OCH_3_), 4.002 (s, 3H, Q_6_-OCH_3_), 4.527–4.597 (m, 1H, -CH-), 7.382–7.404 (m, 3H, 2H Ar^1^-H_3_, 1H Q-H_8_), 7.577 (s, 1H, Q-H_5_), 7.927 (d, 2H, Ar^1^-H_2_, *J* = 8.5 Hz), 8.125 (s, 1H, -CH=N-), 8.568 (s, 1H, Q-H_2_),11.441 (br. s, 1H, =N-NH-); **^13^C-NMR** (DMSO-*d*_6_, 125 MHz) *δ* ppm: 21.8 (-CH_3_), 45.5 (-CH-), 56.0 (Q_7_-OCH_3_), 56.1 (Q_6_-OCH_3_), 100.6 (Q_8_), 106.7 (Q_4a_), 109.7 (Q_5_), 122.4 (Ar^1^-C_3_), 128.6 (Ar^1^-C_2_), 131.5 (Ar^1^-C_1_), 141.2 (-CH=N-), 148.9 (Q_8a_), 150.1 (Q_6_), 152.1 (Q_7_), 153.5 (Ar^1^-C_4_), 155.8 (Q_4_), 164.6 (Q_2_), 175.7 (C=S); and **MS**: *m/z* = 426.2 [M + H]^+^, calcd = 425.15.

*(E)-N-(2,3-dichlorophenyl)-2-(4-((6,7-dimethoxyquinazolin-4-yl)oxy)benzylidene)hydrazine-1-carbothioamide* (**TSC10**); pale-yellow powder, mp = 210–211 °C; yield = 86.2%; **FT-IR** (KBr) ν_max_ cm^−1^: 721.25 (C-Cl str.), 1215.42 (C-O str.), 1378.85 (C-H bnd.), 1502.28 (C=C str.), 1619.43 (C=N str.), 3168.47 (N-H str.), 3263.45 (N-H str.); **^1^H-NMR** (DMSO-*d*_6_, 500 MHz) *δ* ppm: 3.984 (s, 3H, Q_6_-OCH_3_), 3.999 (s, 3H, Q_6_-OCH_3_), 7.400–7.436 (m, 4H–2H Ar^1^-H_3_, 1H Q-H_8_, 1H Ar^2^-H_4_), 7.573–7.607 (m, 2H, 1H Ar^2^-H_5_, 1H Q-H_5_), 7.642–7.660 (m, 1H Ar^2^-H_6_), 8.022 (d, 2H, Ar^1^-H_2_, *J* = 8.5 Hz), 8.229 (s, 1H, -CH=N-), 8.573 (s, 1H, Q-H_2_), 10.236 (br. s, 1H, -NH-), 12.105 (br. s, 1H, =N-NH-),**^13^C-NMR** (DMSO-*d*_6_, 125 MHz) *δ* ppm: 56.0 (Q_7_-OCH_3_), 56.1 (Q_6_-OCH_3_), 100.6 (Q_8_), 106.7 (Q_4a_), 109.7 (Q_5_), 122.5 (Ar^1^-C_3_), 127.5 (Ar^2^-C_6_), 128.3 (Ar^2^-C_4_), 128.9 (Ar^1^-C_2_), 129.0 (Ar^2^-C_5_), 129.9 (Ar^2^-C_2_), 131.3 (Ar^1^-C_1_), 131.5 (Ar^2^-C_3_), 138.7 (Ar^2^-C_1_), 142.3 (-CH=N-), 148.9 (Q_8a_), 150.1 (Q_6_), 152.1 (Q_7_), 153.8 (Ar^1^-C_4_), 155.8 (Q_4_), 164.6 (Q_2_), 176.7 (C=S); and **MS**: *m/z* = 528.1 [M + H]^+^, calcd = 527.06.

### 3.3. In Vitro Cytotoxicity Evaluation

The antiproliferative potential of the **TSC1-TSC10** series and the reference compound **sorafenib** was evaluated on a selected panel of cell lines that consist of Ea.Hy926 cells (human vascular endothelial hybrid cells, passage number 5–10), HaCaT cells (immortal keratinocyte cells, passage number 5–10), and A375 cells (human malignant melanoma cells, passage number 10–15). The Ea.Hy926 cells were cultivated in a DMEM growth media with a high glucose concentration (4.5%) and 1% pyruvate, while the HaCaT and A375 cells were cultivated in DMEM growth media with a high glucose concentration and a daily refresh of the media. To the growth media of all the cell lines, 1% penicillin/streptomycin and 10% FBS were added. The cell lines were cultured in standardized conditions (37 °C and 5% CO_2_) with the initiation of the experimental protocol once an 80–90% confluency was reached.

The cytotoxic potential of the **TSC1-TSC10** series and reference compound **sorafenib** was assessed using the Alamar Blue (AB) protocol as previously reported [26,34]. The spectrophotometric quantification (*λ_emission_ =* 590/35, *λ_excitation_ =* 530/25) of resorufin by the metabolic-dependent conversion of the resazurin by the viable cells was achieved using the microplate reader Synergy 2 Multi-Mode. The cells were seeded in 100 µL of growth media in 96-well plates at the specified density (Ea.Hy296—5 × 10^3^ cells/well, HaCaT—8 × 10^3^ cells /well, and A375—7 × 10^3^ cells /well) and were left overnight to attach. The attached cells were rinsed further with PBS and exposed to different ranges of concentrations (up-and-down method) of the **TSC1-TSC10** series and the reference drug **sorafenib** for the calculation of the 50% inhibitory concentration (IC_50_). After 48 h of cultivation, the growth media was removed, and the AB assay was performed for the quantification of the viable cells post-exposure to the selected compounds. Three biological replicates were performed for each biological experiment with six technical replicates, using 0.2% DMSO as the negative control (NC) for data normalization (100% viability). The IC_50_ values were calculated based on the dose–cellular viability curve ratio obtained by fitting the experimental data with a 4-parameter logistic curve (SigmaPlot 11).

### 3.4. In Vitro VEGFR2 Kinase Inhibition Assay

The inhibitory potential towards the VEGFR2 kinase domain of the **TSC1-TSC10** series and reference drug **sorafenib** was assessed using the Human VEGFR-2 (KDR) kinase assay kit (BPS Bioscience, San Diego, CA, USA, catalog #40325). The experimental assay was conducted according to the manufacturer’s instructions. An initial screening of the series was performed at the 500 nM concentration with further selection for the IC_50_ calculation of the compounds with a minimal 50% kinase inhibitory activity in the initial screening. Selected concentrations for the IC_50_ calculation were as follows: **sorafenib** (62.5 nM, 31.25 nM, 15.62 nM, 7.81 nM, and 3.90 nM) and for the selected **TSC series** (1000 nM, 500 nM, 250 nM, 125 nM, and 62.5 nM). The experimental procedure was performed in a 96-well microplate in the presence of Master Mix (ATP and PTK substrate in kinase buffer) and the isolated kinase domain of VEGFR2 (AAs 805–1356) using the kinase buffer provided by the kit as a solvent with an addition of 0.2% DMSO (in the final mix per well) for dissolution of the evaluated compounds. The incubation time was set for 45 min at 30 °C. Inhibitory potential towards the kinase domain was quantified by luminescence detection, after the addition of the Kinase-Glo™ MAX reagent (Promega, Madison, WI, USA, catalog #v6071). After further incubation for an additional 15 min, the luminescence was recorded using a microplate reader Synergy 2 Multi-Mode. The IC_50_ values were calculated based on the dose–kinase inhibition percent curve ratio obtained by fitting the experimental data with a 4-parameter logistic curve (SigmaPlot 11.0).

### 3.5. Wound Healing (Scratch) Assay

Confluent Ea.Hy926 monolayers were formed by seeding the cells for 24 h in a 12-well plate incubated in a starvation medium. After the confluency was reached, the medium was removed and a scratch was performed using the sterile tip of a 100 µL pipette. Detached cells were removed by washing the cellular monolayers twice with DPBS, and then the cells were photographed individually (phase-contrast microscopy, 10x objective) to assess the wound area at the moment of exposure (T_0_). Subsequently, the plates were exposed to the evaluated compounds: **sorafenib** (1.25 µM, 2.5 µM, and 5 µM), **TSC5** (1.25 µM, 2.5 µM, and 5 µM), **TSC9** (5 µM, 10 µM, and 12.5 µM), **TSC10** (2.5 µM, 5 µM, and 7.5 µM), and **0.2% DMSO** solution as the NC, dissolved in the media. For each experiment, an image was captured 12 h post-exposure (T_12_), and the rate of Ea.Hy926 cell migration was quantitatively evaluated by the wound healing percentage (%) calculated by the formula (A_T0_ − A_T12_)/ A_T0_ × 100 [35]. The scratch areas at T_0_ (A_T0_) and T_12_ (A_T12_), respectively, were determined with the IKOSA Prisma Application Wound Healing (Scratch) assay (v2.2.0) and are expressed as square pixels (Px^2^) [36].

### 3.6. Chorioallantoic Egg Membrane (CAM) Assay

To examine the impact of the selected compounds (**TSC5**, **TSC9**, **TSC10**, and **sorafenib**) on the angiogenic process, an experimental protocol that uses the chick chorioallantoic egg membrane (CAM) assay was employed. Eggs without deformities and weighing between 50 and 60 g were selected. The eggs were cleaned with 70% alcohol and incubated for 14 days in standard conditions (37.5 °C, humidity 65%). On the 4th day of incubation, approximately 10–12 mL of egg white (albumen) was extracted from the top of each egg using a medical syringe with a needle for the detachment of the egg membrane. On the 5th day, the eggs were checked for fertility using a light source, and a window large enough to reveal the vascular plexus was cut out with forceps. The window was then sealed with medical tape to maintain favorable conditions for embryonic development. Optimal ventilation conditions, temperature, and humidity were ensured throughout incubation to facilitate proper embryo growth. On the 10th day when the embryo and the CAM vascularization had an optimal development, the CAM assay was performed. For this purpose, a transparent silicone ring was applied to the vascular plexus of the CAM using a tweezer. In this ring, 10 μL of each test sample was applied for 4 days. The evaluated compounds, **TSC5**, **TSC9**, **TSC10,** and **sorafenib,** were assessed in a successive series of dilutions (1μM, 5 μM, and 10 μM) along with **0.2% DMSO** as the NC. Pictures were taken daily (days 1–5 of exposure) to observe the effects of the samples on the angiogenic process, and the changes observed were noted. For microscopic analysis, a Zeiss SteREO Discovery.V8 stereomicroscope was used, coupled with a Zeiss Axiocam 105 digital camera, both connected to a computer. The images presented in this study were captured using the ZEN core 3.8 software. The scale bar from the images represents a measurement of 500 μm.

### 3.7. ADMETox and Drug-Likeness Profile

Physicochemical descriptors and drug-likeness evaluations were predicted using the web tool SwissADME developed by the Swiss Institute of Bioinformatics (Lausanne, Switzerland), which is freely available [37]. ADME parameters (absorption, distribution, metabolism, and elimination) and toxicological potential were further predicted using the pkCSM web tool [38].

### 3.8. Molecular Docking Studies

The files with the three-dimensional structures of the compounds **TSC1-10** and **sorafenib** were obtained in the DFT calculations and processed using the previously reported protocol using AutoDock Tools 1.5.6 [22,26,39,40]. They were docked into the three-dimensional structure of human VEGFR2 obtained from homology modeling and reported in our previous paper using AutoDock Vina 1.1.2 [41]. The search parameters used were the same as reported by our group: coordinates of the center x = −21.966, y = −0.473, and z = −11.442; sides of the search space equal to 20; and exhaustiveness and num_modes were set to 50 and 20, respectively [26,41]. The visual inspection of the generated poses of the ligands into the binding site of the protein was performed using UCFS Chimera 1.10.2 [42]. The 2D depictions of the interactions between the studied ligands and protein were created using LigPlot + 2.2.8 [43].

### 3.9. Density Function Theory (DFT) Calculations

The in silico DFT calculations were performed using Spartan 20 (Wavefunction, Inc., Irvine, CA, USA) to identify the electronic and structural characteristics of the compounds **TSC1-10** and **sorafenib** as reference drugs. The calculations were performed as previously reported by our group to identify the regions of the molecules important for their interaction with VEGFR2 according to their electron density, angles, frontier molecular orbitals (FMOs), and dipole momentum. Based on Koopman’s theory, the chemical reactivity descriptors were also calculated by the following formulas: ionization potential (I = −E_HOMO_); electronic affinity (E = −E_LUMO_); chemical potential (µ = (I + E)/2)); chemical hardness (η = I − E); chemical softness (S = 1/2η); electrophilicity index (ω = µ^2^/2η); nucleophilicity index (N = 1/ω); and additional electronic charges (∆N = −µ/η). For a better insight into the charge distribution, electrostatic potential maps (EPMs) were generated for each compound [26,44,45].

### 3.10. Molecular Dynamics Studies

To gain a more comprehensive understanding of the interactions between the ligands and VEGFR2, the dynamic behavior of the predicted complexes was explored through molecular dynamics (MD) simulations using GROMACS 2023 under the CHARMM36 force field as previously reported [46,47,48,49,50]. The simulations ran for 100 ns on a Linux Debian 11 machine with CUDA 12 on a 12700KF CPU (Santa Clara, CA, USA) and an NVIDIA RTX 3060 GPU (Santa Clara, CA, USA). The most favorable binding conformation of each ligand in the complex with the protein was evaluated, and their parametrization was made using CgenFF [46]. The systems were solvated with the TIP3P water model inside an orthorhombic box with a 1 nm gap at each side, and subsequently neutralized. Energy minimization was performed via the steepest descent method with a convergence threshold of less than 1000 KJ mol^−1^ nm^−1^. System equilibration followed, using NVT and NPT ensembles at 300 K for 100 ps. The simulations were conducted with periodic boundary conditions on all axes, and temperature and pressure were regulated using a velocity rescaling thermostat and a Parrinello–Rahman barostat, respectively, with a reference temperature of 300 K and a reference pressure of 1 bar [51,52]. Visualization of the trajectories was carried out using VMD 1.9.4 [53], while the stability of the complexes was quantitatively analyzed using the integrated functionalities of both GROMACS and VMD 1.9.4.

### 3.11. MM-PBSA Free Energy Calculation

The average free binding energy of the ligands **TSC1-TSC10** and **sorafenib** to VEGFR2 was calculated using the Molecular Mechanics–Poisson–Boltzmann Surface Area (MM-PBSA) method, implemented through gmx_MMPBSA as previously reported by our group [26,54]. The analysis focused on the last 25 ns of each MD’s simulation, evaluating a total of 2500 frames. This assessment aimed to determine the energy contributions of each AA within a 5 Å radius using an energy decomposition approach. The binding energy difference between the ligand and the protein and the unbound ligand was calculated using the formula ΔG_binding_ = G_complex_ − (G_complex_ + G_ligand_) [55].

## 4. Results and Discussion

### 4.1. Chemistry

**Scheme 1:**
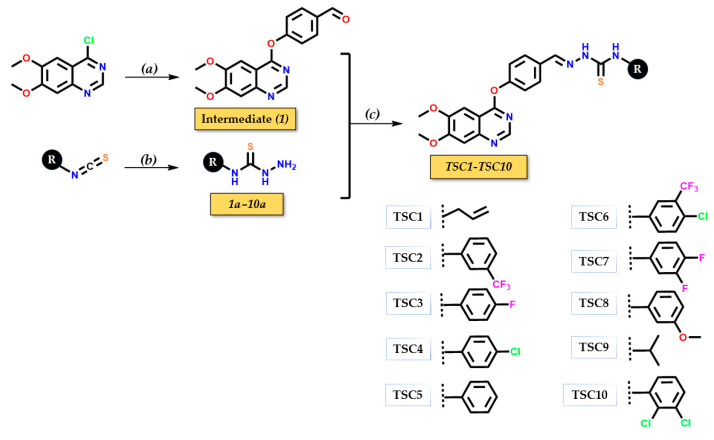
Synthetic procedure of the quinazoline derivatives **TSC1-TSC10**: (**a**) 4-hydroxy-benzaldehyde, K_2_CO_3_ (1:3), and 10 h reflux in MeCN; (**b**) isothiocyanates, hydrazine hydrate (80%, 1:5), EtOH, and 5 h reflux; and (**c**) 1 drop of conc. acetic acid, EtOH, and 5 h reflux.

Ten novel thiosemicarbazone-containing quinazoline derivatives (**TSC1**-**TSC10**) were successfully synthesized following the route depicted in Scheme 1. The aldehyde intermediate (1) was synthesized in high yield (89%) following a previously reported protocol and all spectral data are consistent with the already existing reports of similar compounds. Briefly, the intermediate (**1**) was synthesized through the alkylation of 4-hydroxy-benzaldehyde with 6,7-dimethoxy-4-chloro-quinazoline in acetonitrile (MeCN), using an excess of potassium carbonate (1:3) to achieve the desired alkaline pH [26]. The synthesis of thiosemicarbazide derivatives ***1a–10a*** was previously reported by several research groups [26,28,29,30,31,32,33]. The applied method by our group was to synthesize the thiosemicarbazide derivatives starting from the corresponding isothiocyanates in reaction with an excess of hydrazine hydrate 80% (1:5) in EtOH, under continuous stirring at room temperature for 5 h. Isolation in good yields (45–95%) was achieved by the quantitative formation of thiosemicarbazide crystals following the precipitation in cold water and filtration over vacuum. The synthesis of the final compounds **TSC1-TSC10** was performed by a modified protocol previously applied by Marc et al. [56]. After the prior solubilization of the intermediate (**1**) in the minimal amount of EtOH under reflux condition (7 mL), thiosemicarbazide intermediates (***1–10a***) were added to the reaction in equimolar quantities (***2a–8a***, ***10a***) or with a 25% excess (***1a***, ***9a***) and 1 drop of conc. acetic acid was used as the catalyst. The reaction mixture was refluxed for 5 h until the quantitative formation of insoluble thiosemicarbazone derivatives **TSC1-TSC10** as indicated by TLC. For the case of **TSC1** and **TSC9**, the initial low yield archived (>20%) was addressed using a 25% excess of thiosemicarbazide derivatives (***1a***, ***9a***) that were easily removed from the final products through the filtration of the hot suspension and repeated EtOH rinsing (final yield 62% and 55%). Superior yields were achieved for the aromatic derivatives **TSC2-TSC8** and **TSC10** (75–95%) following a similar isolation protocol from the hot suspension. All the final compounds (**TSC1-TSC10**) were purified through crystallization using hot EtOH.

All the novel thiosemicarbazone derivatives **TSC1-TSC10** were characterized by spectral data (Supplementary Materials, Figures S1–S40) and all the provided results indicate consistency between the synthesized compounds and proposed chemical structures. In all the provided MS spectra of the **TSC1-TSC10** series, the molecular peak was designated as [M + H]^+^. The disappearance of the high-intensity band corresponding to C=O at 1702 cm^−1^ in the FT-IR spectra confirms the successful derivatization of the aldehyde group of the intermediate (**1**) and the formation of the imine (-C=N-) bond. Two distinctive asymmetric N-H stretch bands corresponding to the thiosemicarbazone moiety can also be designated in the FT-IR spectra, at 3154–3168 cm^−1^ and 3352–3263 cm^−1^, respectively. The higher frequency band can be associated with the resonance phenomenon of the imine group that provides a higher electron-withdrawing effect to the -NH- group compared to distal substituted aromatic or aliphatic groups that exert a lower electron-withdrawing effect on the adjacent -NH- moiety. The conserved presence among the **TSC1-TSC10** series and the intermediate (**1**) of specific bands corresponding to methoxy groups (C-O str. 1207–1215 cm^−1^) and quinazoline core (C=C str. 1498–1510 cm^−1^; C=N str. 1614–1620 cm^−1^) confirms the integrity of the conserved 6,7-dimethoxy-4-substituted-quinazoline moiety. Distinct signals designated for C-F bond str. (**TSC2**, *3*-CF_3_, 1164 cm^−1^; **TSC3**, *4*-F, 1166 cm^−1^; **TSC6**, *3*-CF_3_, 1198 cm^−1^; and **TSC7**, *3,4*-diF, 1155 cm^−1^) and C-Cl bond str. (**TSC4**, *4*-Cl, 768 cm^−1^) can be identified for the series confirming the successful completion of the final step in the synthetic procedure.

In the ^1^H-NMR spectra for the **TSC1-TSC10** series, all the corresponding peaks for each compound were identified with the predicted multiplicity and coupling. The consistent presence of all the expected peaks for the 6,7-dimethoxy-4-aryloxy-quinazoline moiety, such as the Q-H_2_ singlet (8.566–8.577 ppm), two singlets corresponding to 6,7-dimethoxy moieties (3.982–3.987 ppm and 3.996–4.002 ppm), and diverse multiplicity peaks corresponding to Q-H_5_, Q-H_8_, and the aryloxy region, respectively, outlines the common origin of the synthesized compounds. The presence of distinctive peaks designated to the N-substituted thiosemicarbazone moiety confirms the completion of the final synthetic step. The provided data suggest that the electronic properties of the N-substituent play a pivotal role in the intensity of the deshielding effect on both the substituent itself and, at a distance, on the thiosemicarbazone and aryloxy protons. The highest degree of deshielding effect is induced by phenyls substituted with potent electron-withdrawing groups (**TSC2, TSC6**) that are exerted on all three protons of the thiosemicarbazone moiety (-NH-, -NH-N=, -CH=N-) and the aryloxy group. The inferior potential was identified for the EDG substituted derivative **TSC8**, which provides a diminished effect compared to all aromatic derivatives including the unsubstituted derivative **TSC5**. As expected, all the aromatic derivatives (**TSC2-TSC8, TSC10**) exhibited a significant deshielding effect when compared to the aliphatic derivatives (**TSC1, TSC9**), with the intensity of the effect being associated with the intramolecular distance (-NH- > -NH-N= > -CH=N- > Ar^1^-H_2_).

Similar observations were recorded for ^13^C-NMR spectra, where all the predicted peaks for the N-substituted thiosemicarbazone moiety were identified with corresponding values. Conserved peaks across the **TSC1-TSC10** series for -CH=N- (141.181–143.022 ppm) and C=S (175.723–177.214 ppm) confirm the successful formation of the thiosemicarbazone moiety and the synthesis of the desired compounds.

### 4.2. In Vitro Cytotoxicity Evaluation

The cytotoxic potential of the **TSC1-TSC10** series was evaluated on three cell lines including Ea.Hy926, HaCaT, and A375 following a 48 h exposure using the clinically approved anti-angiogenic agent **sorafenib** as the reference (Table 1). The graphical representation of the dose-dependence reduction in the cellular viability for the **TSC1-TSC10** series and **sorafenib** on the entire cell panel is provided in the Supplementary Materials, Figure S41. **Sorafenib** provides a suitable reference due to the increased affinity towards VEGFR2 and its widespread use in the clinical practice for the treatment of several types of cancer marked by a prominent role of neoangiogenesis in tumor expansion (hepatocellular carcinoma and renal cell carcinoma) [57].

Cytotoxic potential towards vascular endothelial cells is a suggestive indicator of a potential anti-angiogenic derivative developed as a VEGFR2 TKI; the degree of proliferation and differentiation of these cells is critical for tumor angiogenesis [58]. Ea.Hy926 cells provide a suitable in vitro model due to their stable characteristics, ability to mimic endothelial cell behavior, VEGFR2 dependency, and immortality for the evaluation of the anti-angiogenic potential of the synthesized series [59].

Eight out of the ten novel quinazoline derivatives synthesized displayed a marked antiproliferative activity on Ea.Hy296 cells (7.47–49.9 µM). The inferior cytotoxic potential was identified for **TSC2** across the entire cell panel (>100 µM) due to the highly limited solubility in the cellular environment that induced a lack of proportionality in the dose–cellular viability curve. **TSC5** displayed the highest cytotoxic potential (7.47 µM), 1.35-fold higher than the reference drug **sorafenib** (10.15 µM). Considering the aliphatic series, isopropyl derivative **TSC9** displayed superior antiproliferative activity (12.57 µM) compared to allyl derivative **TSC1** (49.9 µM), with the second-highest antiproliferative activity of the series and higher activity compared to all the phenyl-substituted derivatives of the series.

The available data suggest that the substitution pattern on the distal phenyl moiety plays a pivotal role in the cytotoxic potential towards Ea.Hy296 cells. Substitution with one or more chlorine groups (**TSC4**, **TSC6**, and **TSC10**) enhances the antiproliferative activity (12.79–16.49 µM) compared to fluorine derivatives (**TSC3**, **TSC7**) that displayed inferior cytotoxicity (26.51 µM, 43.19 µM) and *3*-methoxy derivative **TSC8** that displayed no toxicity at all (>100 µM). A *1,2*-diCl substitution pattern (**TSC10**) provides the highest potency (12.79 µM) similar to that of isopropyl derivative **TSC9**, followed closely by the mono *4*-Cl substitution (**TSC4**—14.47 µM). Further substitution with the *3*-CF_3_ group of the *4*-chlorine derivative **TSC4** in the compound **TSC6** led to a decreased cytotoxic potential (16.49 µM).

A further classification of the series can be provided by the assessment of the toxic potential toward normal keratinocyte cell line HaCaT. Skin toxicity is a widespread side effect of several anti-angiogenic compounds frequently used in clinical practice due to several off-target effects. Preliminary assessment of the toxicity through IC_50_ and the selectivity index (SI_a_) provides a predictive tool for lead molecule selection [26,60]. The entire **TSC1-TSC10** series displayed higher IC_50_ values on HaCaT cells (>15.94 µM) compared to the reference drug **sorafenib** (12.87 µM). Considering the selectivity ratio SI_a_, the three most active compounds of the series toward Ea.Hy296 (**TSC5**, **TSC10**, and **TSC9**) provided a significantly higher selectivity (2.20, 4.09, and 6.90) compared to the reference drug **sorafenib** (1.27). Lower values of SI_a_ could be suggestive of non-specific toxicity related to the increased lipophilicity of selected compounds (**TSC4**, **TSC6**).

The cytotoxic potential towards cancer cells associated with amplified VEGFR2 signaling provides a dual antitumoral mechanism for all the established VEGFR2 TKIs used in clinical practice, along with the anti-angiogenic potential [61]. In this work, we evaluated the cytotoxic potential of the series toward malignant melanoma cells A375, which could provide an effective in vitro model due to the established role of VEGFR2 signaling as a major driving oncogene in this type of cancer. Three out of the ten compounds of the series displayed visible cytotoxic potential (14.82–28.57 µM) with a lower potency compared to the reference drug **sorafenib** (5.26 µM). A similar SAR pattern can be outlined for A375, with the presence of either unsubstituted distal phenyl moiety (**TSC5**) or chlorine-substituted phenyl moiety (**TSC4**, **TSC6**, and **TSC10**) being pivotal for the cytotoxic potential. An in-depth evaluation of the selectivity across the entire cell panel identified **TSC10** as the most promising compound, displaying a comparable SI_b_ (1.83) to that of the reference drug **sorafenib** (2.45) combined with a superior selectivity between the Ea.Hy296 and HaCaT cells.

### 4.3. In Vitro VEGFR2 Kinase Inhibition Assay

All the synthesized compounds **TSC1-TSC10** and **sorafenib** were assessed for the potential to inhibit the kinase activity of VEGFR2 in vitro and the results are indicated in Table 1. At an initial screening of the series at 500 nM, six out of the ten quinazoline derivatives provided an inhibitory potential higher than 50% of the kinase activity as indicated by the assay. The available data suggest that the 4-chloro-substitution of the distal phenyl ring leads to a dramatic decrease in the inhibitory potential as indicated by **TSC4** (40.49% inhibition) with a total loss of the activity with the further inclusion of an additional 3-CF_3_ group in the case of **TSC6** (0% inhibition). Electron-donating groups such as 3-methoxy substitution for **TSC8** (0% inhibition) and the dual 3,4-difluoro-substitution for **TSC7** (21.09% inhibition) were identified to be unfavorable for the biological activity. Out of the entire series, **TSC10** stands out with the lowest IC_50_ value (119 nM), which is less potent than the reference drug **sorafenib** (8.99 nM). Aliphatic derivatives **TSC1** (IC_50_ = 389 nM) and **TSC9** (IC_50_ = 378.68 nM) displayed a medium inhibitory potential towards VEGFR2 in a similar range with the unsubstituted aromatic derivative **TSC5** (IC_50_ = 378.13 nM). An interesting case is that of the compound **TSC2** (IC_50_ = 340.78 nM), which displayed no antiproliferative activity at all due to the limited solubility in the growth media but is capable of inhibiting in the nM range of the VEGFR2 kinase activity due to the reduced relevance of the solubility for the concentrations used in this assay.

### 4.4. Wound Healing (Scratch) Assay

**Figure 3:**
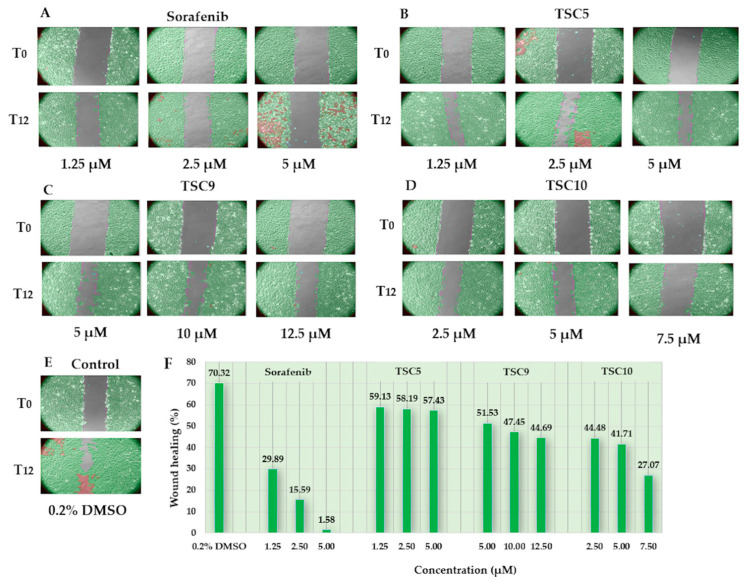
Ea.Hy296 wound closure after 12 h (T_12_): after treatment with **sorafenib** (**A**); after treatment with **TSC5** (**B**); after treatment with **TSC9** (**C**); after treatment with **TSC10** (**D**); and after treatment with 0.2% DMSO (NC) (**E**). Graphical representation of the percentage of wound closure (**F**) (%) after 12 h for each evaluated compound (**sorafenib**, **TSC5**, **TSC9**, and **TSC10**) and the designated evaluated concentration.

Cellular motility is a crucial hallmark of angiogenesis that is initiated by the formation on the leading tip of the vascular endothelial cell of distinct membrane protrusions named lamellipodia and filipodia. VEGFR2 signaling plays a pivotal role as a major motility inducer through extensive cytoskeletal rearrangements and enhancement of the focal adhesion turnover through complex interactions with integrins, acting as a chemotactic agent in response to the VEGF concentration gradient in the hypoxic areas [62,63]. An experimental in vitro model was employed to evaluate the potential of the most promising compounds (**TSC5**, **TSC9**, and **TSC10**) to inhibit the motility of Ea.Hy296 cells to further evaluate their anti-angiogenic potential. The evaluated compounds were selected based on their increased cytotoxic potential towards Ea.Hy296 cells (7.47–12.79 µM), promising VEGFR2 inhibition in vitro and the highly favorable selectivity index SI_a_ in comparison with the reference drug **sorafenib**. A series of three concentrations, lower than the experimentally determined IC_50_ values, were tested in this assay with the experimental results being depicted in Figure 3.

**Sorafenib** displayed the highest capability to inhibit the mobility of Ea.Hy296 cells in a concentration-dependent manner, with an almost total inhibition at the highest evaluated concentration (5 µM). Out of all three quinazoline derivatives, **TSC5** displayed the lowest motility inhibition potential (11.19–12.82%) with a low concentration dependence correlation. Superior results were achieved in the case of **TSC9** and **TSC10**, respectively, with a reduction in the wound closure area of 25.63% and 43.29% for the highest evaluated concentration. By reporting these values to the normal motility of the Ea.Hy926 cells (% wound closure with negative control 0.2% DMSO), the experimental data suggest the reduction of the wound closure speed by 2.6-fold for the derivative **TSC10** at the 7.50 µM concentration.

### 4.5. Chorioallantoic Egg Membrane (CAM) Assay

**Figure 4:**
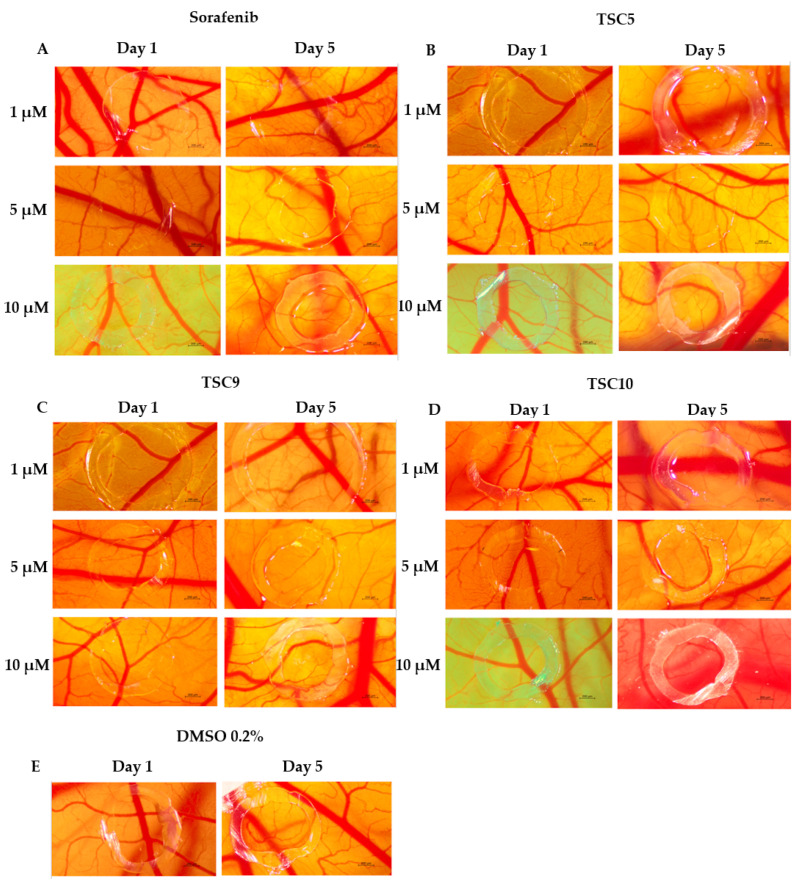
The impact on angiogenesis in the CAM assay following the treatment with different concentrations (1 μM, 5 μM, and 10 μM) of (**A**) **sorafenib**, (**B**) **TSC5,** (**C**) **TSC9**, (**D**) **TSC10**, and (**E**) **DMSO 0.2%**. The graphical representation indicates day 1 of the experiment compared to day 5 of the consequent exposure to the tested samples.

In the assessment of the anti-angiogenic potential of the selected compounds of the series (**TSC5**, **TSC9**, and **TSC10**—similar selection criteria to Section 4.4) along with the reference drug **sorafenib** and the negative control (**0.2% DMSO**), the development of the vascular architecture of the CAM was evaluated during the 5-day protocol following the consecutive administration of the tested samples. All the evaluated compounds were tested at three distinctive concentrations (1 µM, 5 µM, and 10 µM) with no visible signs of vascular irritation such as lysis, hemorrhage, or coagulation across the entire panel of experiments. A comparison between the first and fifth days of the experiment is provided in Figure 4 for all the evaluated compounds at the three concentrations while the entire panel of photos (day 1 to day 5) is available in the Supplementary Materials, Figure S42.

For the negative control (**0.2% DMSO**), a normal angiogenic process was observed. The architectural arrangement of the vascular plexus within the treatment region remained similar to that of the surrounding area outside the delimited ring. Notably, the development of new blood vessels was highly visible, particularly on day 4 of the experiment. Both the formation of new vascular branches and their optimal development were observed, with the vessels progressively increasing in diameter over time.

In the case of **sorafenib**, a potent anti-angiogenic effect was observed across all three tested concentrations. The strongest and most pronounced anti-angiogenic activity was noted at the highest concentration of 10 μM. These anti-angiogenic effects were observable as early as day 2 of treatment and intensified with each subsequent day. Specifically, it was evident by day 2 that no new blood vessel branching occurred, and the diameter of the existing vessels failed to increase, remaining stagnant in their growth. Furthermore, by day 5 of treatment, the vessels appeared thinner and more fragile. A similar phenomenon occurred at 5 μM with a noticeable impact on the vascular growth and minimal impact at 1 μM, suggesting a strong correlation between the biological effect and the concentration of the tested sample.

For compound **TSC5**, a pronounced decrease in CAM vascularization was identified. The anti-angiogenic effects were highly visible as early as day 2 for the 5 μM and 10 μM concentrations and by day 3 for the 1 μM concentration. Throughout the treatment, the blood vessels within the vascular plexus became increasingly thinner, with a significant reduction in vessel diameter observed by day 5 across all three tested concentrations. Notably, the formation of new blood vessel branches was not observed, indicating that neoangiogenesis was significantly inhibited by **TSC5**. The results obtained with **TSC5** were comparable to those induced by **sorafenib**, with a slightly higher biological effect at the lower doses (1 μM).

For **TSC9**, the anti-angiogenic properties were also demonstrated across all three tested concentrations. Similar to the findings of **TSC5**, the two higher concentrations (5 μM and 10 μM) produced stronger anti-angiogenic effects. However, the lowest concentration of 1 μM was also able to inhibit neoangiogenesis throughout the treatment period. For **TSC9**, the anti-angiogenic effects became much more pronounced and visible in the later stage of the experiment (days 4–5), with a slightly lower impact at the smaller concentration of 1 μM, as compared to all the other tested compounds.

In the evaluation of **TSC10**, potent anti-angiogenic properties were observed. This compound significantly reduced the development of CAM vascularization within the treatment area. The vessel diameter was progressively reduced, eventually resulting in very thin and fragile blood vessels. The anti-angiogenic effects elicited by **TSC10** were highly comparable to those obtained with the positive control, sorafenib treatment, and the **TSC5** derivative. A lower intensity effect was identified for 1 μM starting from day 3, with a highly potent inhibition by day 2–3 for the 5 μM and 10 μM concentrations with an almost complete disruption of the vascular development by the end of the experiment.

Considering the above findings, all the evaluated compounds displayed a significant inhibition of the angiogenic potential, with two out of the three novel compounds (**TSC5** and **TSC10**) displaying a similar effect to that of the reference compound **sorafenib** both in potency and in the concentration–biological effect dependency. An overview of the experimental data suggests a faster onset of the anti-angiogenic effect for **TSC5** compared to **TSC10** and **sorafenib**, which provided progressive inhibition over time. Concerning the potential clinical implications of this phenomenon, a stronger onset of the inhibition could be related to a higher risk of hypoxia-driven tumor resistance and a lower safety profile, while a progressive profile could be related to a more sustained control over time for the chronic treatment [64].

### 4.6. ADMEtox and Drug-Likeness Profile

The physicochemical and drug-likeness potential of the novel compounds **TSC1-TSC10** and reference drug **sorafenib** evaluated by the SwissADME web tool are depicted in the Supplementary Materials, Table S1. The available data indicate that all the compounds display a minimal number of violations of the Lipinski rule of five or the Veber filter, which provides positive evidence for oral absorption. Three compounds of the series displayed higher than 500 g/mol molecular weight (**TSC2**, **TSC6**, and **TSC10**), which is related to the multiple halogen substituents in each molecule. Overall, the series displayed a high degree of lipophilicity and reduced water solubility (“poorly” or “moderately” soluble, with the lowest solubility predicted for **TSC2**), which is related to the increased number of aromatic moieties (at least three) that are consecrated to be the minimal requirement for VEGFR2 inhibition through the type II mechanism. A better insight into the oral absorption capabilities of the compounds can be provided by the graphical representation of the bioavailability radar (Supplementary Materials, Figure S43).

The pharmacokinetics profile of **TSC1-TSC10** and **sorafenib** were evaluated by both the SwissADME web tool and the pkCSM web tool and the results are depicted in the Supplementary Materials, Table S2. The overall gastro-intestinal (GI) absorption predicted by SwissADME is higher or similar to that of **sorafenib** (“Low” or “High”). The theoretical Caco2 (immortalized human colorectal adenocarcinoma cells) permeability model employed by pkCSM suggests a significantly higher penetration of the cellular monolayer for **TSC1-TSC10** (1.008–1.134 Log Papp in 10^−6^ cm/s) compared to **sorafenib** (0.302 Log Papp in 10^−6^ cm/s) and no interactions with P-glycoproteins. All the evaluated compounds are predicted to have a high protein plasma binding (0–0.135% unbound fraction) with the highest volume of distribution (VDss) being predicted for **TSC10** (0.035 LogL/Kg). All compounds displayed low to minimal blood–brain barrier (BBB) permeability and central nervous system (CNS) permeability with potential interactions with the CYP3A4 enzymatic system.

The toxicological profile predicted by the pkCSM web tool is depicted in the Supplementary Materials, Table S3. No compound displayed mutagenic potential with all the **TSC1-TSC10** series (except **TSC9**) displaying higher predicted maximum tolerated doses in humans (0.545–0.77 log mg/kg/day) compared to **sorafenib** (0.16 log mg/kg/day). No compound was predicted to be related to skin sensitization and all the compounds (except for **TSC10**) were identified as potential hepatotoxic agents. The other predicted parameters such as acute or chronic rat toxicity suggest a better toxicity profile compared to **sorafenib**, while the toxic potential towards *T. pyriformis* and minnows is similar across all the evaluated compounds.

### 4.7. Molecular Docking Studies

**Figure 5:**
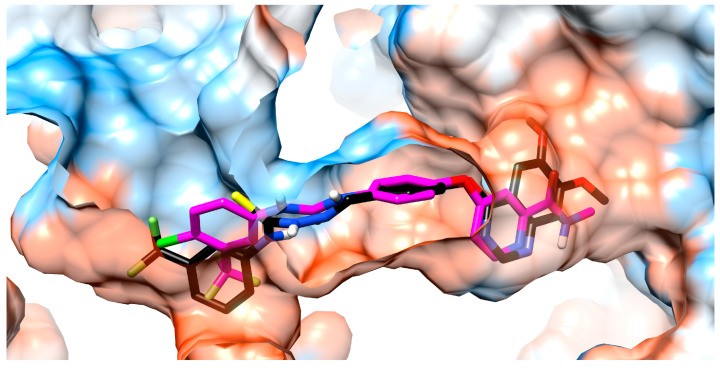
**TSC2** (black) docked into the catalytic site of the VEGFR2 (DFG-out configuration) superposed onto the docked position of **sorafenib** (magenta) in a 3D surface visualization.

The novel quinazoline derivatives **TSC1-TSC10** and **sorafenib** were docked into the ATP-binding site of the kinase domain of the inactive form of VEGFR2 (DFG-out). Preliminary analysis of the results suggests an increased level of similarity between the binding pattern predicted for the **TSC1-TSC10** series and **sorafenib**, with the high degree of superposition indicating the successful application of the scaffold hopping approach in the development of the novel compounds (Figure 5).

**Figure 6:**
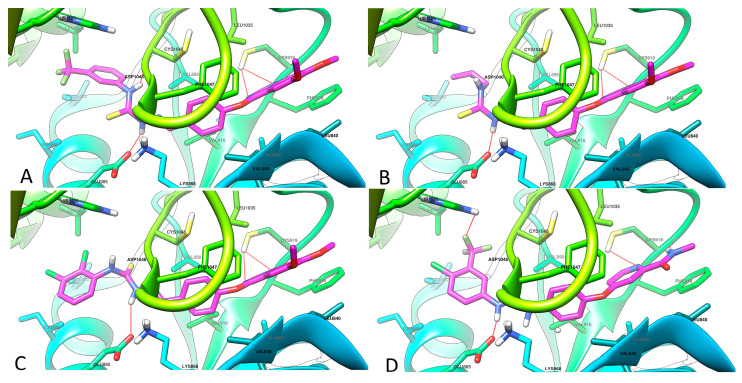
Graphical representation of the binding mode of **TSC2** (**A**); **TSC9** (**B**); **TSC10** (**C**); and **sorafenib** (**D**) in the catalytic site of the VEGFR2 (DFG-out) kinase domain [4ASD] using UCFS Chimera 1.10.2. For each compound, the chemical framework is colored with magenta, and H-bonds are indicated with bright red lines.

Both the quinazoline (**TSC1-TSC10**) and pyridine (**sorafenib**) cores successfully accommodate the HYDI, stabilizing the complex though acceptor H-bonds provided by Cys919 and hydrophobic contacts with Leu840, Glu917, Phe918, respectively (Figure 6). Similarly, the 6,7-dimethoxy groups on the quinazoline core and the 2-methyl-amide moiety of **sorafenib** extend into the Extra I region of the kinase domain. The 4-phenoxy substituent linked to the quinazoline (**TSC1-TSC10**) or pyridine (**sorafenib**) core enters through the gatekeeper region, stabilizing the complex by multiple hydrophobic bonds (Leu840, Ala866, Val899, Val868, Phe918, Phe1047, and Leu1035). The ether bridge that links the two aromatic moieties provides a suitable angle between the two plans that facilitates the complex formation with minimal repulsion from the gatekeeper residues (Val 916, Lys868). Overall, the 4-phenoxy-quinazoline moiety (**TSC1-TSC10**) and 4-phenoxy-picoliamide (**sorafenib**) displayed an almost complete overlap that provides a solid anchor for the VEGFR2–ligand complex and substantial stability.

The complex electronic properties of the thiosemicarbazone (**TSC1-TSC10**) linker provide a series of distinctive features compared to the urea group (**sorafenib**). The extended hybridization induces a zig-zag conformation with the two hydrogens of the -NH- pointing towards the opposite parts of the group plan. In the entire **TSC1-TSC10** series, one -NH- group points toward Glu885, leading to the formation of a donor H-bond through the disruption of the Lys868-Glu885 salt bridge, and one -NH- group points toward the Asp1047 forming a stable H-bond with the side chain of the residue, leading to the successful accommodation of the DFG region. Canonically, **sorafenib** binds the Glu885-Asp1047 pair through a donor–acceptor H-bond interaction by the urea group in a similar binding pattern predicted for the **TSC1-TSC10** series.

The distal substituent of the hydrophilic linker on the urea group of the **sorafenib** (4-Cl-3-CF_3_-benzene) or thiosemicarbazone group (aliphatic or aromatic groups for the **TSC1-TSC10** series) successfully dives into the allosteric pocket of the kinase domain (HYD II) formed by the displacement of the Phe1047 from the DFG motif (inactive conformation). In the case of **sorafenib**, the substituted distal phenyl residue forms stabilizing hydrophobic interactions with His1026, Leu1019, Ile888, and Ile852. The different lengths of the hydrophilic linker in the DFG region (thiosemicarbazone vs. urea) induce a slight shift forward, deeper in HYD II, of the distal substituent for the **TSC1-TSC10** series that leads to novel interactions in the VEGFR2–ligand complex compared to **sorafenib**.

Aliphatic groups (allyl—**TSC1**, isopropyl—**TSC9**) displayed the lowest affinity towards the VEGFR2 (DFG-out) kinase domain with a binding energy of ∆G = −9.1 and −9.3 kcal/mol. The predicted affinity of the phenyl-substituted derivatives was substantially higher (∆G = −9.6 to −10.9 kcal/mol), which could indicate the favorable effect of an aromatic moiety in this region. The experimental data suggest that the substitution pattern on the phenyl moiety plays a crucial role in the affinity for the target and the orientation in the HYD II. The closer proximity of His1026 and Leu1019 compared to **sorafenib** leads to different binding patterns due to the repulsive forces. Smaller substituents in *para* (*4*-F, **TSC3**, ∆G = −10 kcal/mol) or the unsubstituted phenyl ring (**TSC5**, ∆G = −10.1 kcal/mol) lead to an orientation similar to **sorafenib**, with minor deviation of the phenyl group plan. Bulkier groups (*4*-Cl, **TSC4**, ∆G = −10 kcal/mol; *4*-Cl-*3*-CF_3_, **TSC6**, ∆G = −10.9 kcal /mol) can lead to dramatic shifts in the phenyl moiety orientation, pointing toward Arg1027 in the opposite direction of the HYD II. An intermediary level of steric hindrance provided by the *2,3*-diCl substitution of **TSC10** (∆G = −9.6 kcal/mol) provides a medium degree of shift in the phenyl group position (Figure 6). An interesting case is that of **TSC2** (*3*-CF_3_, ∆G = −10.5 kcal/mol), which displays an increased level of affinity toward VEGFR2 kinase (DFG-out), with the phenyl group oriented similarly with **sorafenib** (with a slight deviation) but flipped, with the *3*-CF_3_ pointing in the opposite direction compared to the same group of **sorafenib**, away from His1026. This increased level of variability is possible due to the flexible nature of the -NH-N= bond of the thiosemicarbazone moiety of the **TSC1-TSC10** series. For a more comprehensive understanding of the interactions between the entire TSC1-TSC10 series and the active site of the VEGFR2, 2D depictions are provided in the Supplementary Materials, Table S4.

### 4.8. Quantum Chemistry Parameters

Quantum chemistry descriptors (frontal molecular orbitals—FMOs, chemical reactivity descriptors—CRDs, and dipole momentum) are a powerful tool in drug discovery for the prediction of reactivity and hotspots of electron interactions with a biological target in conjugated π-systems [45,65]. A facile evaluation of the electronic interaction in a ligand-biological target is provided by the 3D distribution of the highest occupied molecular orbital (HOMO) and lowest occupied molecular orbital (LUMO) and the complementarity between the proximal regions found in contact in the given complex [66]. The FMO analysis, calculated CRDs, and dipole momentum for **TSC1-TSC10** and **sorafenib** are provided in the Supplementary Materials, Table S5.

**Figure 7:**
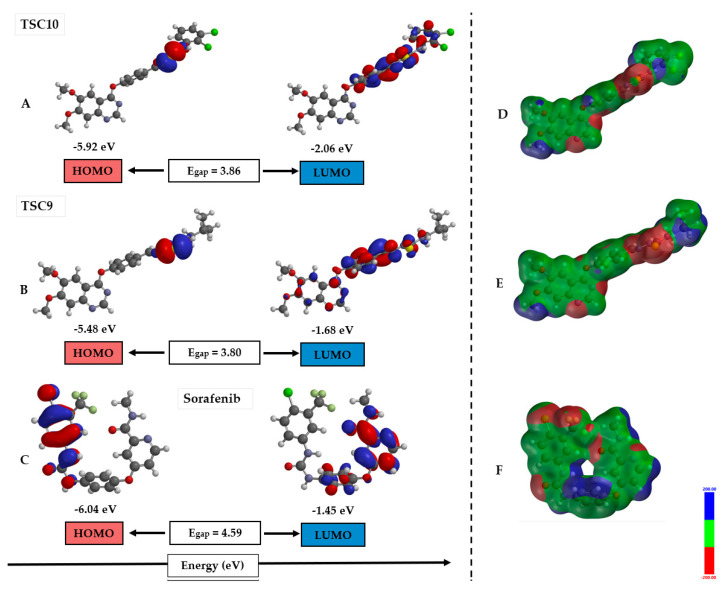
Schematic representation of the HOMO and LUMO orbital surface distribution and energy levels for **TSC10** (**A**), **TSC9** (**B**), and **sorafenib** (**C**). Graphical representation of the MEMs for **TSC10** (**D**), **TSC9** (**E**), and **sorafenib** (**F**).

The HOMO-LUMO spatial distribution for selected compounds (**TSC9**, **TSC10**; for **TSC1-TSC8**, see the Supplementary Materials, Table S6) and **sorafenib** is provided in Figure 7. As expected, due to the increased electronegativity and polarizability of the sulfur and -CH=N- group, for the entire **TSC1-TSC10** series, the HOMO orbitals are confined around the thiosemicarbazone moiety. Increased HOMO energy is associated with electron-donating regions, which, in this case, led to the polarization of the N-H bonds that could facilitate the donor H-bond interactions with the Glu885 residue as predicted by the VEGFR2–ligand interaction studies. For the distal aromatic substituted derivatives (**TSC2-TSC8**, **TSC10**), LUMO orbitals are confined on top of the two phenyl rings and the thiosemicarbazone group due to the extended π-system conjugation of the electronic cloud. For the aliphatic derivatives (**TSC1**, **TSC9**), the LUMO system is extended across the entire molecule due to the lack of π-conjugation, providing less stable and higher energy LUMO orbitals (Supplementary Materials, Table S5) (−1.73 eV, −1.68 eV) compared to the highly electron-withdrawing groups such as *4*-Cl-3-CF_3_ (**TSC6**, −1.14 eV) or 2,3-diCl (**TSC10**, −2.06 eV).

The high level of overlapping of the HOMO-LUMO orbitals in the **TSC1-TSC10** series provides an effective charge transfer transition and makes the thiosemicarbazone moiety a hotspot for potential interactions with the biological target. Similar phenomena are predicted for **sorafenib**, but due to the less electronegative character of the oxygen atom in the urea group, HOMO orbitals (−6,04 eV) are extended on the entire *4*-Cl-*3*-CF_3_-phenyl-urea moiety and LUMO orbitals are confined around the π-deficient picoliamide moiety (−1.45 eV).

The **TSC1-TSC10** series displays a smaller HOMO-LUMO gap (3.78–3.91 eV) compared to **sorafenib** (4.59 eV), indicating a higher degree of polarization, which is due to “soft molecules” with a decreased chemical hardness. Charge separation in the molecules is indicated by the dipole momentum, with **TSC10** (6.13 Debye) and **sorafenib** (6.68 Debye) displaying the most effective separation with a potential impact on biological activity. All the compounds are strong electrophiles (ω = 3.37–4.29 eV, ω > 1.5 eV) compared to **sorafenib** (3.06 eV) and marginal nucleophiles (N = 0.23–0.33 eV^−1^) based on Domingo’s scale, as previously reported [67]. The molecular electrostatic maps (MEMs) are also provided in Figure 7 (**TSC9**, **TSC10**, and **sorafenib**; for **TSC1-TSC8**, see the Supplementary Materials), providing a graphical representation of the electron-rich areas (red) and electron-deficient areas (blue).

### 4.9. Molecular Dynamics Studies

Further analysis of the ligand–VEGFR2 complex interactions and their stability can be achieved by MD studies. The evaluation of the physical movement in a sampled system of the atoms involved in the evaluated complex provides insight into the stability and behavior of the ligand–biological target interactions. The quantitative assessment of the trajectories of the atoms evaluated during the 100 ns simulations of the **TSC1-TSC10** series and **sorafenib** in complex with VEGFR2 were analyzed and expressed as the root mean square deviation (RMSD) of the ligand’s heavy atoms, the RMSD of the protein, the radius of gyration of the protein (Rg-protein), the root mean square fluctuation (RMSF) of the α-carbons and the AA side chains, and the overall predicted number of hydrogen bonds (NoHB) formed between the ligand and protein during the 100 ns simulations (Table 2).

In the aromatic series (**TSC2-8,10**), the available data suggest that the substitution pattern on the distal phenyl group plays a significant role in the stability of the ligand–VEGFR2 complex. The lowest stability in terms of RMSD-ligand was predicted for the compounds with bulky substituents in position 4 of the benzene ring (**TSC4**—0.48 nm, **TSC6**—0.38 nm). Lower stability was also provided by electron-donating groups in position 3, such as the methoxy group (**TSC8**—0.33 nm). Compared to unsubstituted derivative **TSC5** (0.25 nm), higher stability is achieved with the insertion in position 4 and/or 3 of medium-sized lipophilic groups (*4*-F, **TSC3**—0.21 nm; *3,4*-diF, **TSC7**—0.25 nm). In all cases, the magnitude of instability of the ligand–VEGFR2 complex predicted by the movement of the heavy atoms is highly correlated with the protein structure’s related parameters (Table 2). Aliphatic substituted thiosemicarbazone derivatives **TSC1** and **TSC9** displayed an intermediary stability slightly higher than that of the unsubstituted phenyl derivative **TSC5**. Graphical representation of the parameters in time during the 100 ns simulations for **TSC1,3-9** are provided in the Supplementary Materials, Figures S44–S47. The depiction of the pose of the ligands (**TSC1-TSC10**) at the beginning of the simulation compared to the end of the simulation is provided in the Supplementary Materials, Table S7.

**Figure 8:**
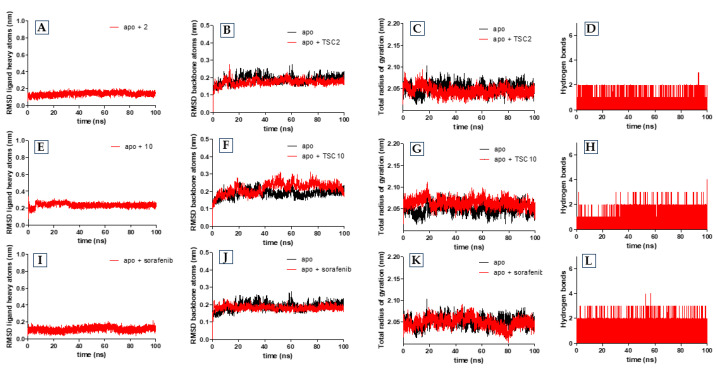
The evolution in time of the MD parameters during the 100 ns simulations for the selected compounds: **TSC2** ((**A**) RMSD-ligand, (**B**) RMSD-protein, (**C**) Rg-protein, and (**D**) NoHB generated in the ligand complex during the simulation); **TSC10** ((**E**) RMSD-ligand, (**F**) RMSD-protein, (**G**) Rg-protein, and (**H**) NoHB generated in the ligand complex during the simulation); and **sorafenib** ((**I**) RMSD-ligand, (**J**) RMSD-protein, (**K**) Rg-protein, and (**L**) NoHB generated in the ligand complex during the simulation).

The highest degree of stability in the series was predicted for the **TSC2**-VEGFR2 complex, with the lowest value for RMSD-ligand (14 nm) comparable to that of **sorafenib** (12 nm), keeping its initial docked state with minimal movement during the entire 100 ns simulation. The impressive stability over the 100 ns simulation was also confirmed by a lower impact on the motion of the protein’s atoms (RMSD protein = 0.15 nm, Rg-protein = 0.14 nm) than **sorafenib**, suggesting a stabilizing effect compared to the normal state of the protein in similar conditions. In terms of fluctuation of the α-carbons and side chains, a minimal movement suggests, for both **sorafenib** and **TSC2,** a high degree of stability for the complex with a reduced impact on the AAs′ topological positions. The graphical representation for **TSC2** and **sorafenib** of these parameters and their evolution during the 100 ns simulations is provided in Figure 8.

Intriguing results were identified in the case of the **TSC10**-VEGFR2 complex. While the RMSD-ligand value suggests average stability of the complex (0.22 nm), the graphical representation of this parameter indicates an initial unstable state with increased movement of the heavy atoms (0–30 ns) followed by a highly stable state in the second part of the simulation (Figure 8E). An in-depth analysis of the simulation’s frames indicates that the initial docked state of **TSC10** in the active site of VEGFR2 is highly unstable due to the unfavorable proximity of the *2,3*-diCl substituents and the carboxyl group of Glu885. The initial repulsion between the two electronegative entities led to a dramatic shift in both the position of the *2,3*-diCl-phenyl group of **TSC10** with a 90° flip (into a similar position to that of **TSC2′**s 3-CF_3_-phenyl group) and the negatively charged side chain of Glu885. The novel orientation of the distal aromatic ring of **TSC10** buried in a hydrophobic pocket (Gly893, Leu889) provides an increased state of stability for the **TSC10**-VEGFR2 complex, followed by the return of the Glu885 carboxyl group in the initial position. The conformation modifications in the protein structure are visible in the RMSD-protein graphical representation, suggesting an increased movement after the first 40 ns (Figure 8F). The initial state of instability is also suggested by the ascending radius of gyration (0–20 ns), followed by minimal modifications from the baseline in the second part of the simulation (Figure 8G).

The overall low NoHB protein–ligand interactions during the simulation of the evaluated series (0.07–0.44) compared to sorafenib (1.00) suggest that one of the main driver forces for complex stability is hydrophobic interactions. Out of all the compounds, **TSC10** displays the highest NoHB interactions (0.44), with a unique pattern of increasing the number after the first 30 ns of the simulation (Figure 8H). An in-depth evaluation of the frequency of the individual H-bonds with specific AAs of VEGFR2 is provided in the Supplementary Materials, Table S8. The experimental data suggest a similar pattern of H-bond interactions between **TSC1-TSC10** and **sorafenib** but with a highly different distribution during the 100 ns simulations. **Sorafenib** displayed a minimal interaction with Cys919 (1.78%), with a higher dependency for the complex stability of the Glu885-Asp1046 pair (38.25%, 58.33%). For **TSC2**, a similar pattern was observed with a significantly higher affinity for Cys919 (16.59%) and a different distribution of Glu885-Asp1046 (7.66%, 0.49%). A distinctive feature for **TSC2** and the aliphatic derivatives (**TSC1**, **TSC9**) is the formation of an increased number of H-bond interactions with the gatekeeper Lys868 (8.22–9.71%). **TSC10** displays a unique pattern with an even distribution between Cys919 (15.12%), Glu885 (12.54%), and Asp1046 (13.74%). The analysis of the H-bond distribution during the four quarters of the simulation (Supplementary Materials, Table S9) indicates that the *2,3*-diCl-phenyl group drift in the first 30 ns led to a highly favorable state that facilitates H-bond formation with all three major residues (Cys919, Glu885, and Asp1046), which are canonically known to drive type II inhibitor complex formation.

### 4.10. MM-PBSA Energy Decomposition

For a better understanding of the interactions concerning the complex formed between the **TSC1-TSC10** series and the kinase domain of VEGFR2 (DFG-out), an MM-PBSA analysis of the last 25 ns of the MD simulations was conducted and the predicted values are presented in Table 3.

According to the ΔG values (kcal/mol), 3 out of the 10 compounds of the **TSC1-TSC10** series displayed superior binding free energy (**TSC2** = −74.24 ± 3.88 kcal/mol; **TSC9** = −72.41 ± 3.51 kcal/mol; and **TSC1** = −70.97 ± 5.31 kcal/mol) compared to the reference drug **sorafenib** (−69.39 ± 3.63 kcal/mol). In a similar pattern to the MD stability parameters, bulkier substituents in position 4 of the phenyl derivatives (**TSC4**, **TSC6**) led to the lowest total free energies recorded (−33.23 ± 8.64 kcal/mol; −35.61 ± 10.71 kcal/mol), with the highest fluctuations in terms of standard deviations (SDs) that are predictive for a highly unstable ligand–protein complex. Smaller 4-phenyl substituents such as 4-F (**TSC3** = −68.70 ± 5.86 kcal/mol) displayed superior binding free energy compared to the bulkier substituents and the 3,4-diF-phenyl derivative **TSC7** (−43.71 ± 5.60 kcal/mol). Considerably lower energy was identified for **TSC10** (−62.18 ± 4.11 kcal/mol) with a reduced SD.

A detailed analysis of the energetic contributions of the ΔG parameter suggests that for the entire **TSC1-TSC10** series and **sorafenib,** the major driving force in the ligand–VEGFR2 complex formation is the van der Waals-type interactions. An increased van der Waals energy is suggestive of a high degree of shape complementarity between the ligand and the biological target, with favorable hydrophobic contacts and minimal steric clashes. For the analyzed compounds, the most favorable van der Waals interactions were identified for **TSC2** (−69.68 ± 3.02 kcal/mol) and **TSC10** (−65.93 ± 2.78 kcal/mol), which are significantly higher than **sorafenib** (−59.19 ± 2.81 kcal/mol). Increased electrostatic energy was identified for the unstable complexes generated by **TSC4-TSC6** and **TSC8** (between −89.73 ± 30.80 kcal/mol and −123.28 ± 10.73 kcal/mol) that is associated with a highly increased positive solvation energy (114.08 ± 22.0 kcal/mol to 130.64 ± 8.82 kcal/mol) leading to an unfavorable energetic balance, which ultimately connects to complex instability due to an unfavorable desolvation phenomenon. For the complexes with increased stability according to the MD studies, the aliphatic derivatives (**TSC1**, **TSC9**) display the highest dependency towards electrostatic interactions for the complex stability (−46.82 ± 4.11 kcal/mol, −39.43 ± 3.80 kcal/mol) to a degree similar to **sorafenib** (−39.67 ± 3.76 kcal/mol). A unique pattern was identified for **TSC10,** with a low contribution for the total energy from the electrostatic interactions (−12.24 ± 4.58 kcal/mol) and the most favorable energetic penalty for the solvation (15.99 ± 3.74 kcal/mol) compared to all the evaluated complexes.

**Figure 9:**
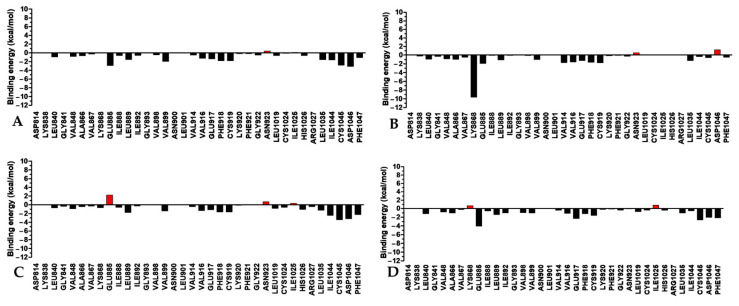
Free energy decomposition for all the AAs in the 5Å proximity of the ligand ((**A**) **TSC2**, (**B**) **TSC9**, (**C**) **TSC10**, and (**D**) **sorafenib**) during the last 2500 frames of the MD simulation.

To provide an insight into the detailed interactions with relevant AAs that are found in the 5Å proximity of the ligand in the complex, an energy decomposition was performed, and the graphical representation for **TSC2**, **TSC9**, **TSC10**, and **sorafenib** is depicted in Figure 9 (**TSC1**, **TSC3-TSC8**, Supplementary Materials, Figure S48). An overview of the experimental data for the unstable complexes **TSC4-TSC6** and **TSC8** suggests that the major driving force of instability is the electrostatic repulsion (+4.28 kcal/mol to +10.12 kcal/mol) of the positively charged side chain of the Lys868 residue found in the gatekeeper area. The impaired fit of the distal substituent on the thiosemicarbazone moiety in HYD II leads to a drift of the entire molecule towards the gatekeeper and hinge region that severely impairs the complex stability through repulsion from Lys868 but also an unfavorable position for H-bond generation with the Asp1047 side chain (**TSC5** = +3.76 kcal/mol; **TSC7** = +2.05 kcal/mol).

Aliphatic derivatives **TSC1** and **TSC9** displayed a unique stability pattern based on an increased electrostatic energy interaction with the Lys868 side chain (−11.13 kcal/mol, −9,86 kcal/mol) and a minimal contribution from Cys919, Glu885, and Asp1046. Compound **TSC2** displayed the most favorable energetic pattern with increased binding energy identified towards Cys919 (−2.80 kcal/mol), Asp1046 (−3.08 kcal/mol—major van der Waals contribution), and Glu885 (−2.89 kcal/mol—major electrostatic contribution) in a similar distribution to **sorafenib**. Like the aliphatic derivatives, the **TSC2** data suggest an increased electrostatic energy from the interactions with the Lys868 side chain (−5.17 kcal/mol) but with an overall neglectable effect (−0.07 kcal/mol) due to the increased solvation energy (+6.54 kcal/mol).

In the case of **TSC10**, a shift in the position after the first 30 ns of the MD simulation according to the MM-PBSA decomposition led to a stable state with a unique interaction with the major AAs. While there is a significant interaction with the Glu885 side chain through electrostatic (−1.78 kcal/mol) and van der Waals (−1.31 kcal/mol) energy, the overall energetic contribution suggests a negative impact (+2.21 kcal/mol) on the complex stability due to the initial drift of the side chain that was solvated in the process and requires higher energy for solvation (+5.31 kcal/mol). The final stable state seems to lead to marked electrostatic repulsion from Asp1046 (+2.57 kcal/mol), which overall has a favorable impact on the complex stability through van der Waals (−2.58 kcal/mol) and favorable solvation energy (−3.20 kcal/mol). The **TSC2**-VEGFR2 complex stable state is predicted to rely on hydrophobic interactions with AAs such as Cys919 (−3.44 kcal/mol), Leu1035 (−1.24 kcal/mol), Ile1044 (−2.43 kcal/mol), and Phe1047 (−2.25 kcal/mol).

## 5. Conclusions

In summary, ten novel thiosemicarbazone-containing quinazoline derivatives were successfully developed as potential VEGFR2 inhibitors with proven antiproliferative and anti-angiogenic potential. The selected compounds displayed remarkable potential through in vitro and in silico screening, providing a similar pharmacologic profile to that of the reference drug **sorafenib**.

The cytotoxic potential towards vascular endothelial cells provides a preliminary insight into the anti-angiogenic potential of the compounds. Eight out of the ten derivatives were identified to have an increased cytotoxic potential towards Ea.Hy296 cells (7.47–49.9 µM). The toxic potential of the series was evaluated on the HaCaT cell line, with the entire series providing a higher IC_50_ value (>15.94 µM) compared to **sorafenib** (12.87 µM); three out of the ten compounds stood out based on their superior selectivity profile, which was quantified by SI_a_. Additional experimental data on A375 melanoma cells identified **TSC10** (28.57 µM) as a promising compound of the series, with a selectivity ratio of malignant-to-healthy cell IC_50_ ratio in a similar range to that of **sorafenib** (1.83 vs. 2.45).

The initial hypothesis was verified by the in vitro VEGFR2 kinase inhibition assay, which confirmed the lack of inhibitory potential at the tested concentration (500 nM). While **sorafenib** was identified to have a 10-fold higher affinity toward the VEGFR2 kinase domain (8.99 nM), a similar SAR pattern with the antiproliferative assay was identified, with the highest inhibitory potential being recorded for **TSC10** (119 nM) and **TSC3** (239.41 nM).

The most promising compounds (**TSC5, TSC9,** and **TSC10**) were assessed for their capacity to inhibit the motility of vascular endothelial cells and their impact on the formation and differentiation of the vascular architecture in the CAM assay. **TSC10** provided the highest inhibition of cellular motility with an up to 2.6-fold reduction compared to the normal speed of Ea.Hy296 wound closure at the 7.5 µM concentration. In the CAM assay, both **TSC5** and **TSC10** displayed a remarkable capacity to reduce the vascular branching, blood vessel diameter, and blood vascular density in ovo, to a similar degree to that of **sorafenib**.

The docking studies revealed an increased level of superposition between the **TSC1-TSC10** series and **sorafenib**, successfully confirming the scaffold-hopping approach for their development. Established key interactions canonically identified to be detrimental for VEGFR2 inhibition (H-bonds with Glu885, Asp1046, and Cys919) were predicted to form and stabilize the complex for the novel compounds in a similar fashion to **sorafenib**. The HOMO-LUMO orbital distribution is highly influenced by the increased electronegativity of the thiosemicarbazone moiety.

MD studies provide insight into the stability of the ligand–VEGFR2 complex in time. The compounds with a minimal inhibitory potential towards VEGFR2 (**TSC4, TSC6,** and **TSC8**) were identified in the MD studies to form highly unstable complexes. More stable complexes (**TSC10, TSC2,** and **TSC9**) were linked in the in vitro study with higher IC_50_ values for the inhibition of VEGFR2. MM-PBSA analysis provided a similar pattern of SAR in terms of the stability of the ligand–VEGFR2 complex among the **TSC1-TSC10** series, with the major driving force in complex stability being comprised of van der Waals energy. A detailed decomposition of the energetic contribution of the AAs found within 5 Å of the ligand suggests that Lys868 is mainly responsible through electrostatic repulsions for the instability of the complexes due to the poor fitting of the distal residue in the HYDII area.

Given the provided in vitro and in silico data that explore the anti-angiogenic, anti-VEGFR2, and antiproliferative potential in agreement with the initial hypothesis of the **TSC1-TSC10** series, we strongly believe that our original work delivered an innovative structural design and SAR observations, which could provide a building block for novel and clinically relevant VEGFR2 inhibitors in the fight against cancer.

## Figures and Tables

**Table 1:** Experimental IC_50_ values (µM) after 48 h exposure of the novel **TSC1-TSC10** series and **sorafenib** on the evaluated cell line panel (Ea.Hy926, HaCaT, and A375) and the selectivity indexes (SIs). The VEGFR2 kinase inhibitory activity potential of the **TSC1-TSC10** series and **sorafenib**.

Comp.	Evaluated Cell Lines			Selectivity Index		VEGFR2 Kinase Inhibitory Activity	
	EA.hy926	HaCaT	A375	^1^SI_a_	^2^SI_b_	% of Inhibition (500 nM)	VEGFR2 IC_50_ (nM)
**TSC1**	49,9	≈100	>100	2.00	-	52.70	389.00
**TSC2**	>100	>100	>100	-	-	59.46	340.75
**TSC3**	26.51	28.63	>100	1.08	-	62.10	239.41
**TSC4**	14.47	15.94	14.82	1.10	1.08	40.49	-
**TSC5**	7.47	16.45	17.35	2.20	0.95	70.98	378.13
**TSC6**	16.49	23.02	61.04	1.40	0.38	0	-
**TSC7**	43.19	53.83	>100	1.25	-	21.09	-
**TSC8**	>100	>100	>100	-	-	0	-
**TSC9**	12.57	86.76	>100	6.90	-	70.46	378.68
**TSC10**	12.79	52.35	28.57	4.09	1.83	89.08	119.04
**Sorafenib**	10.15	12.87	5.26	1.27	2.45	99.87	8.99

The provided data are depicted as relative means ± standard deviations of three biological replicates. Data were expressed as relative values related to the NC (100%) with significant differences (*p* < 0.05); ^1^SI_a_ = IC_50_ HaCaT/IC_50_ EA.hy296; and ^2^SI_b_ = IC_50_ HaCaT/IC_50_ A375.

**Table 2:** MD parameters predicted for the **TSC1-TSC10** series and **sorafenib** in complex with the apoVEGFR2.

System Evaluated (Compound + apoVEGFR2)	RMSD-Protein (nm) ^1^	RMSF AA αCarbons (nm) ^2^	RMSF AA Side Chains (nm) ^3^	RMSD- Ligand (nm) ^4^	Rg-Protein (nm) ^5^	NoHB Protein–Ligand (no/ns) ^6^
**TSC1**	0.19	0.17	0.12	0.22	2.06	0.23
**TSC2**	0.17	0.15	0.10	0.14	2.05	0.33
**TSC3**	0.19	0.16	0.11	0.21	2.04	0.18
**TSC4**	0.29	0.17	0.12	0.42	2.09	0.07
**TSC5**	0.22	0.14	0.09	0.28	2.08	0.27
**TSC6**	0.25	0.18	0.13	0.38	2.09	0.24
**TSC7**	0.28	0.19	0.14	0.25	2.08	0.11
**TSC8**	0.23	0.17	0.12	0.33	2.08	0.37
**TSC9**	0.23	0.16	0.10	0.26	2.08	0.20
**TSC10**	0.22	0.17	0.11	0.24	2.06	0.44
**Sorafenib**	0.18	0.13	0.17	0.12	2.05	1.00
**apoVEGFR2** ^7^	0.19	0.11	0.16	-	2.05	-

^1^ Root mean square deviation of the backbone of the apoVEGFR2 receptor; ^2,3^ root mean square fluctuation (RMSF) of the side chains of the AAs and the α-carbons from AAs; ^4^ root mean square deviation of the heavy atoms of the ligands; ^5^ radius of gyration of the apoVEGFR2 receptor; ^6^ average number of H-bonds (NoHB) generated in the ligand–protein complex during the 100 ns simulations; and ^7^ baseline parameters for the protein (VEGFR2) under similar experimental conditions.

**Table 3:** The decomposition in types of free binding energy for the **TSC1-TSC10** series and **sorafenib** in complex with VEGFR2 for the last 25 ns of the MD simulations (kcal/mol).

Compound	ΔG (±SD)	Van der Waals (±SD)	Electrostatic (±SD)	Solvation (±SD)
**TSC1**	−70.97 ± 5.31	−54.98 ± 3.14	−46.82 ± 4.11	30.83 ± 3.78
**TSC2**	−74.24 ± 3.88	−69.68 ± 3.02	−29.58 ± 4.19	25.03 ± 2.51
**TSC3**	−68.70 ± 5.86	−62.60 ± 3.31	−36.63 ± 4.99	30.52 ± 6.29
**TSC4**	−33.23 ± 8.64	−57.58 ± 3.67	−89.73 ± 30.80	114.08 ± 22.0
**TSC5**	−40.79 ± 4.92	−50.67 ± 3.30	−123.28 ± 10.73	133.16 ± 8.90
**TSC6**	−35.61 ± 10.71	−55.14 ± 3.59	−97.16 ± 19.49	116.69 ± 14.33
**TSC7**	−43.71 ± 5.60	−57.34 ± 2.52	−8.94 ± 3.78	22.57 ± 4.07
**TSC8**	−44.39 ± 5.04	−57.81 ± 2.96	−117.23 ± 11.78	130.64 ± 8.82
**TSC9**	−72.41 ± 3.51	−58.85 ± 3.06	−39.43 ± 3.80	25.88 ± 2.35
**TSC10**	−62.18 ± 4.11	−65.93 ± 2.78	−12.24 ± 4.58	15.99 ± 3.74
**Sorafenib**	−69.39 ± 3.63	−59.19 ± 2.81	−39.67 ± 3.76	29.47 ± 2.65

## Data Availability

The original contributions presented in this study are included in the article and the Supplementary Materials. Further inquiries can be directed to the corresponding author.

## Supplementary Materials

The following supporting information can be downloaded at https://www.mdpi.com/article/10.3390/pharmaceutics17020260/s1: Figures S1–S10. The IR spectrum of TSC1-TSC10 derivatives; Figures S11–S20. The MS spectra of TSC1-TSC10 derivatives; Figures S21–S30. The *^1^H-NMR* spectrum of TSC1-TSC10 derivatives; Figures S31–S40. The *^13^C-NMR* spectrum of TSC1-TSC10 derivatives; Figure S41. Cytotoxic effects of TSC1-TSC10 derivatives after 48 h exposure to EA.hy926, HaCaT, and A375 cells; Figure S42. The impact on angiogenesis in the CAM assay following treatment with different concentrations (1 μM, 5 μM, and 10 μM) of sorafenib, TSC5, TSC9, TSC10, and DMSO 0.2% from day 1 to day 5 of the experiment.; Figure S43. Bioavailability radar for the TSC1-TSC10 series and sorafenib by SwissADME; Figures S44–S47. Molecular dynamics parameter (graphical representation) evolution during the 100 ns simulations for TSC1 and TSC3-TSC9; Figure S48. Free energy decomposition for all the AAs in the 5Å proximity of the ligand (TSC1, TSC3-TSC8) during the last 2500 frames of the MD simulation; Table S1. In silico physicochemical and drug-likeness predictions of the TSC1-TSC10 series and sorafenib by SwissADME; Table S2. In silico ADME predictions of the TSC1-TSC10 series and sorafenib by SwissADME and pkCSM; Table S3. In silico toxicity predictions of the TSC1-TSC10 series and sorafenib by pkCSM; Table S4. The 2D depictions of the interactions between the studied TSC1-TSC10 series and the active site of VEGFR2; Table S5. The DFT parameters of the TSC1-TSC10 series and sorafenib; Table S6. HOMO-LUMO orbital distribution and MEP for the TSC1-TSC9 series; Table S7. The depiction of the pose of the ligands (TSC1-TSC10) at the beginning of the simulation and at the end of the simulation within the active site of VEGFR2; Table S8. H-bond decomposition in the complexes of TSC1-TSC10 and sorafenib with VEGFR2 during the 100 ns simulations and the encounter frequency (%); Table S9. H-bond decomposition in quarters generated in the complexes of TSC2, TSC10, and sorafenib with VEGFR2 during the 100 ns simulations and the encounter frequency (%) for the most relevant AAs.
