# Supplementary material for: Rational Design and Synthesis of a Novel Series of Thiosemicarbazone-Containing Quinazoline Derivatives as Potential VEGFR2 Inhibitors
Source: Pharmaceutics. 2025 Feb 15;17(2):260. doi: 10.3390/pharmaceutics17020260 (PMC11860020; doi:10.3390/pharmaceutics17020260)
Supplement: Supplementary file 1 [file pharmaceutics-17-00260-s001.zip › pharmaceutics-3457609-supplementary.pdf]

pharmaceutics

MDPI

*Article*

# Supplementary Material

## 1. Chemistry

### *1.1 IR spectrum*

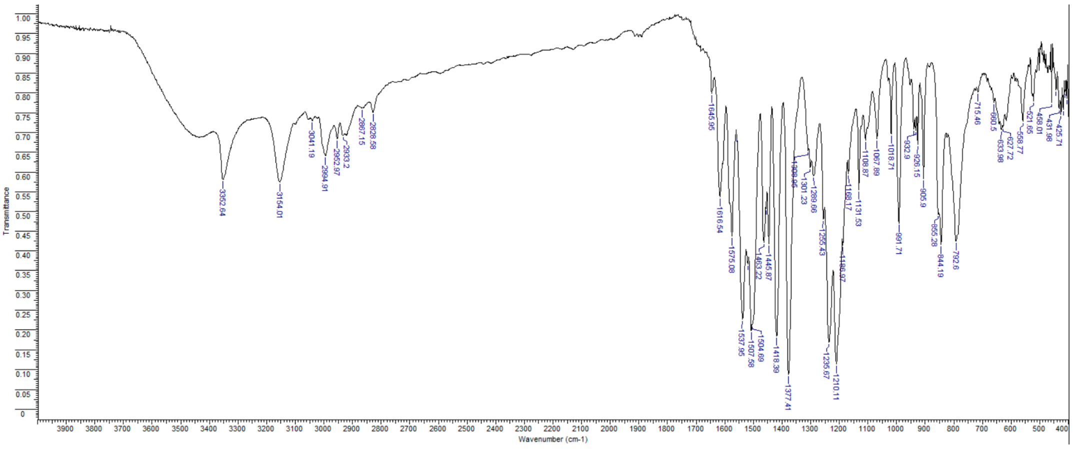


**Figure S1.** The IR spectrum for the compound TSC1

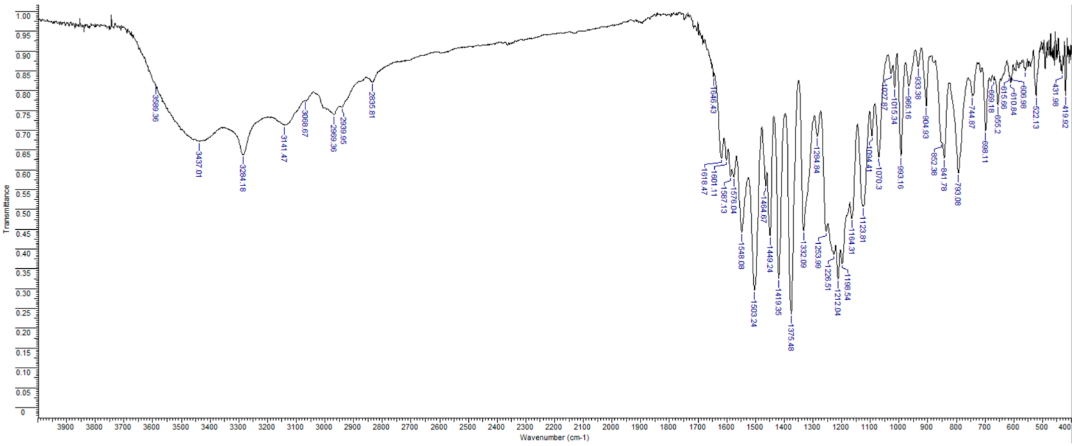


**Figure S2.** The IR spectrum for the compound TSC2

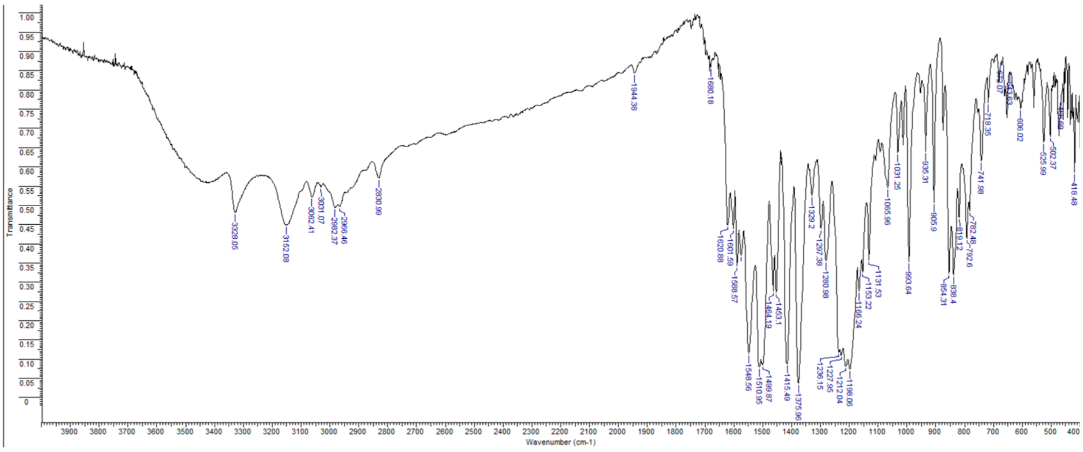

**Figure S3.** The IR spectrum for the compound TSC3

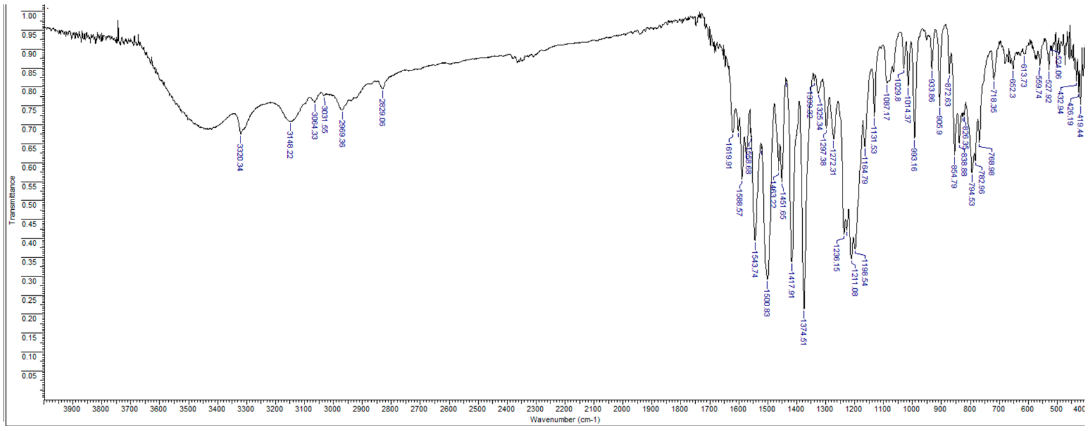


**Figure S4.** The IR spectrum for the compound TSC4

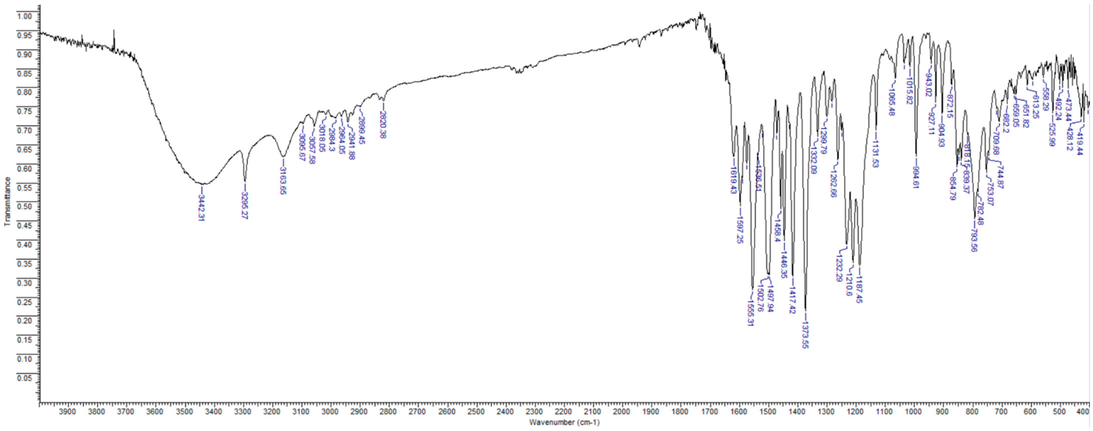


**Figure S5.** The IR spectrum for the compound TSC5

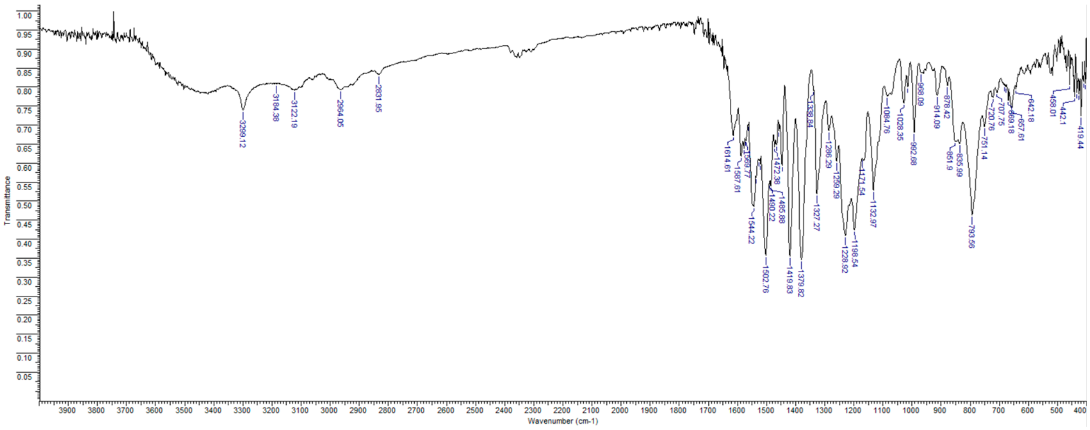


**Figure S6.** The IR spectrum for the compound TSC6

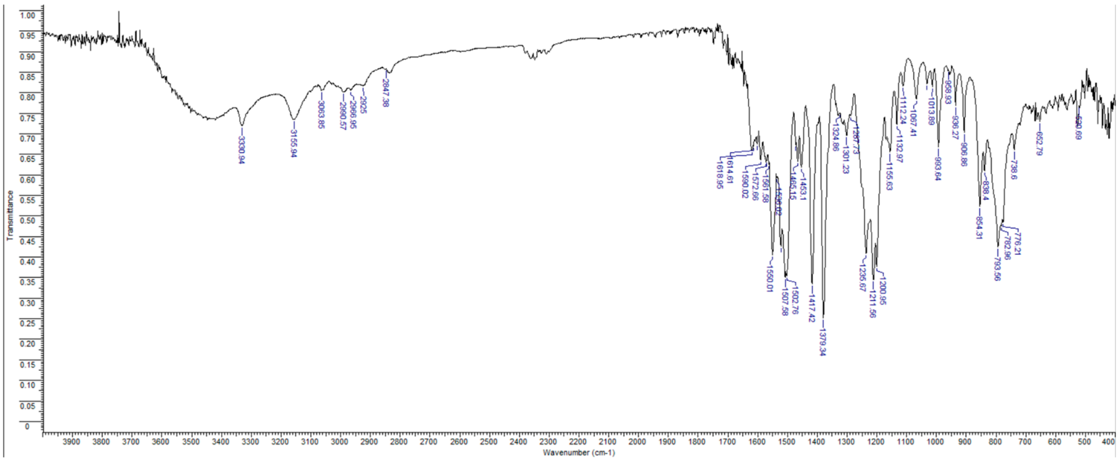


**Figure S7.** The IR spectrum for the compound TSC7

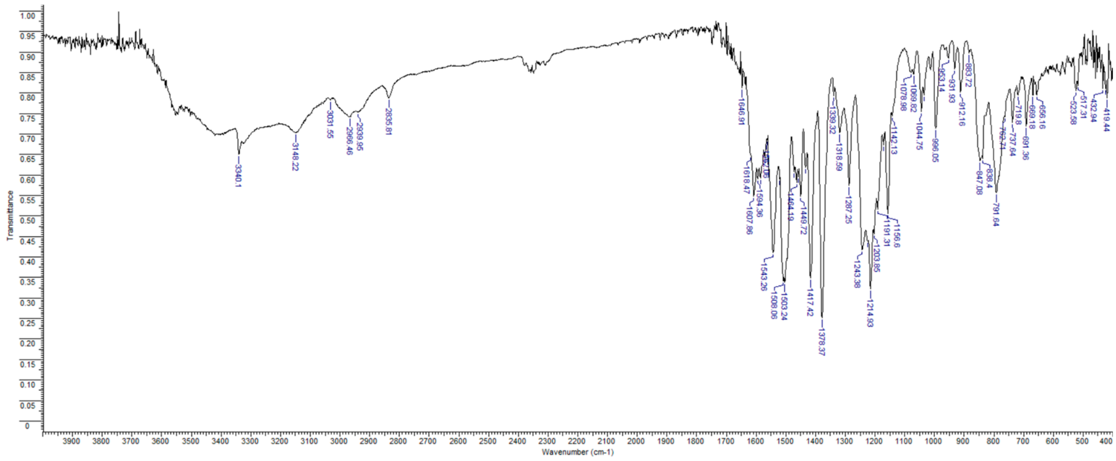


**Figure S8.** The IR spectrum for the compound TSC8

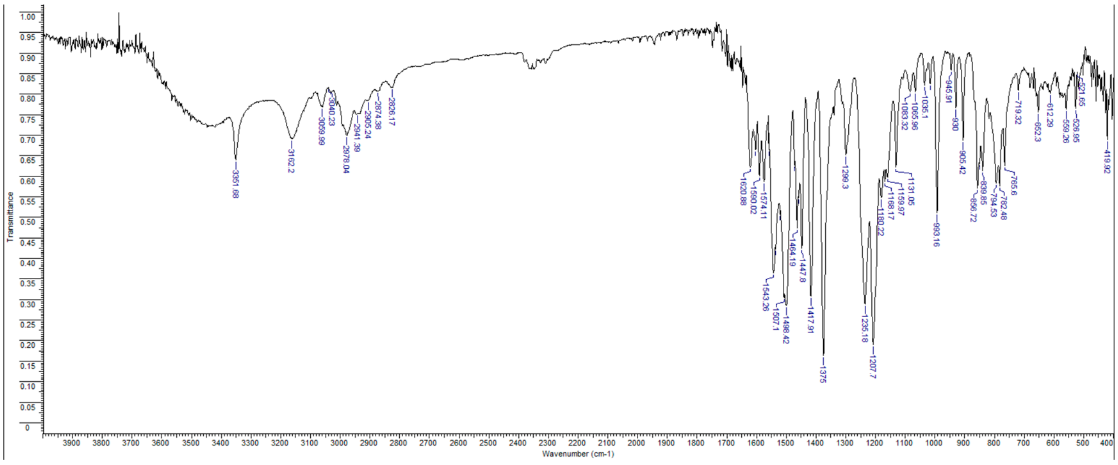


**Figure S9.** The IR spectrum for the compound TSC9

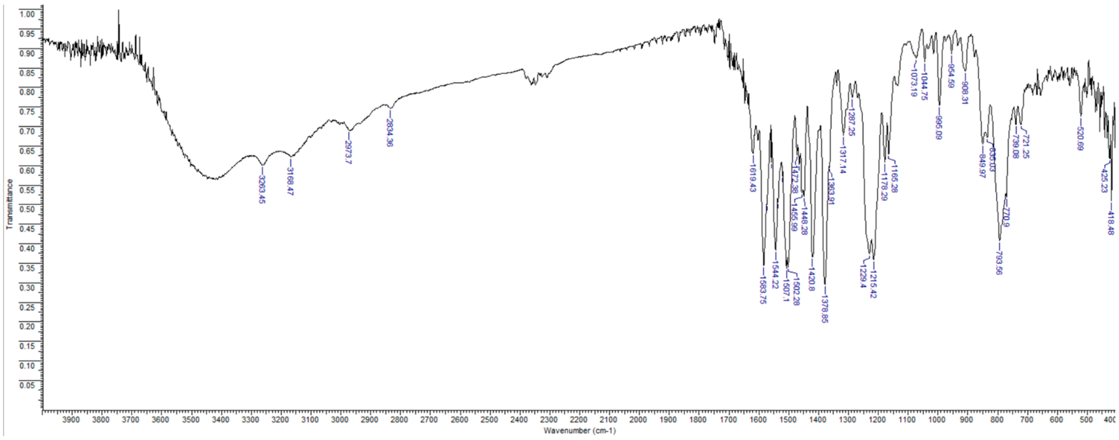


**Figure S10.** The IR spectrum for the compound TSC10

## *1.2. The MS spectra*

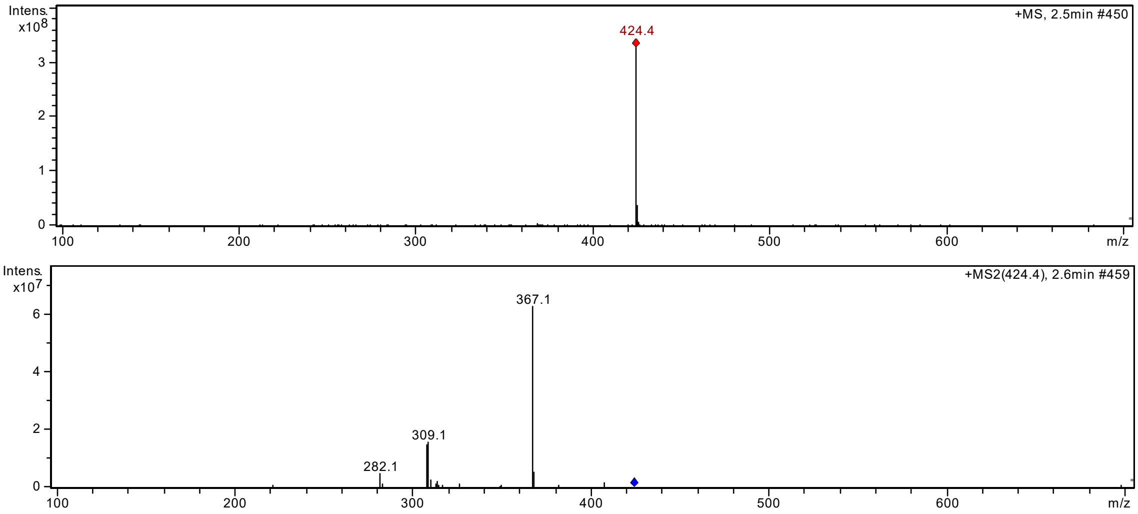


**Figure S11.** The MS spectra for the compound TSC1

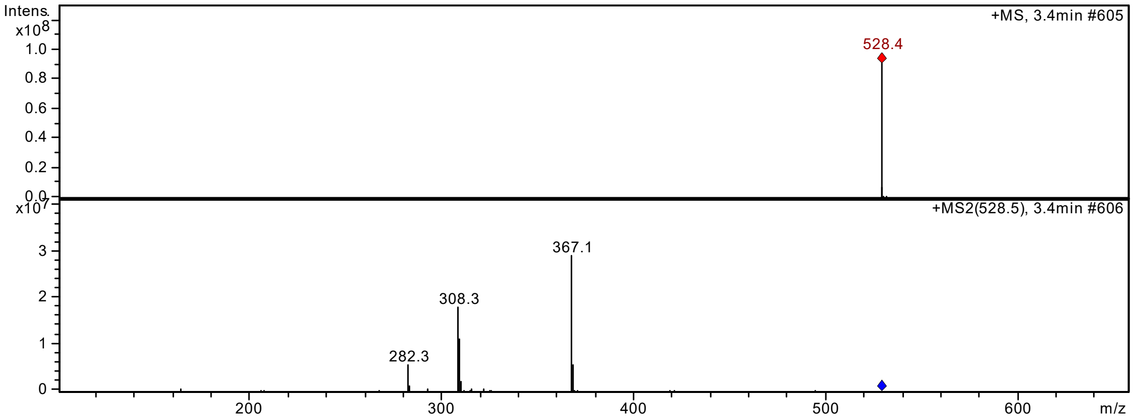


**Figure S12.** The MS spectra for the compound TSC2

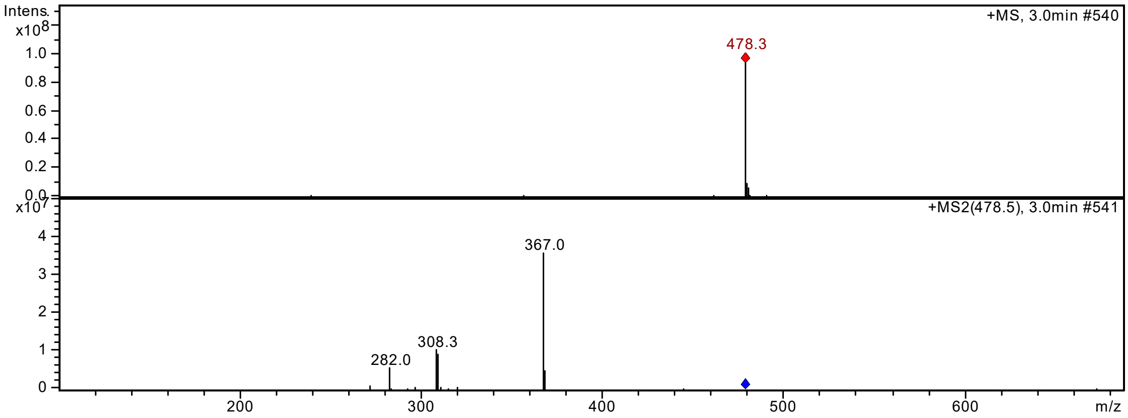


**Figure S13.** The MS spectra for the compound TSC3

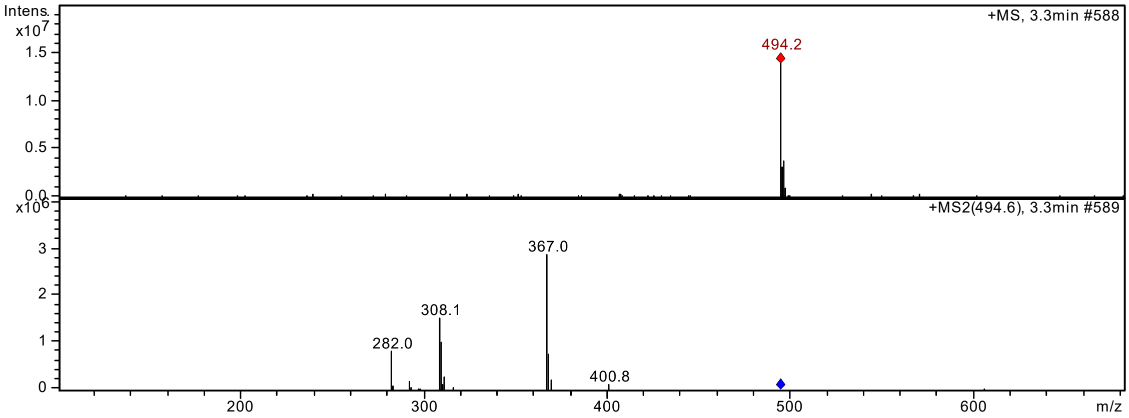


**Figure S14.** The MS spectra for the compound TSC4

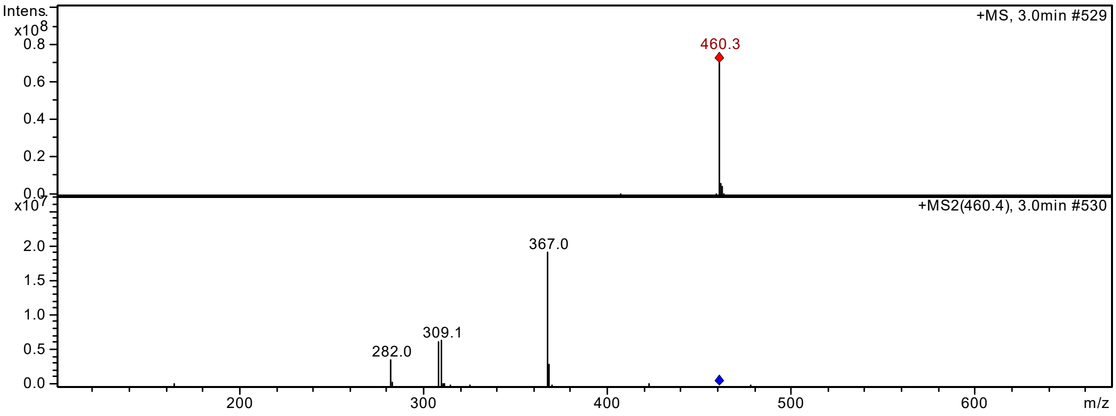


**Figure S15.** The MS spectra for the compound TSC5

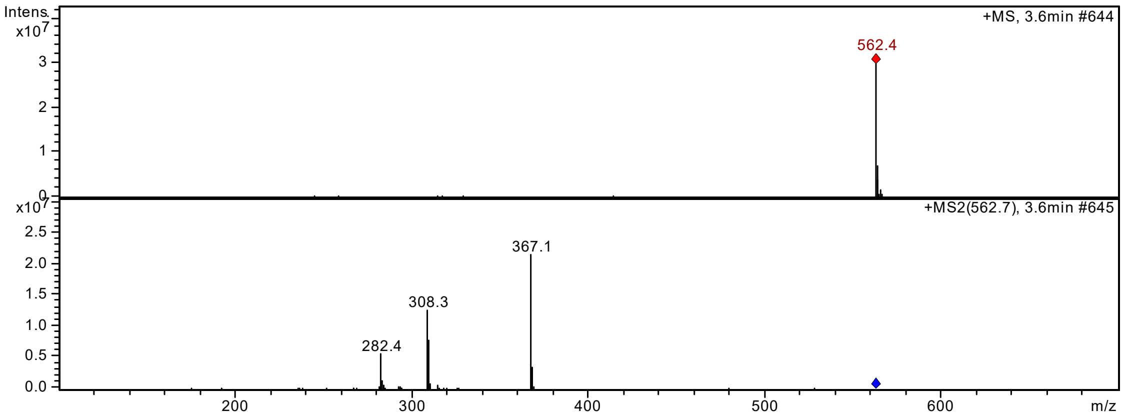


**Figure S16.** The MS spectra for the compound TSC6

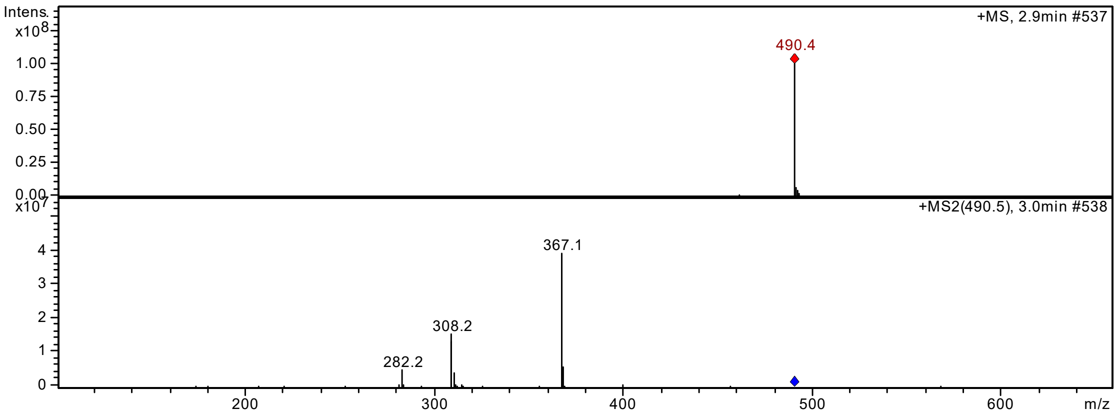


**Figure S17.** The MS spectra for the compound TSC7

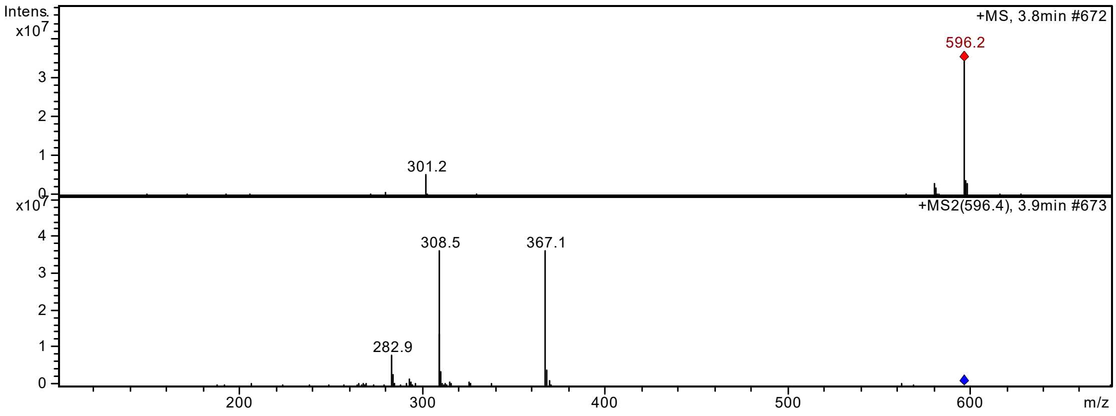


**Figure S18.** The MS spectra for the compound TSC8

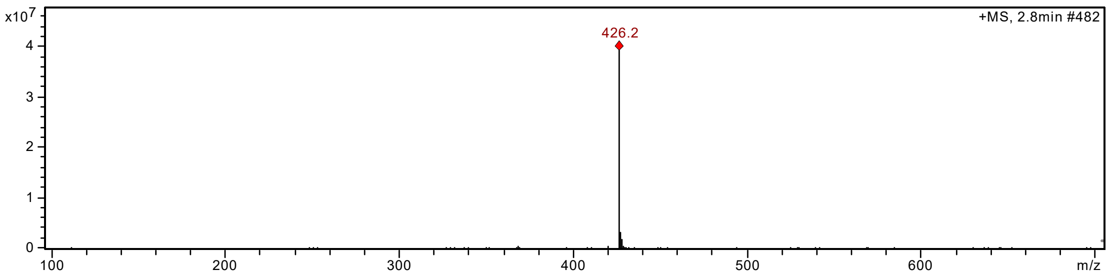

**Figure S19.** The MS spectra for the compound TSC9

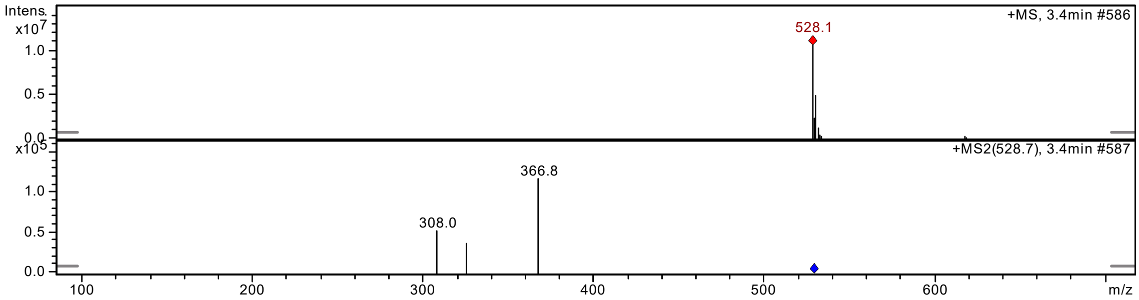


**Figure S20.** The MS spectra for the compound TSC10

## 1.3. The $^{1}$H-NMR spectrum

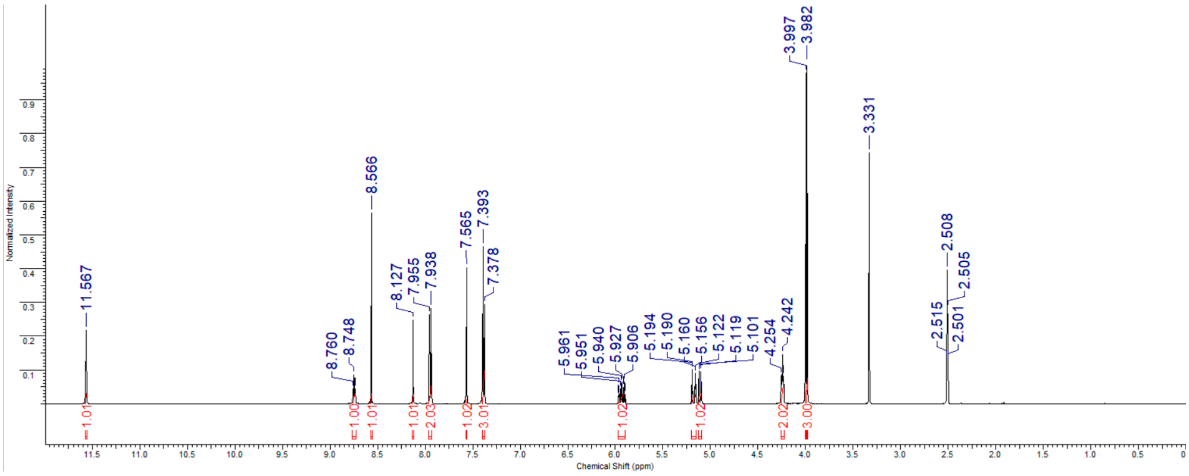


**Figure S21.** The *1H-NMR* spectrum for the compound TSC1

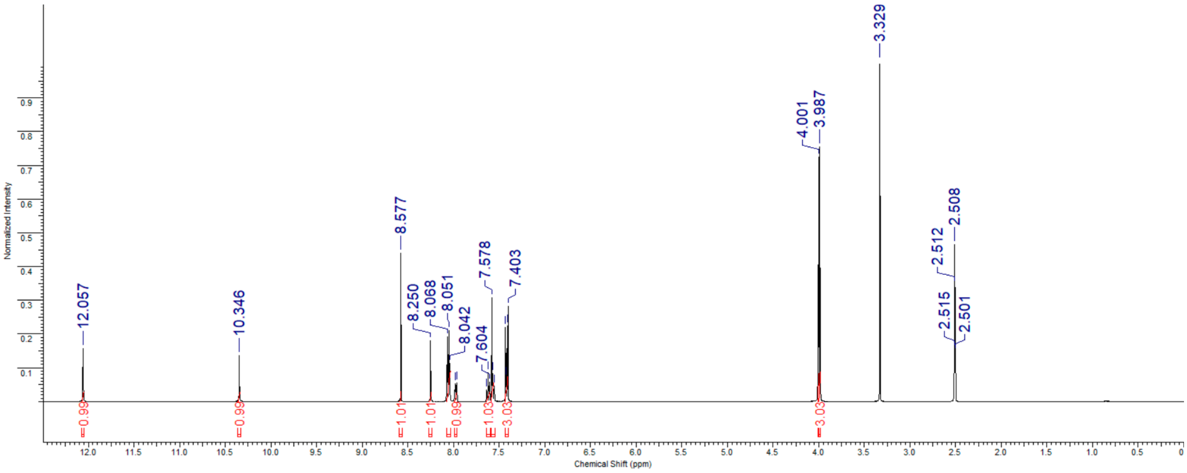


**Figure S22.** The *1H-NMR* spectrum for the compound TSC2

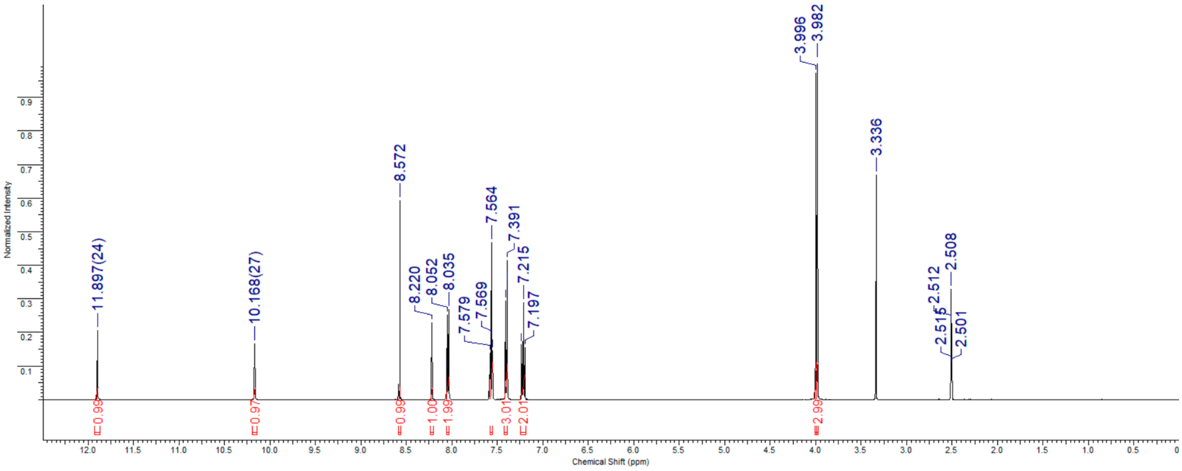


**Figure S23.** The *1H-NMR* spectrum for the compound TSC3

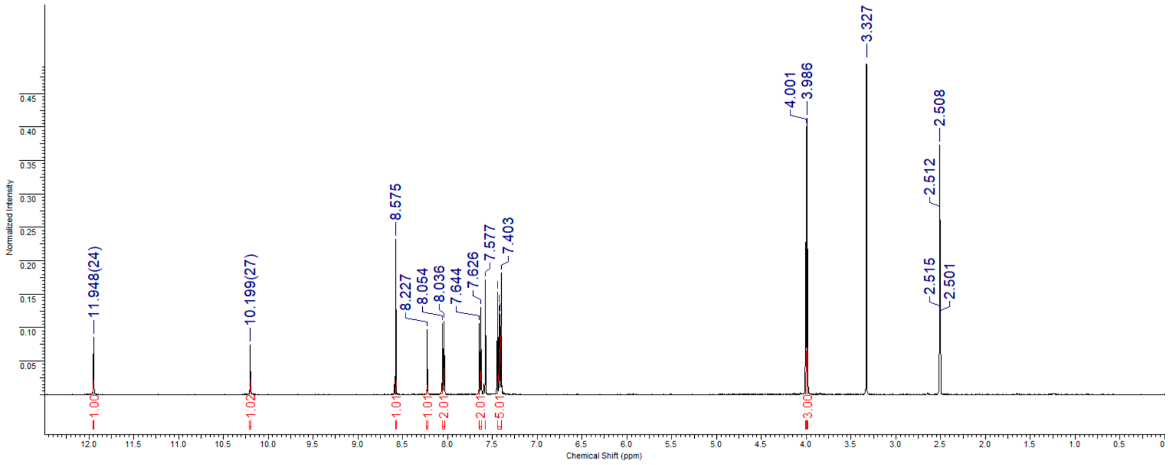


**Figure S24.** The *1H-NMR* spectrum for the compound TSC4

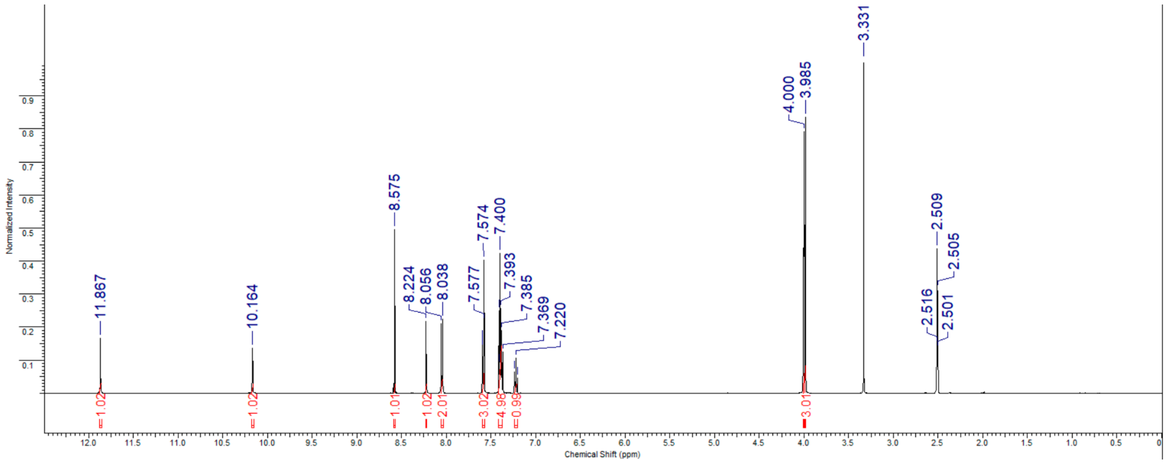


**Figure S25.** The *1H-NMR* spectrum for the compound TSC5

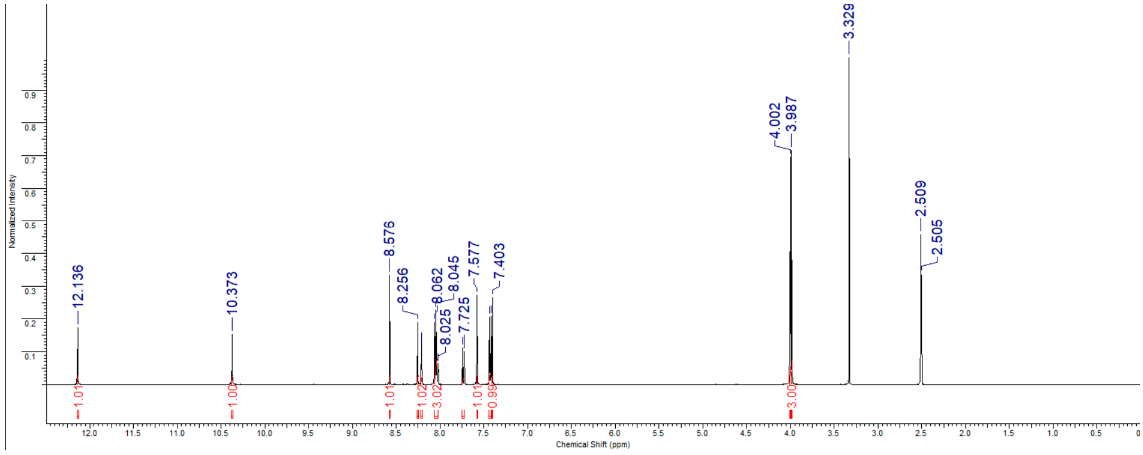


**Figure S26.** The *1H-NMR* spectrum for the compound TSC6

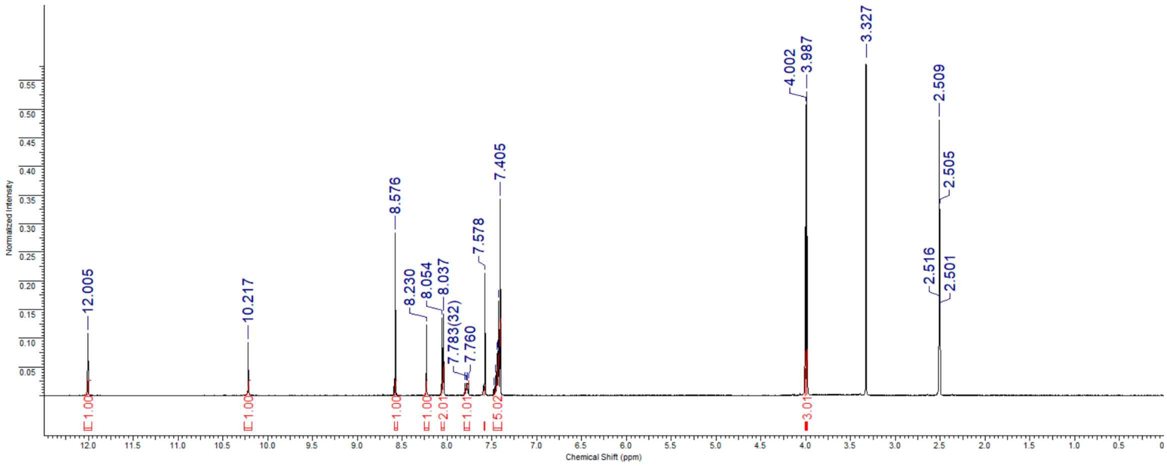


**Figure S27.** The *1H-NMR* spectrum for the compound TSC7

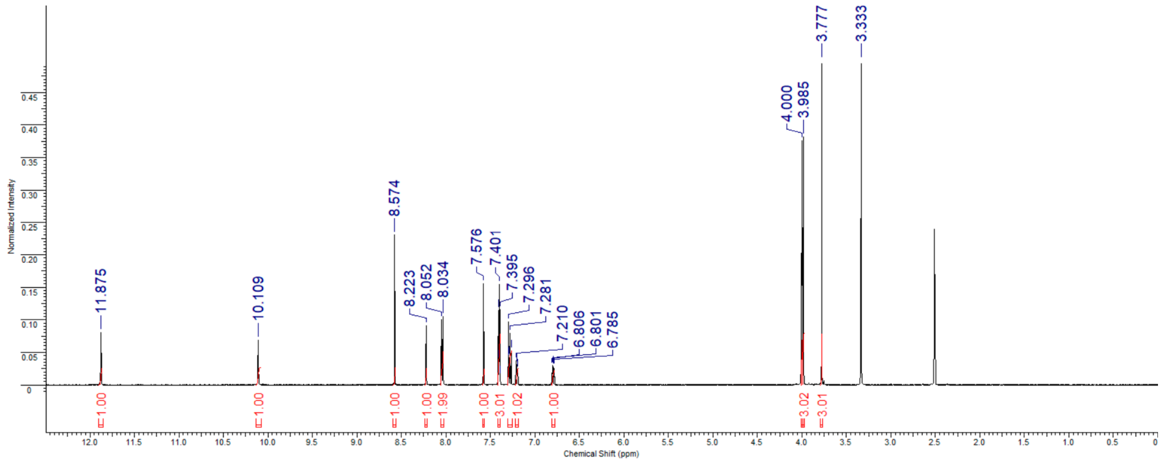


**Figure S28.** The *1H-NMR* spectrum for the compound TSC8

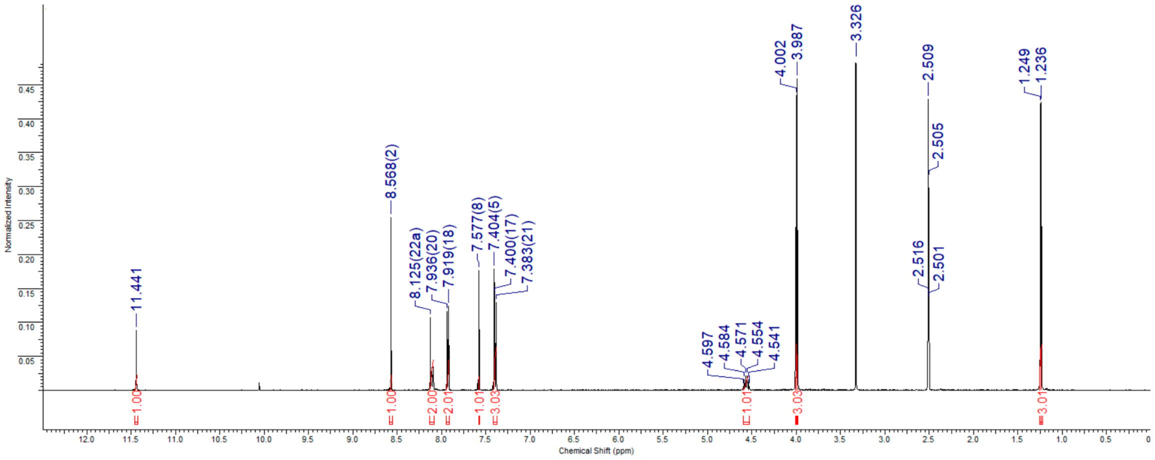


**Figure S29.** The *1H-NMR* spectrum for the compound TSC9

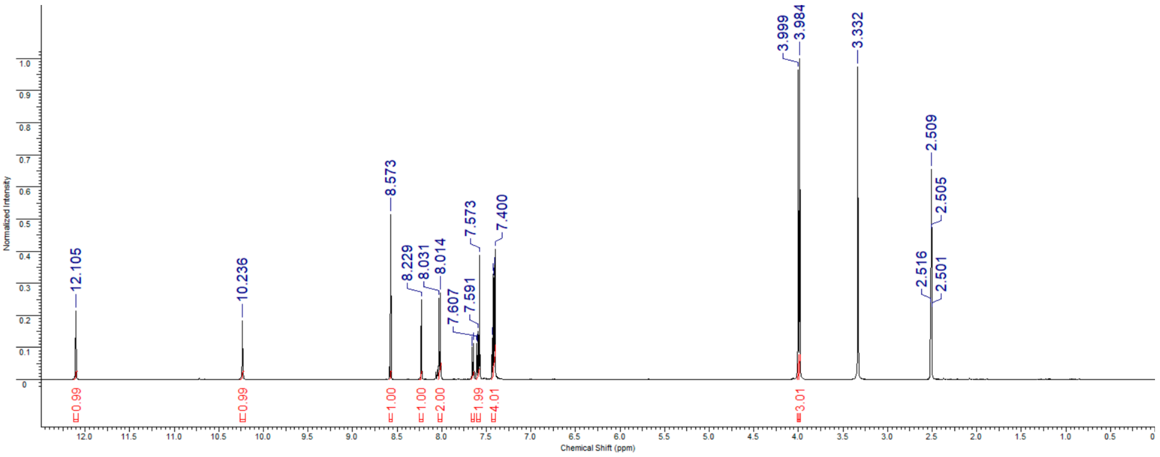


**Figure S30.** The *1H-NMR* spectrum for the compound TSC10

## *1.4. The* [13]*C-NMR spectra*

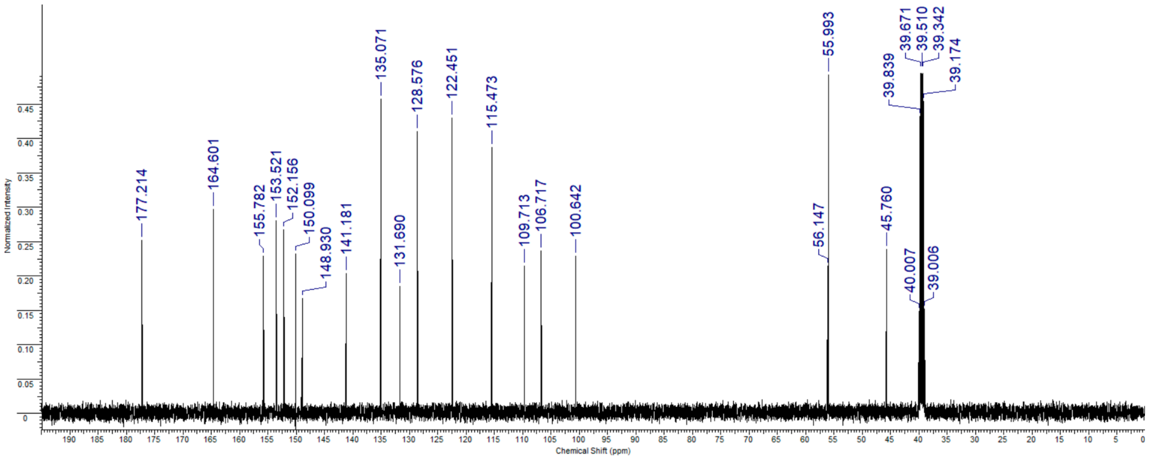


**Figure S31.** The [13]*C-NMR* spectrum for the compound TSC1

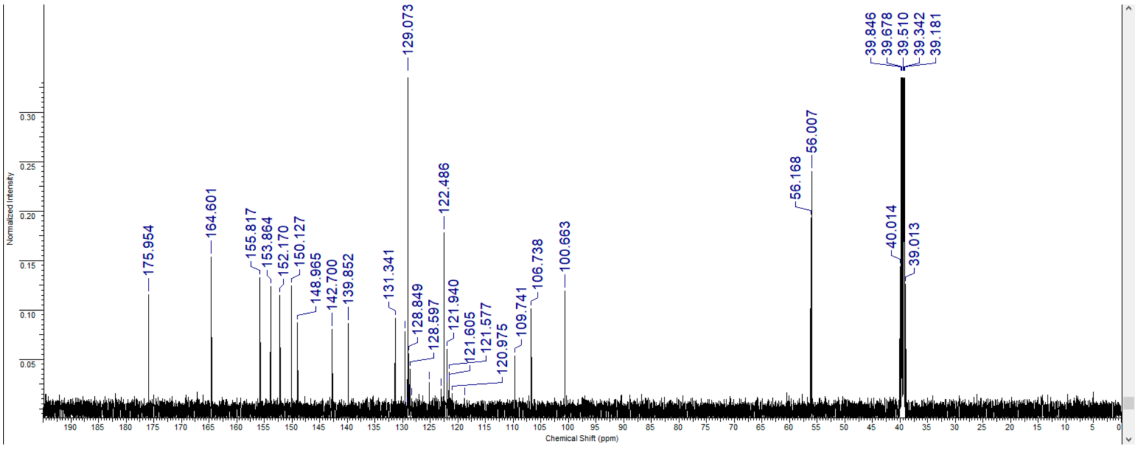


**Figure S32.** The [13]*C-NMR* spectrum for the compound TSC2

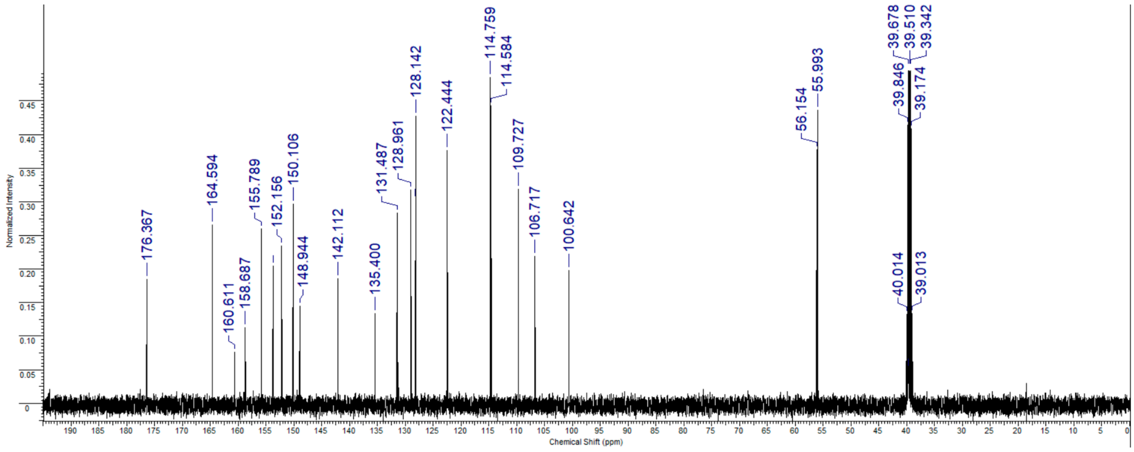


**Figure S33.** The [13]*C-NMR* spectrum for the compound TSC3

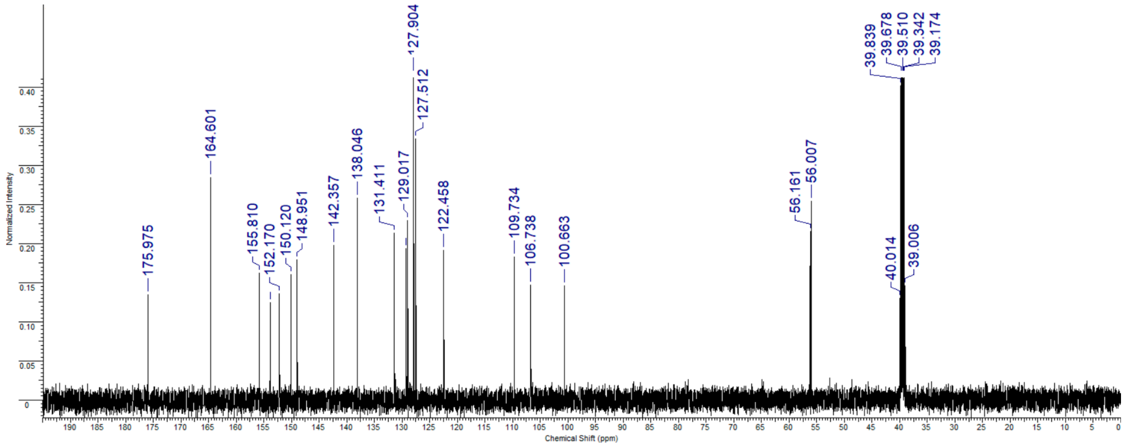


**Figure S34.** The [13]*C-NMR* spectrum for the compound TSC4

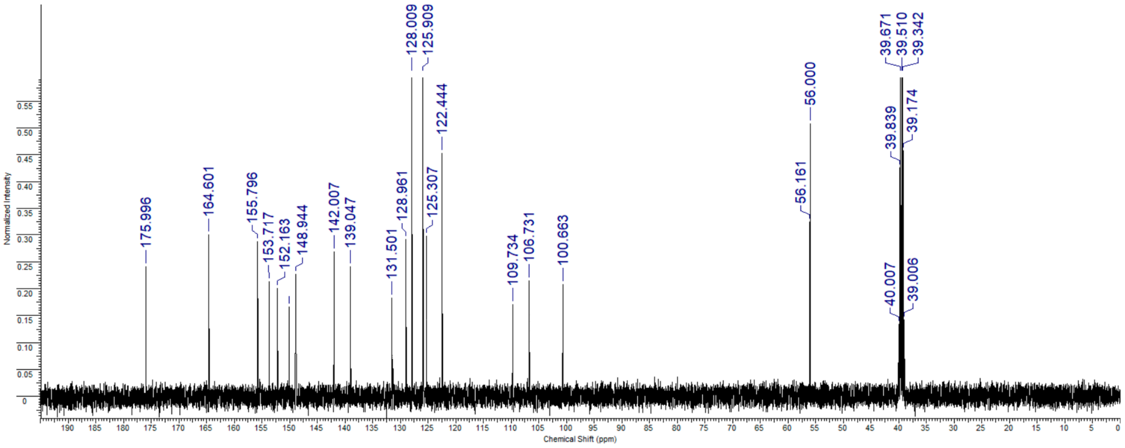


**Figure S35.** The *13C-NMR* spectrum for the compound TSC5

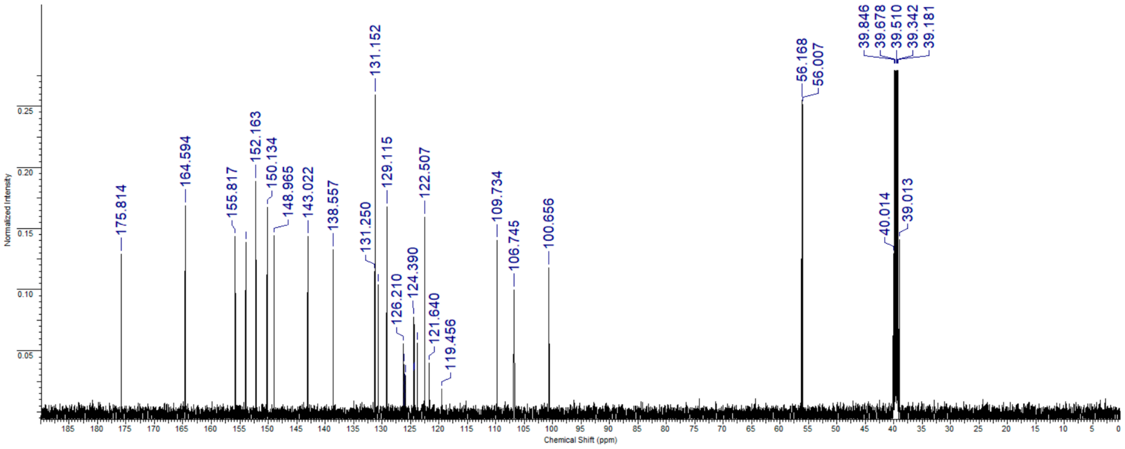


**Figure S36.** The *13C-NMR* spectrum for the compound TSC6

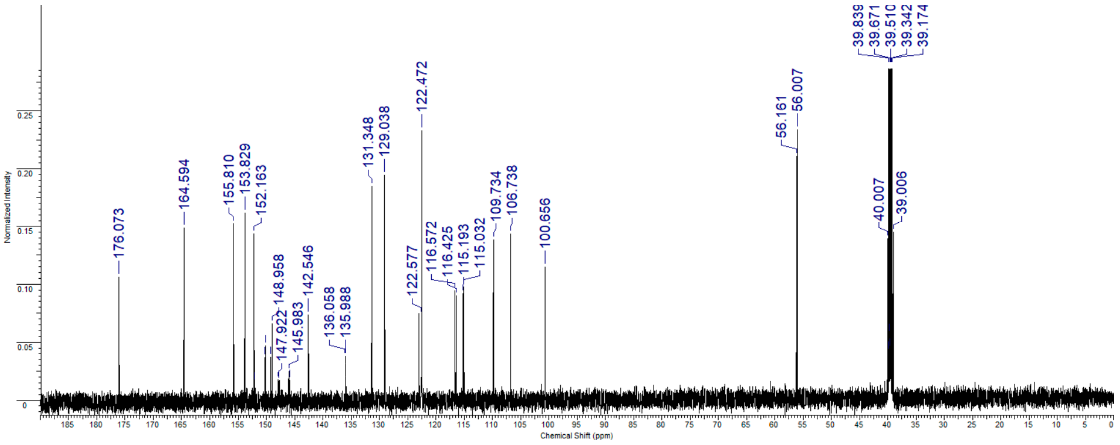


**Figure S37.** The *13C-NMR* spectrum for the compound TSC7

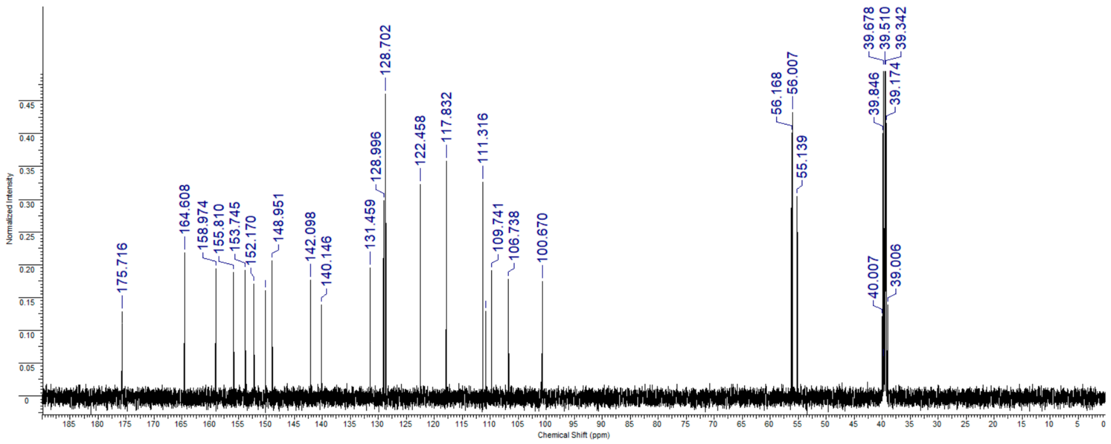


**Figure S38.** The [13]*C-NMR* spectrum for the compound TSC8

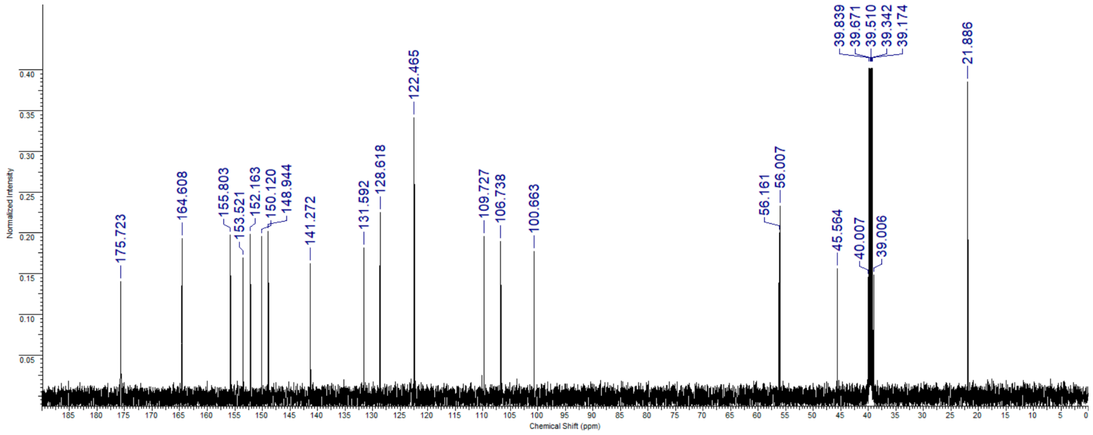


**Figure S39.** The [13]*C-NMR* spectrum for the compound TSC9

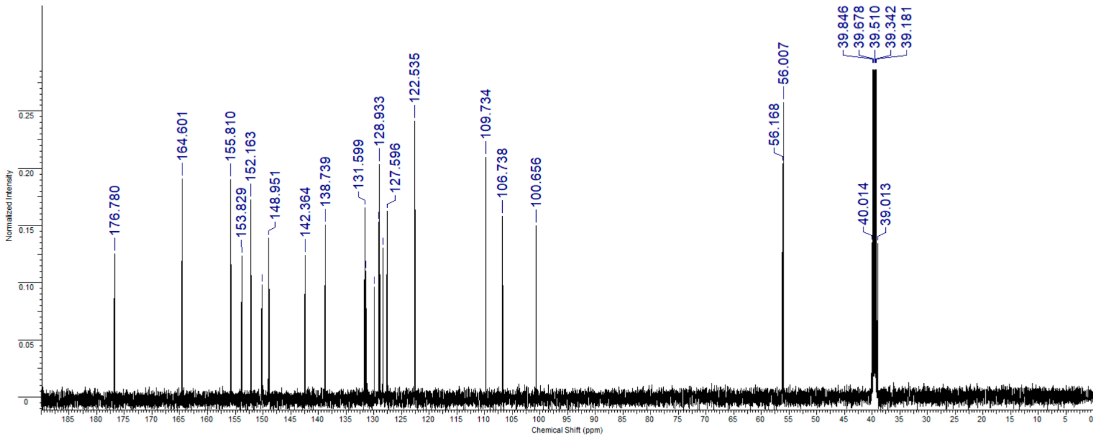


**Figure S40.** The [13]*C-NMR* spectrum for the compound TSC10

## 2. *In vitro* cytotoxicity evaluation

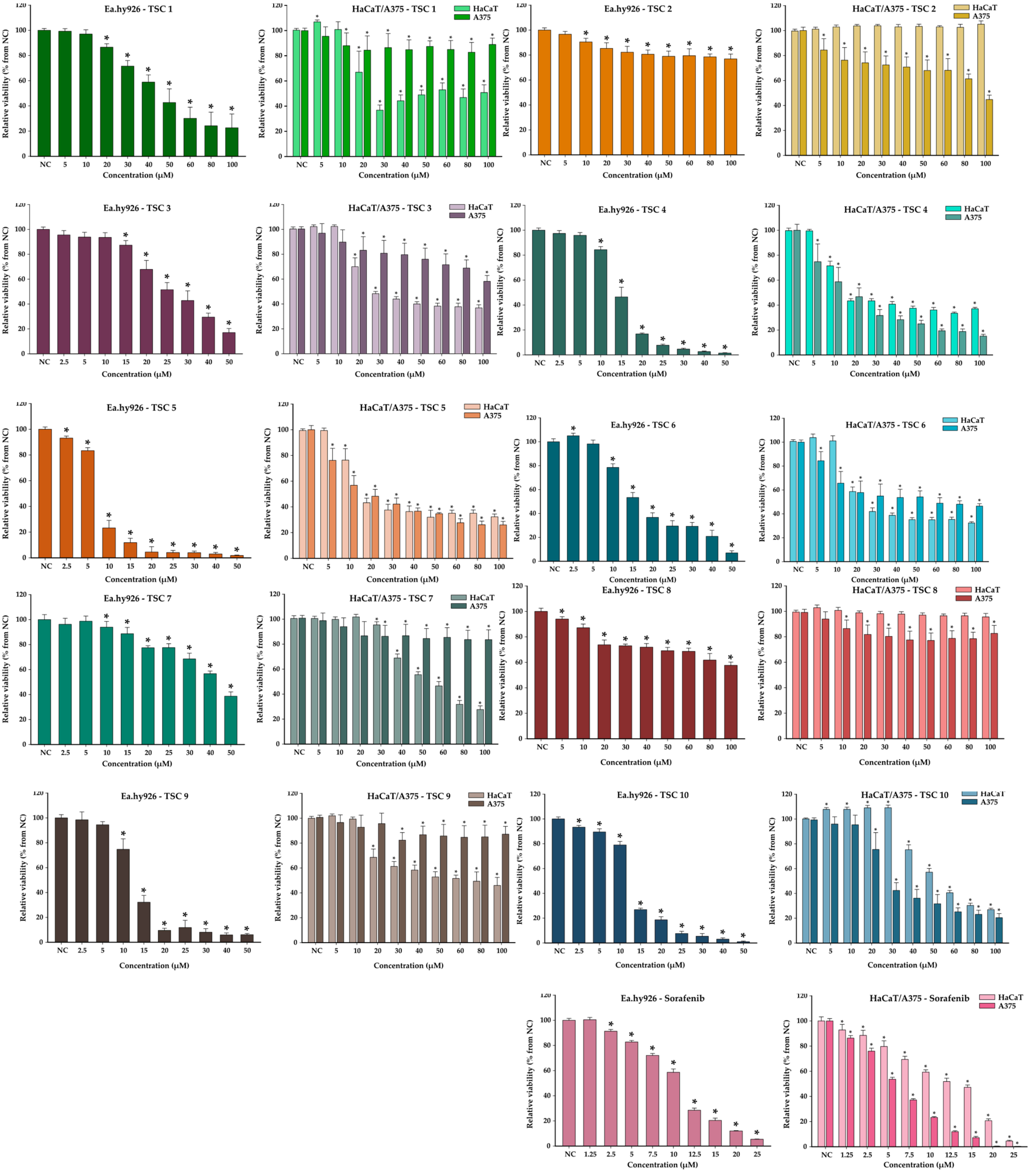


**Figure S41.** Cytotoxic effect of **TSC1-TSC10** after a 48h exposure of EA.hy926, HaCaT, and A375 cells. The provided data are depicted as relative means ± standard deviations of three biological replicates. Data were expressed as relative values related to the negative control (NC)(100%). Asterisks (*) marks significant differences ($p < 0.05$) compared to NC.

## 3. Chorioallantoic egg membrane (CAM) assay

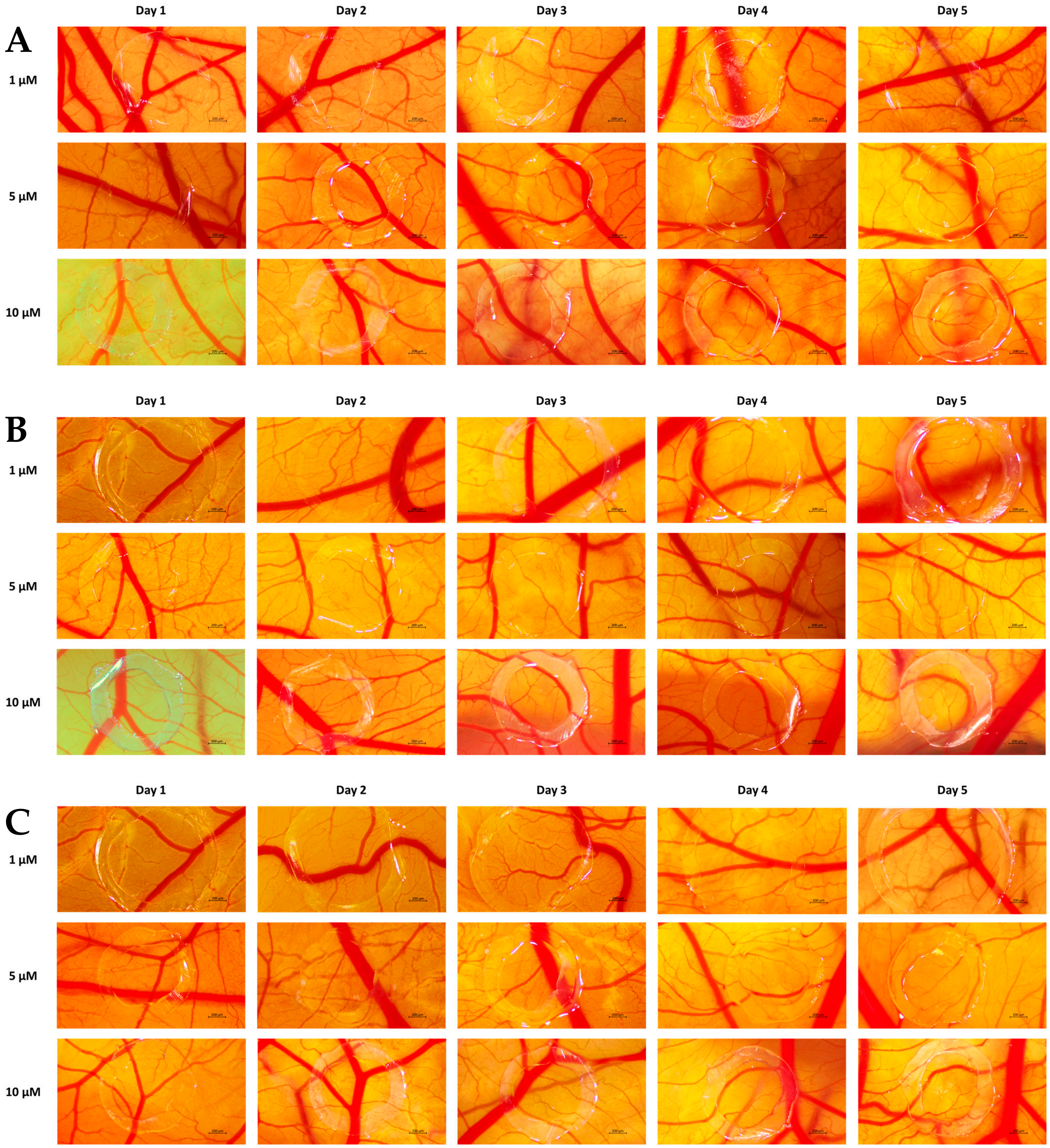

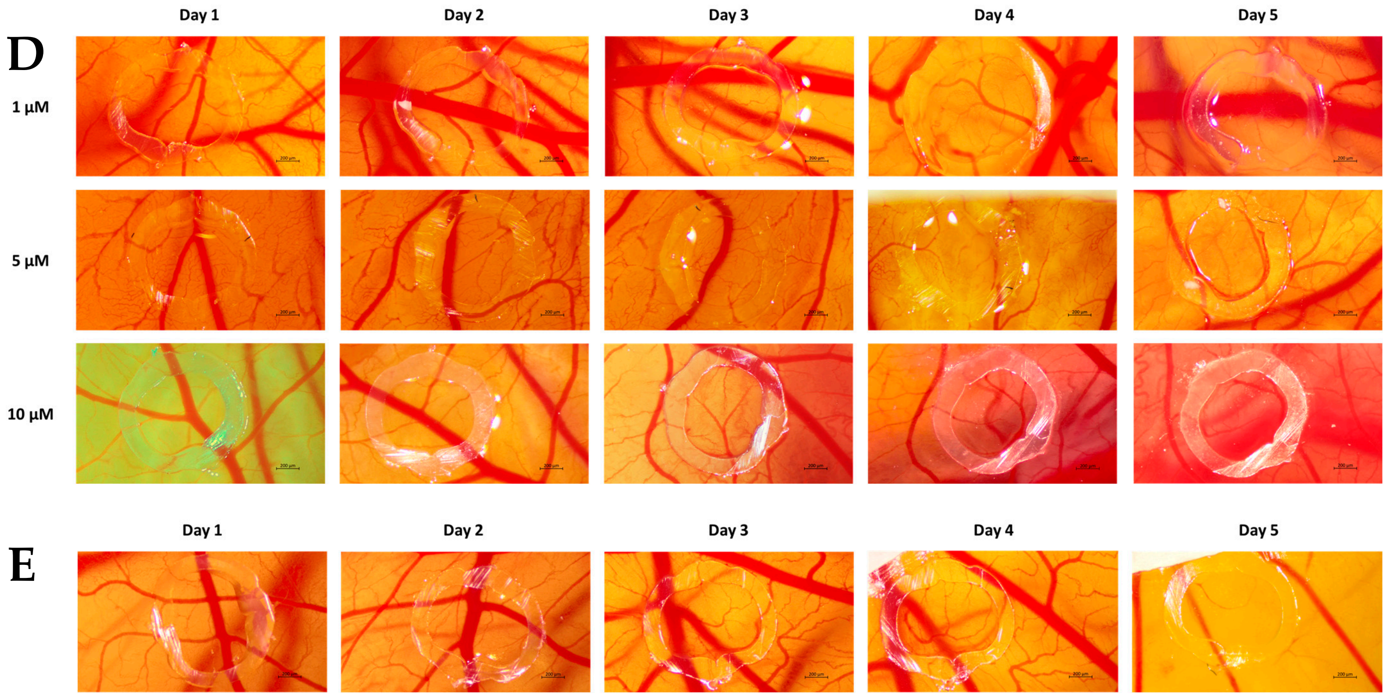


**Figure S42**. The impact on angiogenesis in the CAM assay following the treatment with different concentrations (1 µM, 5 µM, 10 µM) of (A) **sorafenib**, (B) **TSC5,** (C) **TSC9**, (D) **TSC10**, (E) **DMSO 0.2%** from day 1 to day 5 of the experiment.

## 4. ADMETox and drug-likeness profile

**Table S1**. *In silico* Physicochemical and druglikeness predictions of the **TSC1-TSC10** series & **sorafenib** by SwissADME

| | **TSC1** | **TSC2** | **TSC3** | **TSC4** | **TSC5** | **Sorafenib** |
|---|---|---|---|---|---|---|
| | **TSC6** | **TSC7** | **TSC8** | **TSC9** | **TSC10** | |
| Physico-Chemical properties | | | | | | |
| Molecular Weight (g/mol) | 423.49 | 527.52 | 477.51 | 493.97 | 459.52 | 464.82 |
| | 561.96 | 495.50 | 489.55 | 425.50 | 528.41 | |
| Number Heavy Atoms | 30 | 37 | 34 | 34 | 33 | 32 |
| | 38 | 35 | 35 | 30 | 35 | |
| Number Ar. Heavy Atoms | 16 | 22 | 22 | 22 | 22 | 18 |
| | 22 | 22 | 22 | 16 | 22 | |
| Fraction $C_{sp3}$ | 0.14 | 0.12 | 0.08 | 0.08 | 0.08 | 0.10 |
| | 0.12 | 0.08 | 0.12 | 0.24 | 0.08 | |
| Num. Rotatable Bonds | 10 | 10 | 9 | 9 | 9 | 9 |
| | 10 | 9 | 10 | 9 | 9 | |
| Num. H-bonds Acceptors | 6 | 9 | 7 | 6 | 6 | 7 |
| | 9 | 8 | 7 | 6 | 6 | |
| Num. H-bonds Donnors | 2 | 2 | 2 | 2 | 2 | 3 |
| | 2 | 2 | 2 | 2 | 2 | |

| | | | | | | |
|---|---|---|---|---|---|---|
| [1]TPSA (Å$^2$) | 121.98<br>121.98 | 121.98<br>121.98 | 121.98<br>131.21 | 121.98<br>121.98 | 121.98<br>121.98 | 92.35 |
| Lipophilicity | | | | | | |
| Log $P_{o/w}$ (XLOGP3) | 3.67<br>6.09 | 5.47<br>4.78 | 4.68<br>4.55 | 5.21<br>3.82 | 4.58<br>5.84 | 4.07 |
| Log $P_{o/w}$ (MLOGP) | 1.95<br>3.71 | 3.24<br>3.47 | 3.10<br>2.15 | 3.20<br>2.02 | 2.72<br>3.67 | 2.91 |
| Consensus Log $P_{o/w}$ | 3.43<br>5.42 | 5.11<br>4.72 | 4.32<br>4.12 | 4.50<br>3.56 | 4.17<br>5.20 | 4.10 |
| Water Solubility | | | | | | |
| Log S (ESOL) | -4.51<br>-6.93 | -6.34<br>-5.79 | -5.63<br>-5.55 | -6.07<br>-4.69 | -5.47<br>-6.67 | -5.71 |
| Solubility (mg/ml) | 1.30e-02<br>6.61e-05 | 2.43e-04<br>7.95e-04 | 1.11e-03<br>1.39e-03 | 4.21e-04<br>8.78e-03 | 1.54e-03<br>1.14e-04 | 8.98e-04 |
| [2]Class | Moderately<br>Poorly | Poorly<br>Moderately | Moderately<br>Moderately | Poorly<br>Moderately | Moderately<br>Poorly | Moderately |
| Drug-likeness | | | | | | |
| Lipinski Violations[a] | 0<br>1 | 1<br>0 | 0<br>0 | 0<br>0 | 0<br>1 | 0 |
| Veber Violations[b] | 0<br>0 | 0<br>0 | 0<br>0 | 0<br>0 | 0<br>0 | 0 |

[1]Topological polar surface area; [2]Log S scale (insoluble < -10 < poorly < -6 < moderately <-4 <soluble < -2 < very < 0 < highly); [a]Lipinski rules: (1) MW < 500 g/mol, (2) MLOGP < 4.15, (3) Numb. Of H-Bond acceptors < 10, (4) Nb. of H-Bond Acceptors < 5; [b]Veber Filter rules: (1) Rotatable bonds < 10, (2) TPSA < 140 Å$^2$;

**Table S2.** *In silico* ADME predictions of the **TSC1-TSC10** series & **sorafenib** by SwissADME and pkCSM.

| | **TSC1**<br>**TSC6** | **TSC2**<br>**TSC7** | **TSC3**<br>**TSC8** | **TSC4**<br>**TSC9** | **TSC5**<br>**TSC10** | **Sorafenib** |
|---|---|---|---|---|---|---|
| Absorbtion | | | | | | |
| Caco2[1] permeability (Log Papp in $10^{-6}$ cm/s)[a] | 1132<br>1055 | 0.971<br>1015 | 1008<br>1048 | 1043<br>1079 | 1094<br>1019 | 0.302 |
| GI absorbtion[b] | High<br>Low | Low<br>Low | Low<br>Low | Low<br>Low | High<br>Low | Low |
| P-glycoprotein substrate[b] | No<br>No | No<br>No | No<br>No | No<br>No | No<br>No | No |
| Distribution[a] | | | | | | |
| VDss[2] (human) (LogL/kg) | -0.074<br>-0.04 | -0.076<br>-0.219 | -0.172<br>-0.427 | -0.08<br>-0.089 | -0.135<br>0.035 | -0.107 |
| Fraction unbound (human) | 0.014<br>0.002 | 0<br>0.107 | 0.062<br>0.135 | 0.003<br>0 | 0<br>0.003 | 0 |
| BBB[3] permeability (Log BB) | -1.196<br>-1.319 | -1.123<br>-1.145 | -0.966<br>-0.994 | -0.934<br>-0.928 | -0.733<br>-1.042 | -1.487 |

| | | | | | | |
|---|---|---|---|---|---|---|
| CNS[4] permeability (LogPS) | -2.785 | -2.105 | -3.065 | -2.14 | -2.252 | -1.983 |
| | -1.992 | -3.163 | -3.141 | -2.598 | -2.037 | |
| Metabolism[a] | | | | | | |
| CYP3A4 substrate | Yes | Yes | Yes | Yes | Yes | Yes |
| | Yes | Yes | Yes | Yes | Yes | |
| CYP1A2 inhibitior | No | No | No | No | No | No |
| | No | No | No | No | No | |
| CYP2C19 inhibitior | Yes | Yes | Yes | Yes | Yes | Yes |
| | Yes | Yes | Yes | Yes | Yes | |
| CYP2C9 inhibitior | Yes | Yes | Yes | Yes | Yes | Yes |
| | Yes | Yes | Yes | Yes | Yes | |
| CYP2D6 inhibitior | No | No | No | No | No | No |
| | No | No | No | No | No | |
| CYP3A4 inhibitior | Yes | Yes | Yes | Yes | Yes | Yes |
| | Yes | Yes | Yes | Yes | Yes | |
| Elimination[a] | | | | | | |
| Total Clearance | 0.285 | 0.077 | -0.052 | -0.037 | 0.094 | -0.218 |
| | 0.132 | 0.024 | 0.269 | 0.128 | 0.285 | |
| Renal OCT2[5] substrate | No | No | No | No | No | No |
| | No | No | No | No | No | |

[1]human epithelial colorectal adenocarcinoma cell line; [2]steady state volume of distribution; [3] blood-brain barriers; [4]central nervous system; [5]organic cation transporter 2; [a]Parameter predicted by pkCSM web online tool; [b]Parameter predicted by SwissADME web online tool,

**Table S3**. *In silico* toxicity predictions of the **TSC1-TSC10** series & **sorafenib** by pkCSM.

| | **TSC1** / **TSC6** | **TSC2** / **TSC7** | **TSC3** / **TSC8** | **TSC4** / **TSC9** | **TSC5** / **TSC10** | **Sorafenib** |
|---|---|---|---|---|---|---|
| AMES[1] toxicity | No | No | No | No | No | No |
| | No | No | No | No | No | |
| Max. tolerated dose (human) (log mg/Kg/day) | 0.356 | 0.619 | 0.739 | 0.644 | 0.703 | 0.16 |
| | 0.545 | 0.77 | 0.592 | 0.142 | 0.597 | |
| hERG I inhibitor[2] | No | No | No | No | No | No |
| | No | No | No | No | No | |
| hERG II inhibitor[3] | Yes | Yes | Yes | Yes | Yes | Yes |
| | Yes | Yes | Yes | Yes | Yes | |
| Oral Rat Acute Toxicity (LD50) (mol/kg) | 2.842 | 2.877 | 2.787 | 2.891 | 2.878 | 2.239 |
| | 2.879 | 2.625 | 2.242 | 2.867 | 2.900 | |
| Oral Rat Chronic Toxicity (log mg/kg_bw_day) | 1.941 | 1.587 | 1.660 | 1.679 | 1.751 | 0.79 |
| | 1.517 | 1.815 | 2.209 | 1.954 | 1.609 | |
| Hepatotoxicity | Yes | Yes | Yes | Yes | Yes | Yes |
| | Yes | Yes | Yes | Yes | No | |
| Skin Sensitisation | No | No | No | No | No | No |

| | | | | | | |
|---|---|---|---|---|---|---|
| | No | No | No | No | No | |
| *T.pyriformis* toxicity (log µg/L) | 0.365 | 0.289 | 0.289 | 0.291 | 0.292 | 0.336 |
| | 0.288 | 0.287 | 0.285 | 0.357 | 0.29 | |
| Minnow toxicity (Log mM) | 0.323 | -0.751 | -1.02 | -1.319 | -1.21 | -0.128 |
| | -0.751 | -1.276 | 0.411 | -0.484 | -2.07 | |

[1]*S. typhimurium* reverse mutation assay; [2,3]inhibition of K channels encoded by hERG (human ether-a-go-go gene);

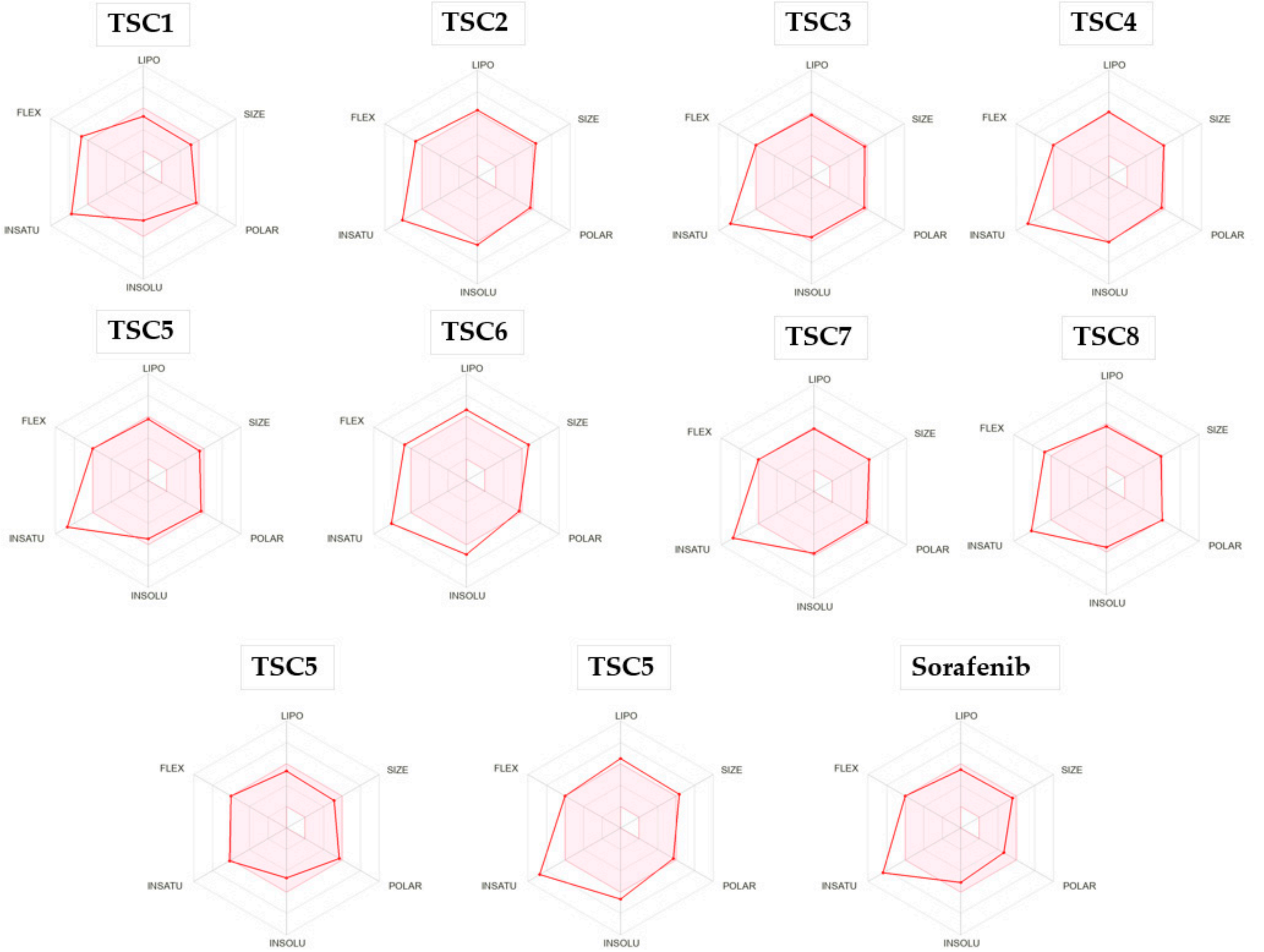


**Figure S43.** Graphical representation of the bioavailability radar for the series TSC1-TSC10 & sorafenib by SwissADME (The red area outlines the suitable range for oral obsrobtion); LIPO (lipophilicity, XLOGP3 = -7 < > +5.0), SIZE (Molecular Weight g/mol, SIZE = 150 < > 500); POLAR (Polarity,POLAR = 20 Å² < > 130 Å²); INSOLU (insolubility; Log S (ESOL) = -6 < > 0); INSATU (Insaturation; Fraction Csp³ = 0.25 < > 1); FLEX (Flexibility, Num. of rotatable bonds = 0 < > 9).

## 5. Molecular Docking Studies

**Table S4.** The 2D depictions of the interactions between the studied series TSC1-TSC10 and active site of VEGFR2

| TSC1 | TSC2 |
|---|---|
| 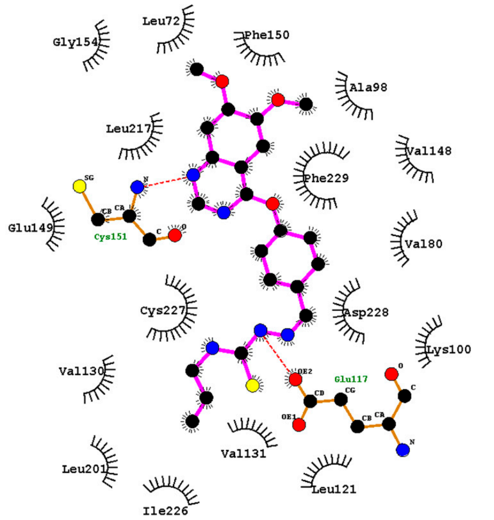 | 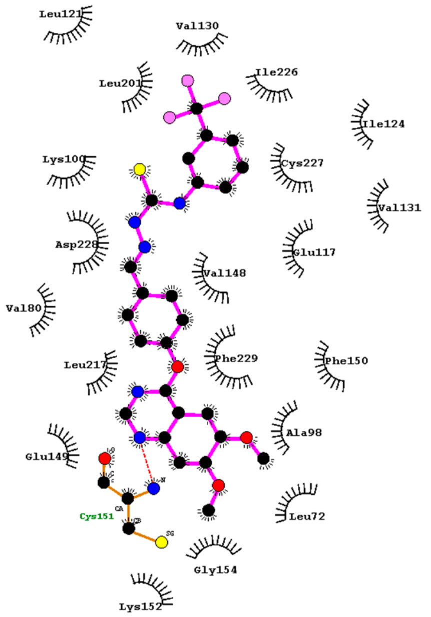 |
| TSC3 | TSC4 |
| 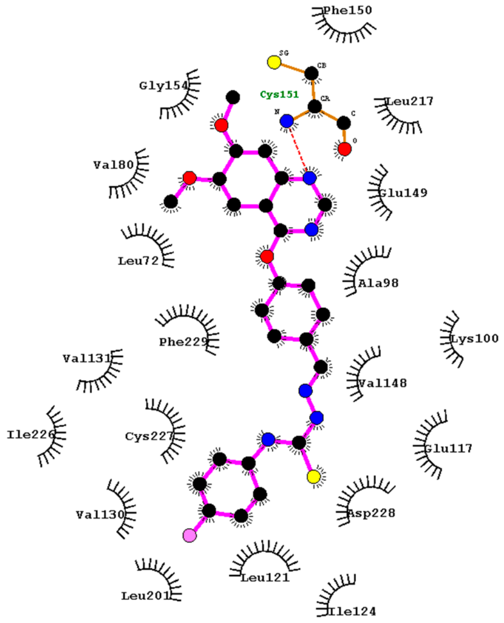 | 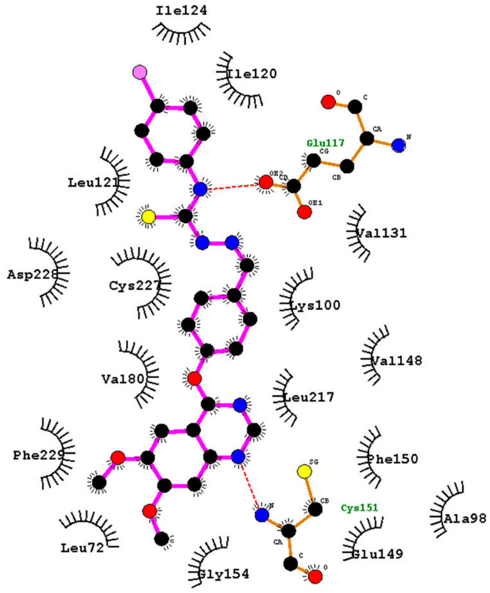 |
| TSC5 | TSC6 |

| | |
|---|---|
| 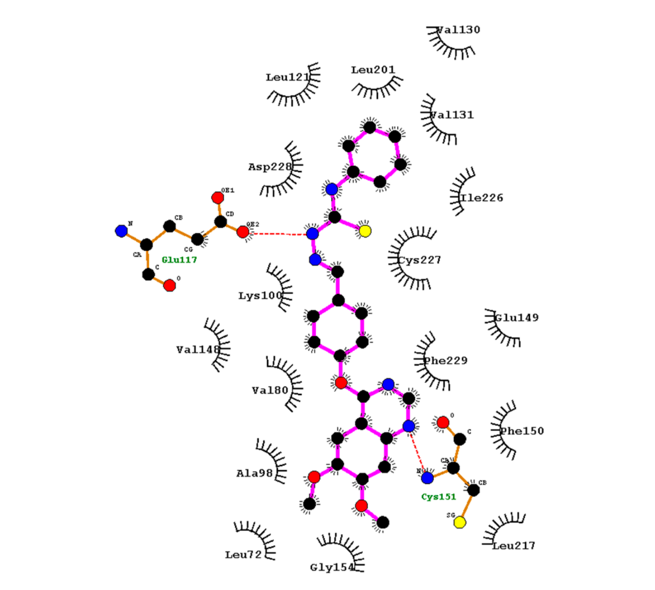  | 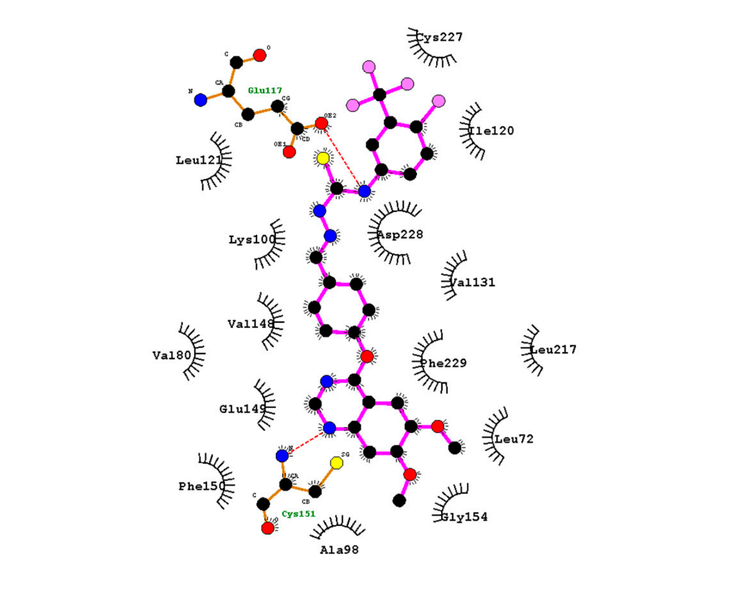  |
| **TSC7** | **TSC8** |
| 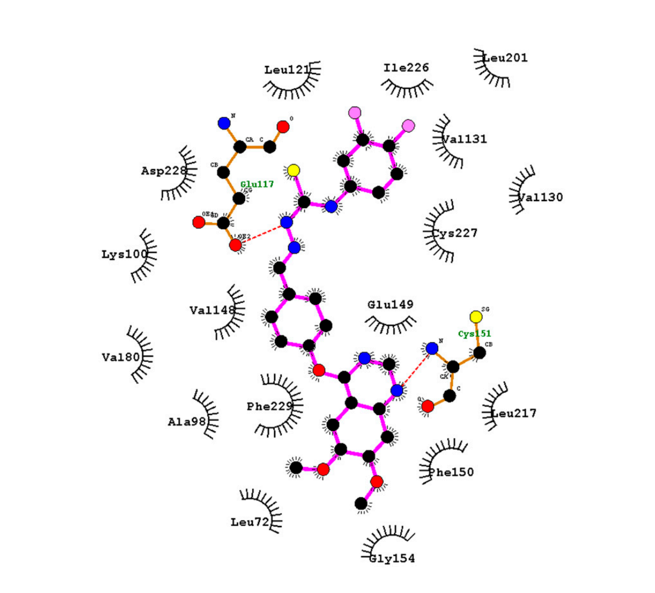  | 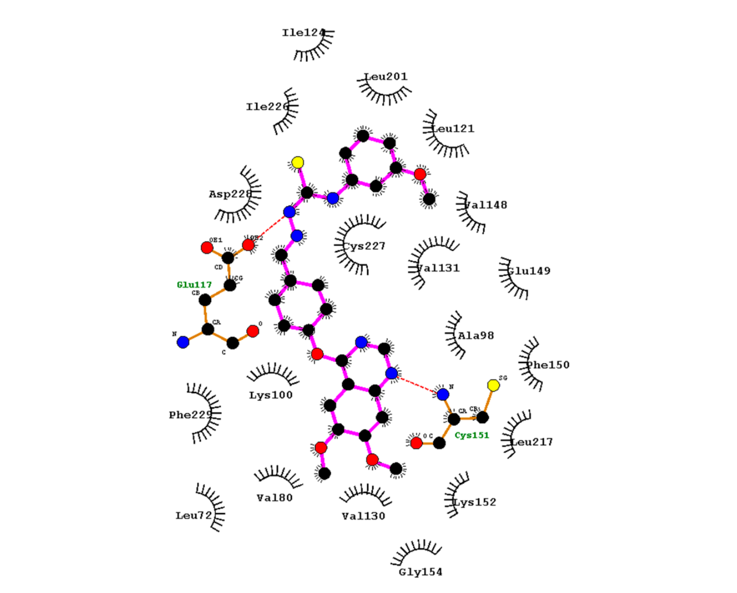  |
| **TSC9** | **TSC10** |

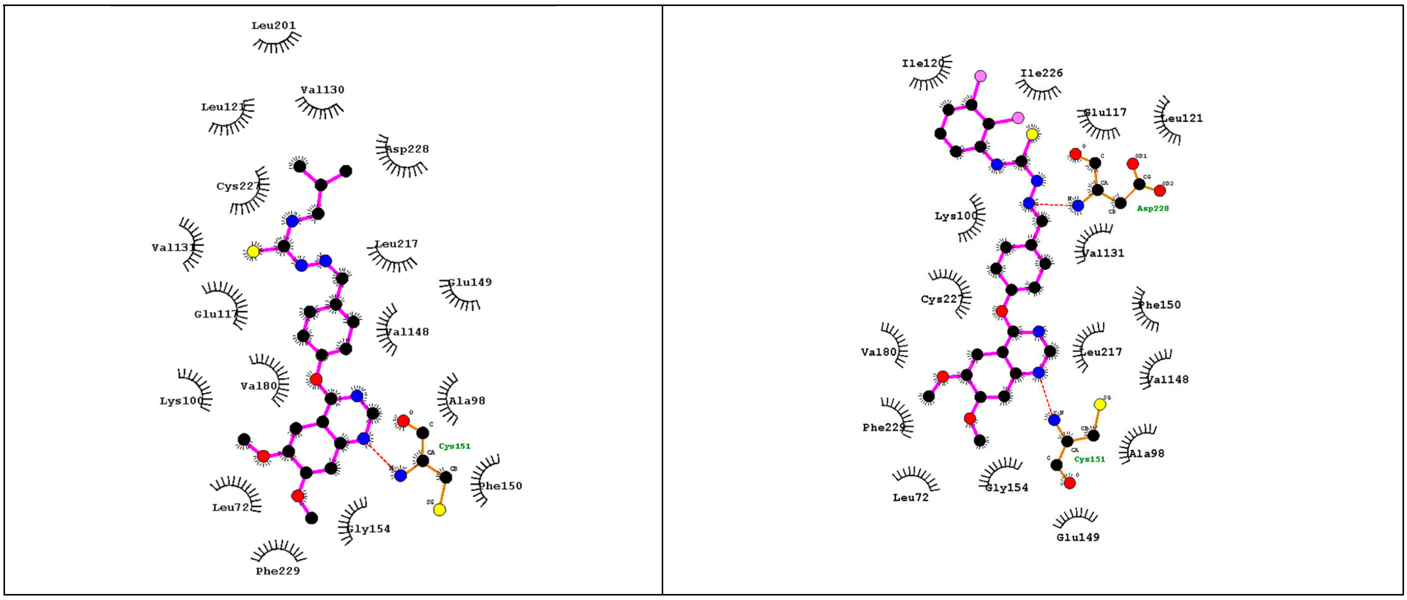


## 6. Density function theory (DFT) studies

**Table S5.** The DFT parameters of the **TSC1-TSC10** series & **sorafenib**.

| | TSC1 / TSC6 | TSC2 / TSC7 | TSC3 / TSC8 | TSC4 / TSC9 | TSC5 / TSC10 | Sorafenib |
|---|---|---|---|---|---|---|
| **Frontal Molecular Orbitals (FMOs) Analysis** | | | | | | |
| HOMO (eV) | -5.51 | -5.89 | -5.73 | -5.82 | -5.68 | -6.04 |
| | -6.02 | -5.86 | -5.66 | -5.48 | -5.92 | |
| LUMO (eV) | -1.73 | -2.00 | -1.83 | -1.92 | -1.79 | -1.45 |
| | -2.14 | -1.95 | -1.77 | -1.68 | -2.06 | |
| Energy gap (eV) | 3.78 | 3.89 | 3.90 | 3.90 | 3.89 | 4.59 |
| | 3.88 | 3.91 | 3.89 | 3.80 | 3.86 | |
| **Chemical Reactivity Descriptors** | | | | | | |
| Ionization potential (I) (eV) | 5.51 | 5.89 | 5.73 | 5.82 | 5.68 | 6.04 |
| | 6.02 | 5.86 | 5.66 | 5.48 | 5.92 | |
| Electronic affinity (A) (eV) | 1.73 | 2.00 | 1.83 | 1.92 | 1.79 | 1.45 |
| | 2.14 | 1.95 | 1.77 | 1.68 | 2.06 | |
| Chemical hardness (η) (eV) | 1.89 | 1.95 | 1.95 | 1.95 | 1.95 | 2.30 |
| | 1.94 | 1.96 | 1.95 | 1.90 | 1.93 | |
| Chemical potential (μ) (eV) | -3.62 | -3.95 | -3.78 | -3.87 | -3.74 | -3.75 |
| | -4.08 | -3.91 | -3.72 | -3.58 | -3.99 | |
| Global softness (S) (eV) | 0.95 | 0.97 | 0.98 | 0.98 | 0.97 | 1.15 |
| | 0.97 | 0.98 | 0.97 | 0.95 | 0.97 | |
| Nucleophilicity index (N) ($eV^{-1}$) | 0.29 | 0.25 | 0.27 | 0.26 | 0.28 | 0.33 |
| | 0.23 | 0.26 | 0.28 | 0.30 | 0.24 | |
| | 3.47 | 4.00 | 3.66 | 3.84 | 3.59 | 3.06 |

| | | | | | | |
|---|---|---|---|---|---|---|
| Electrophilicity index (ω) (eV) | 4.29 | 3.90 | 3.55 | 3.37 | 4.12 | |
| Additional electronic charges (ΔN) | 1.92<br>2.10 | 2.03<br>2.00 | 1.94<br>1.91 | 1.98<br>1.88 | 1.92<br>2.07 | 1.63 |
| | | | | | | |
| Dipole moment (Debye) | 4.94<br>5.19 | 5.36<br>4.36 | 3.92<br>3.87 | 3.56<br>5.23 | 4.83<br>6.13 | 6.68 |

**Table S6.** HOMO-LUMO orbital distribution and MEP for the series **TSC1-TSC9**.

| Compound | HOMO distribution | LUMO distribution | MEM |
|---|---|---|---|
| **TSC1** | 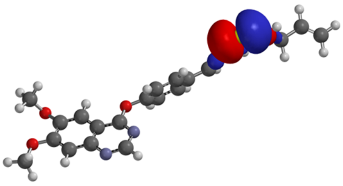 | 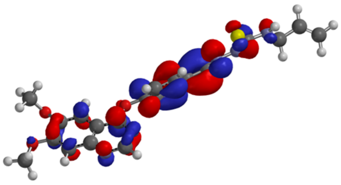 | 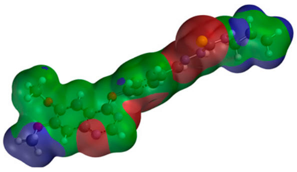 |
| **TSC2** | 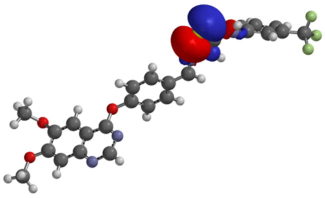 | 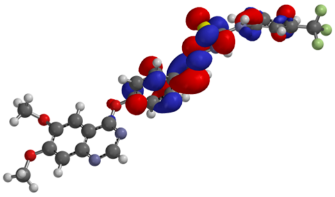 | 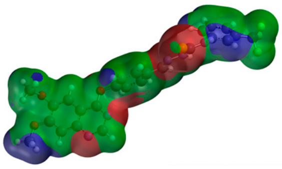 |
| **TSC3** | 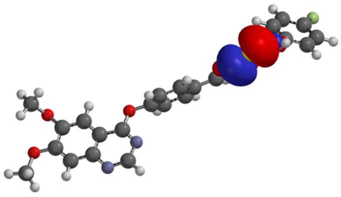 | 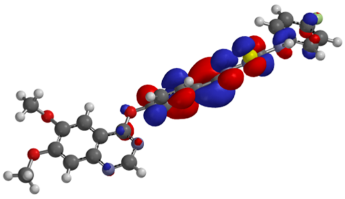 | 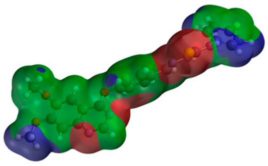 |
| **TSC4** | 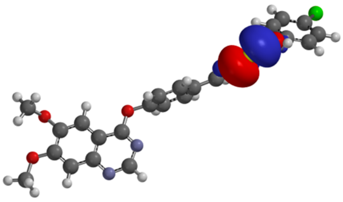 | 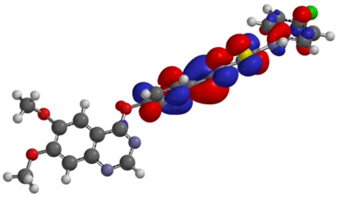 | 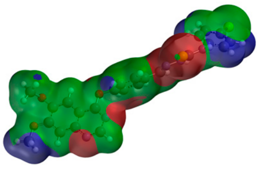 |
| **TSC5** | 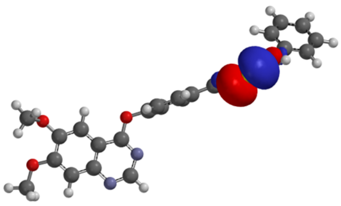 | 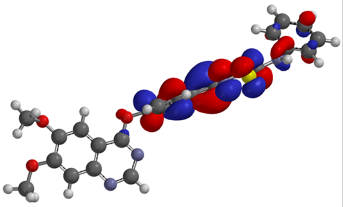 | 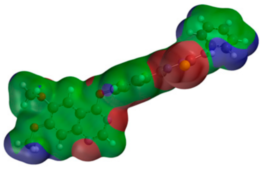 |
| **TSC6** | 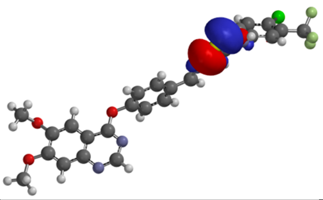 | 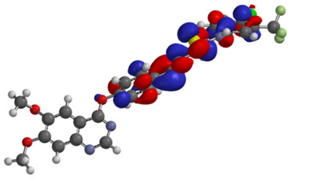 | 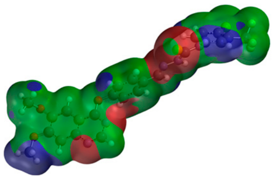 |

| | | | |
|---|---|---|---|
| TSC7 | 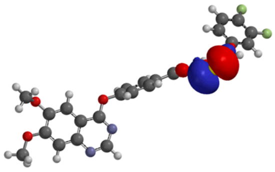 | 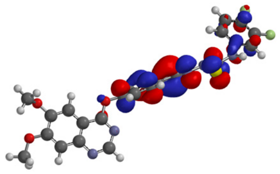 | 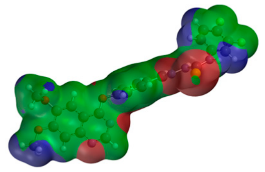 |
| TSC8 | 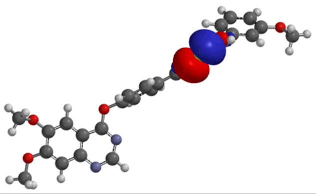 | 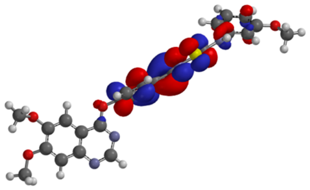 | 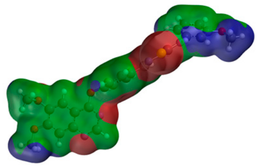 |

## 7. Molecular Dynamics (MD) studies

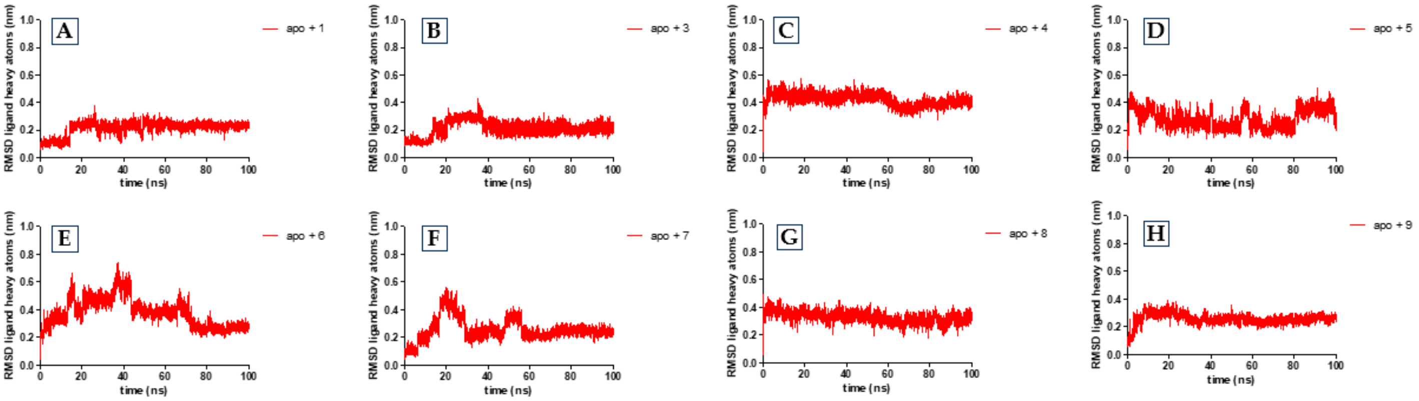


**Figure S44.** Graphical representation of RMSD-Ligand of the ligand-VEGFR2 complex during the 100 ns simulation for TSC1 (A); TSC3 (B); TSC4 (C); TSC5 (D); TSC6 (E); TSC7 (F); TSC8 (G); TSC9 (H);

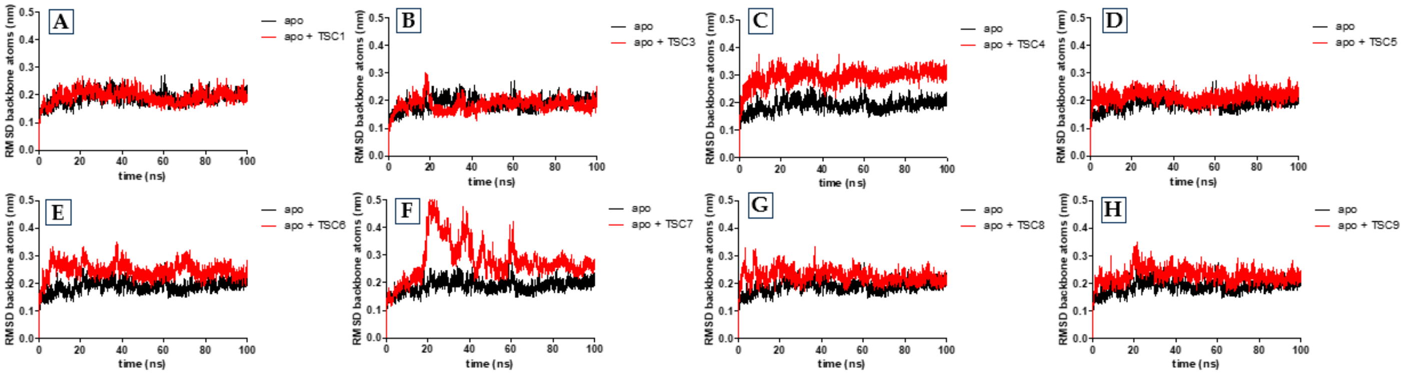


**Figure S45.** Graphical representation of RMSD-Protein of the ligand-VEGFR2 complex during the 100 ns simulation for TSC1 (A); TSC3 (B); TSC4 (C); TSC5 (D); TSC6 (E); TSC7 (F); TSC8 (G); TSC9 (H);

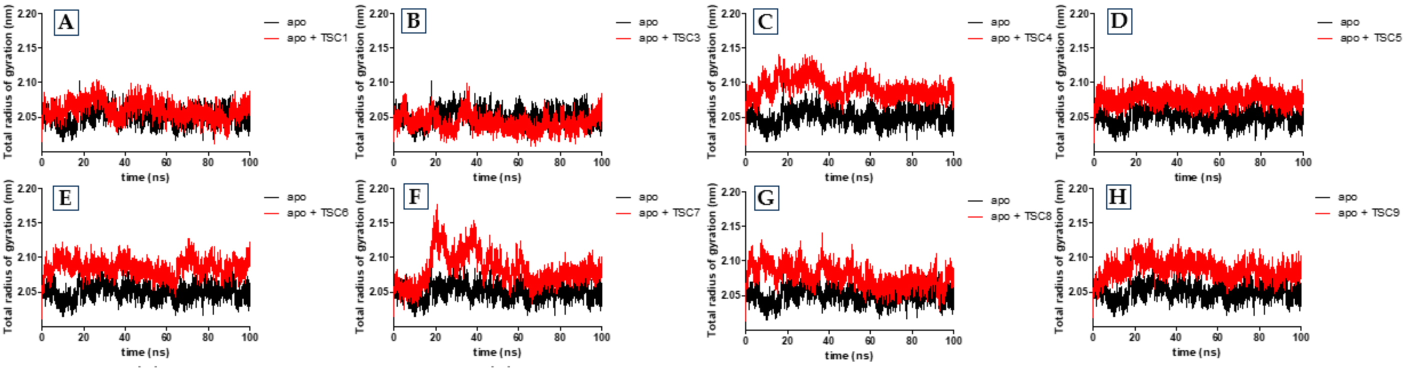


**Figure S46.** Graphical representation of Radius of gyration of the ligand-VEGFR2 complex during the 100 ns simulation for TSC1 (A); TSC3 (B); TSC4 (C); TSC5 (D); TSC6 (E); TSC7 (F); TSC8 (G); TSC9 (H);

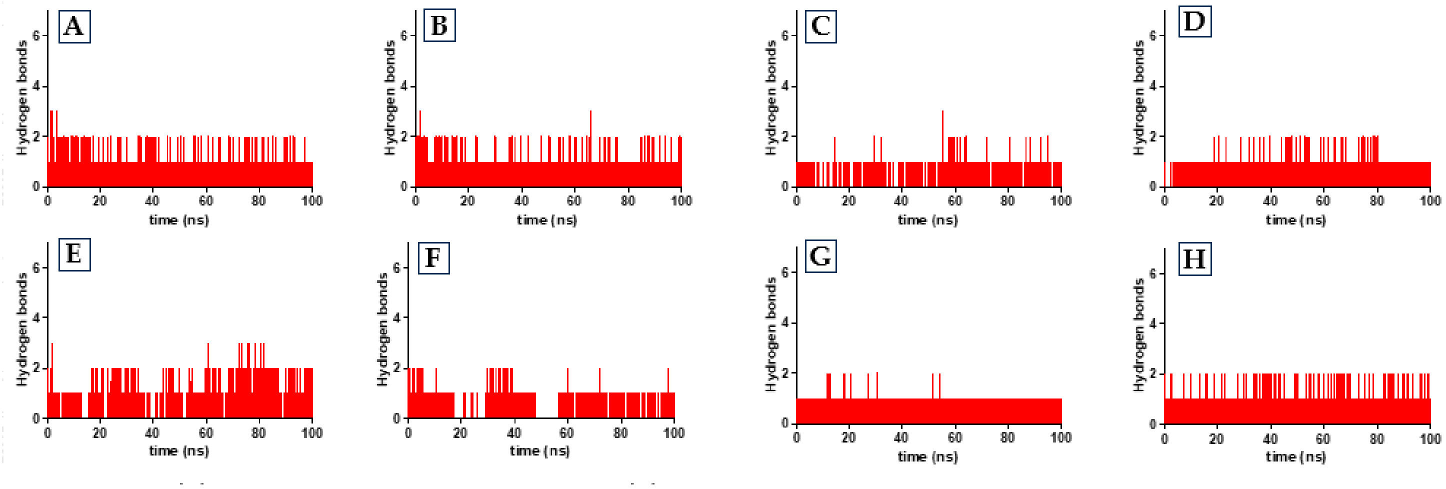


**Figure S47.** Graphical representation of the number of H-bonds of the ligand-VEGFR2 complex during the 100 ns simulation for TSC1 (A); TSC3 (B); TSC4 (C); TSC5 (D); TSC6 (E); TSC7 (F); TSC8 (G); TSC9 (H);

**Table S7.** The depiction of the pose of the ligands (**TSC1-TSC10**) at the beginning of the simulation (0 ns, black skeleton) and at the end of the simulation (100 ns, magenta skeleton) within the active site of VEGFR2

| TSC1 | TSC2 |
|---|---|
| 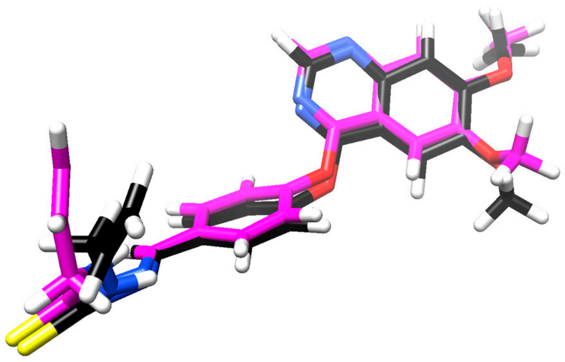 | 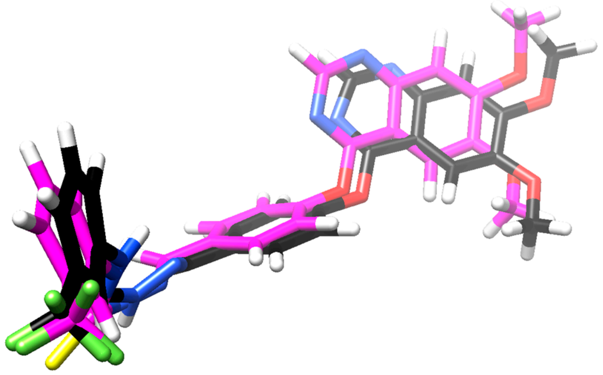 |
| **TSC3** | **TSC4** |

| | |
|---|---|
| 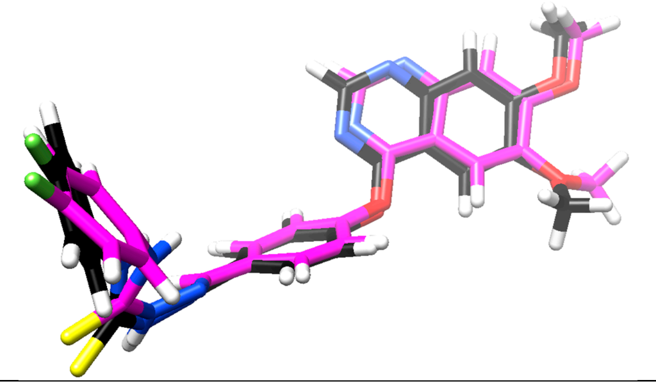 | 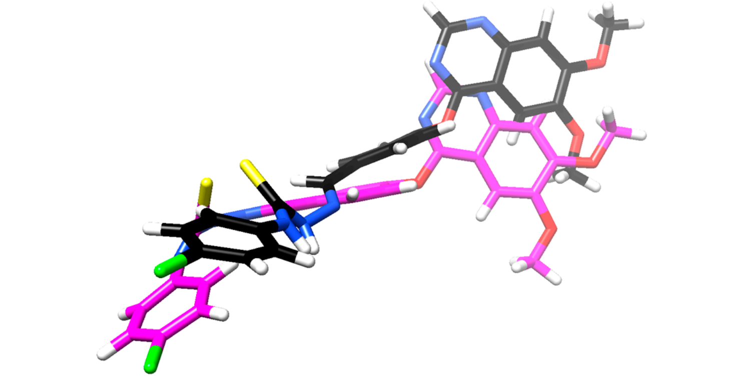 |
| **TSC5** | **TSC6** |
| 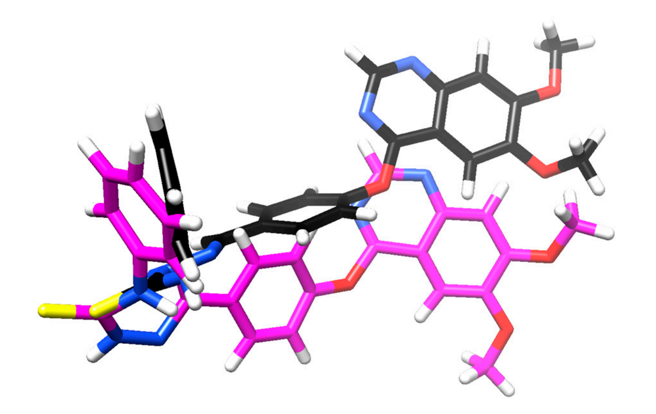 | 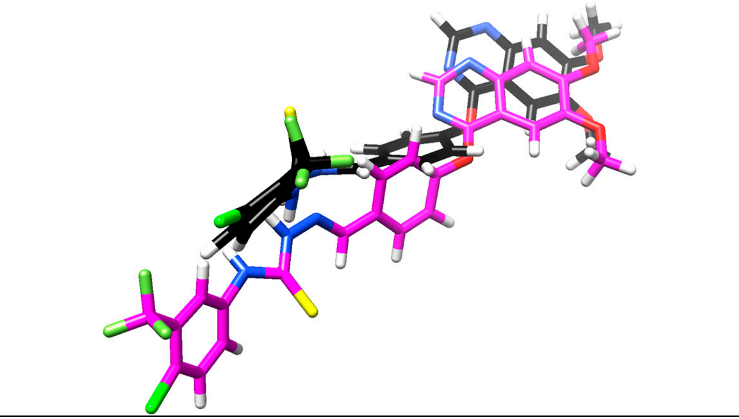 |
| **TSC7** | **TSC8** |
| 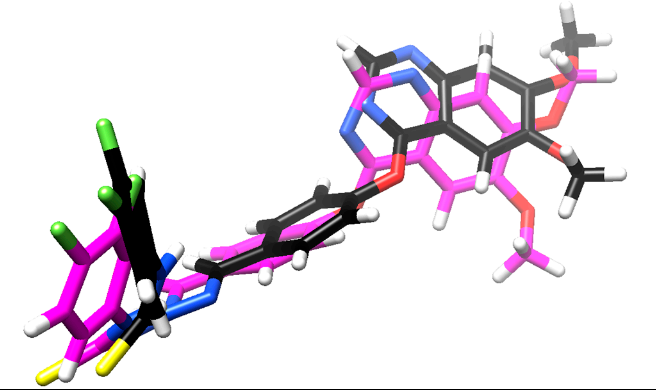 | 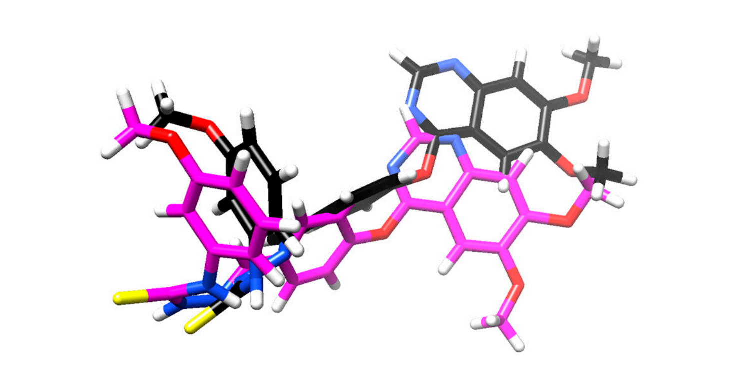 |
| **TSC9** | **TSC10** |
| 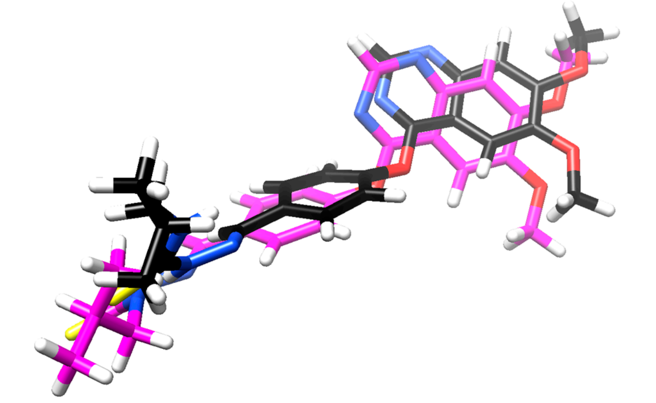 | 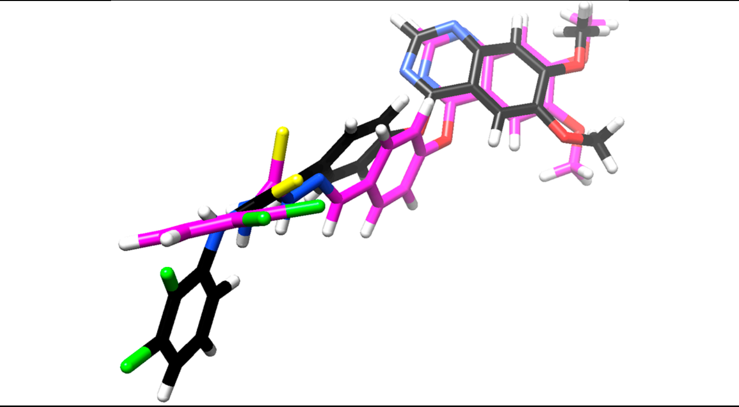 |

**Table S8.** H-bonds decomposition in the complexes of **TSC1-TSC10** and **sorafenib** with VEGFR2 during the 100 ns simulations and the encounter frequency (%).

| | Number of H-bonds interactions (% of total interactions) | | | | | |
|---|---|---|---|---|---|---|
| | **Lys868** | **Glu885** | **Cys919** | **Asp1046** | **Other amino acids** | **Total H-Bonds** |
| **TSC1** | 822 (8.22%) | 45 (0.45%) | 1374 (13.74%) | 38 (0.38%) | - | 2279 |
| **TSC2** | 835 (8.35%) | 766 (7.66%) | 1659 (16.59%) | 49 (0.49%) | - | 3309 |
| **TSC3** | 404 (4.04%) | 313 (3.13%) | 1087 (10.87%) | 23 (0.23%) | - | 1827 |
| **TSC4** | - | 346 (3.46%) | - | 1 (0.01%) | **Pro812** (204/2.04%); **Leu813** (3/0.03%); **Ile1025** (15/0.15%); **Arg1027** (28/0.38%); **Arg1051** (101/1.10%) | 717 |
| **TSC5** | - | 15 (1.5%) | 158 (1.58%) | 2481 (24.81%) | **Leu813** (67/6.7%); **Asp814** (70/7%); **Ile892** (6/0.06%); **Leu1019** (21/2.1%); **Cys1024** (61/6.1%) **Ile1025** (24/2.4%); | 2654 |
| **TSC6** | - | 28 (2.8%) | 141 (1.41%) | 1953 (19.53%) | **Val899** (15/1.5%) | 2374 |
| **TSC7** | 21 (0.21%) | 328 (3.28%) | 660 (6.60%) | 26 (0.26%) | **Arg1051** (20/2,0%) | 1050 |
| **TSC8** | - | 7 (0.07%) | - | 3683 (36.83%) | - | 3710 |
| **TSC9** | 971 (9.71%) | 14 (0.14%) | 1012 (10.12%) | - | - | 1997 |
| **TSC10** | - | 1254 (12.54%) | 1512 (15.12%) | 1376 (13.76%) | **Leu1019** (96/0.96%); **Cys1024** (5/0.05%); **His1026** (36/0.36%); **Arg1027** (50/0.50%); **Ile1044** (95/0.95%); | 4424 |
| **Sorafenib** | - | 3825 (38.25%) | 178 (1.78%) | 5833 (58.33%) | **Val899** (4/0.04%); **Cys1024** (13/0.13%); **His1026** (13/0.13%); | 9866 |

**Table S9.** H-bonds decomposition in quarters generated in the complexes of **TSC2, TSC10** and **sorafenib** with VEGFR2 during the 100 ns simulations and the encounter frequency (%) for the most relevant AAs

| **Compound** | Number of H-bonds interactions | | | | |
|---|---|---|---|---|---|
| | **Time (ns)** | **Lys868** | **Glu885** | **Cys919** | **Asp1046** |
| **TSC10** | 0-24 | - | - | 264 | 66 |
| | 25-49 | - | 362 | 459 | 376 |
| | 50-74 | - | 515 | 424 | 457 |
| | 75-100 | - | 377 | 364 | 476 |
| **TSC2** | 0-24 | 319 | 127 | 347 | 16 |
| | 25-49 | 133 | 265 | 431 | 16 |
| | 50-74 | 144 | 149 | 437 | 11 |
| | 75-100 | 239 | 225 | 444 | 6 |
| **Sorafenib** | 0-24 | - | 864 | 50 | 1344 |
| | 25-49 | - | 1008 | 41 | 1495 |
| | 50-74 | - | 960 | 55 | 1505 |

| | | | | | |
|---|---|---|---|---|---|
| | 75-100 | - | 993 | 32 | 1487 |

## 8. MMPB/SA energy decomposition

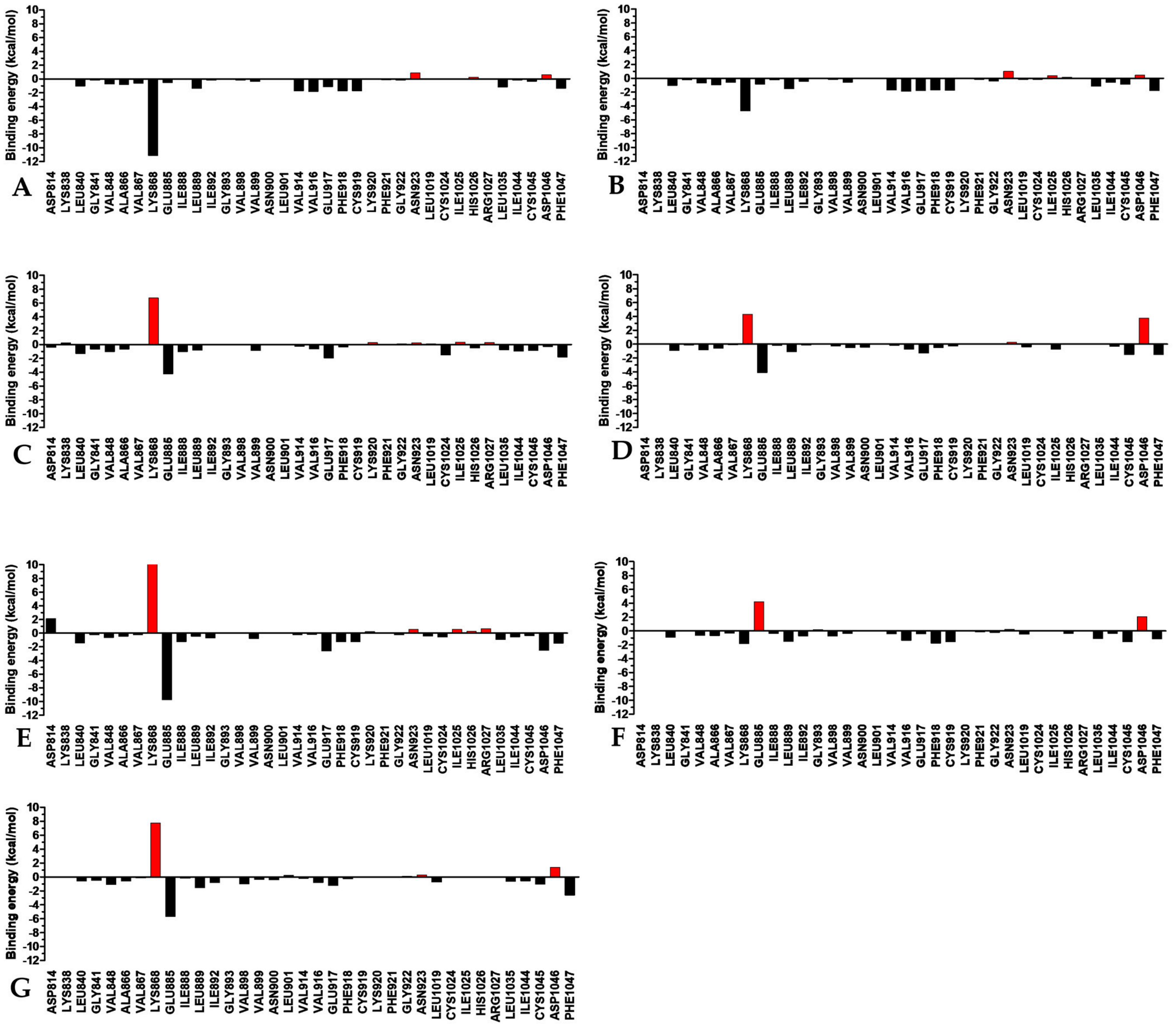


**Figure S48.** Free energy decomposition for all the AAs in the 5Å proximity of the ligand (A – **TSC1**, B –**TSC3**, C – **TSC4**, D – **TSC5,** E –**TSC6**, F – **TSC7**, G – **TSC8**) during the last 2500 frames of the MD simulation.
